# Taxane-induced conformational changes in the microtubule lattice activate GEF-H1-dependent RhoA signalling

**DOI:** 10.1038/s41556-026-02079-4

**Published:** 2026-09-24

**Authors:** Joyce C. M. Meiring, Varsha Mahapatra, Ruijie Liu, Molly S. C. Gravett, Daan Morren, Harriet A. J. Saunders, Sung Ryul Choi, Andressa Pelster José, Adela Karhanova, Ioanna Metallidou, Alex Moore, Marèl F. M. Spoelstra, Ilya S. Grigoriev, Matteo Giono, Saishree S. Iyer, Samantha J. Stehbens, J. Fernando Díaz, Zdenek Lansky, Kelly E. Stecker, Michel O. Steinmetz, Stuart C. Howes, Lukas C. Kapitein, Anna Akhmanova

**Affiliations:** 1 Cell Biology, Neurobiology and Biophysics, Department of Biology, Faculty of Science, Utrecht University, Utrecht, the Netherlands; 2 Institute for Molecular Bioscience, The University of Queensland, Brisbane, Queensland Australia; 3 Australian Institute for Bioengineering and Nanotechnology, The University of Queensland, Brisbane, Queensland Australia; 4 Structural Biochemistry, Department of Chemistry, Bijvoet Centre for Biomolecular Research, Utrecht University, Utrecht, the Netherlands; 5 PSI Center for Life Sciences, Villigen PSI, Villigen, Switzerland; 6 Institute of Biotechnology, Czech Academy of Sciences, BIOCEV, Vestec, Czechia; 7 Department of Cell Biology, Faculty of Science, Charles University, Prague, Czech Republic; 8 Biomolecular Mass Spectrometry and Proteomics, Bijvoet Center for Biomolecular Research and Utrecht Institute for Pharmaceutical Sciences, Utrecht University, Utrecht, the Netherlands; 9 Netherlands Proteomics Center, Utrecht, the Netherlands; 10 School of Chemistry and Molecular Biosciences, The University of Queensland, Brisbane, Queensland Australia; 11 BICS Unit, Centro de Investigaciones Biológicas Margarita Salas, Consejo Superior de Investigaciones Científicas CIB-CSIC, Madrid, Spain; 12 Biozentrum, University of Basel, Basel, Switzerland; 13 Center for Living Technologies, Eindhoven-Wageningen-Utrecht Alliance, UMC Utrecht, Utrecht, the Netherlands

**Keywords:** Microtubules, Actin, Cancer

## Abstract

Taxanes are widely used chemotherapeutic agents that perturb cell division. They also exert effects during interphase, but the underlying mechanisms are poorly understood. Here we show that taxanes activate RhoA signalling and induce actin remodelling by releasing the RhoA activator GEF-H1 from microtubules. This taxane-induced release of GEF-H1 occurs rapidly, is independent of tubulin post-translational modifications, and can be recapitulated using purified proteins. In vitro reconstitution assays combined with analyses of microtubule structure revealed that microtubule binding by GEF-H1 is inhibited by microtubule-stabilizing ligands that expand the microtubule lattice, such as taxanes and GMPCPP, but not by others, including GTPγS and discodermolide, which stabilize a compacted microtubule lattice. Our findings demonstrate that alterations in microtubule lattice conformation can activate key signalling pathways, offering insights into the mode of action of taxanes and the possible origins of their side effects.

## Abstract (web summary)

Meiring, Mahapatra et al. show that expanded microtubule lattice conformations, such as those induced by small-molecule taxanes, can lead to the release of GEF-H1 and thus induce RhoA activation.

## Main

Microtubule-targeting agents (MTAs) are widely used to treat different cancers^1–3^. MTAs are divided into microtubule-destabilizing agents, including colchicine and vinca alkaloids, and microtubule-stabilizing agents, such as taxanes^4–6^. Both types of compound strongly perturb cell division by disrupting spindle formation and microtubule–kinetochore attachments and either triggering spindle assembly checkpoint, which blocks cell-cycle progression, or by inducing chromosome missegregation^3,7–10^. Although the perturbation of mitosis has long been regarded as the explanation for the anti-tumour effects of MTAs, drugs specifically targeting the formation of the mitotic spindle, such as kinesin-5 inhibitors, have shown disappointing results in the clinic, suggesting that the interphase effects of MTAs probably contribute to their clinical efficacy^11,12^. Moreover, there are indications that interphase pathways induced by MTAs may promote cancer cell death^13,14^, but the underlying molecular mechanisms are currently unknown. Finally, MTAs strongly perturb cell migration, important for cancer metastasis, by affecting cell morphology, polarity, intracellular transport and the crosstalk between microtubules and the actin cytoskeleton^3,7,9,10,15,16^.

The major interphase effect of microtubule-destabilizing agents is the activation of signalling by the small GTPase RhoA^17^. This effect depends on the RhoA activator guanine nucleotide exchange factor H1 (GEF-H1), which is sequestered on microtubules in an inactive form, but becomes active upon release from microtubules^18–22^. GEF-H1 function is important for cell migration, matrix invasion and immune cell function^19,21,23–26^, and GEF-H1 expression has been reported to be increased in some cancers^27–29^.

Sequestration of GEF-H1 on microtubules can effectively regulate its activity, because RhoA activation is primarily restricted to the plasma membrane^30^. Because taxanes stabilize microtubules, they would not be expected to affect GEF-H1 activity. However, microtubule stabilization can potentially affect microtubule-associated components of signalling cascades by altering their turnover and tubulin post-translational modifications^31^. Previous studies have suggested that GEF-H1 avoids acetylated microtubules^32,33^. The underlying mechanism is likely to be indirect, because GEF-H1 binds to the outer microtubule surface^22^, whereas acetylation occurs on the luminal microtubule surface^34–36^. Acetylation in interphase cells typically occurs on stable microtubules^37,38^, and stable microtubules were recently found to have a distinct, expanded lattice conformation^39^. This expanded conformation, characterized by an increased longitudinal spacing between tubulin dimers, is also induced by taxanes^39–41^. Hence, we set out to investigate whether taxanes can trigger GEF-H1-mediated RhoA activation through direct modification of the microtubule lattice. Using a combination of cell biology and in vitro reconstitution assays, we show that taxanes indeed can rapidly activate RhoA by releasing GEF-H1 from microtubules through a direct mechanism that involves an alteration of microtubule lattice conformation.

## Results

### Taxanes activate RhoA through GEF-H1

**Fig. 1:**
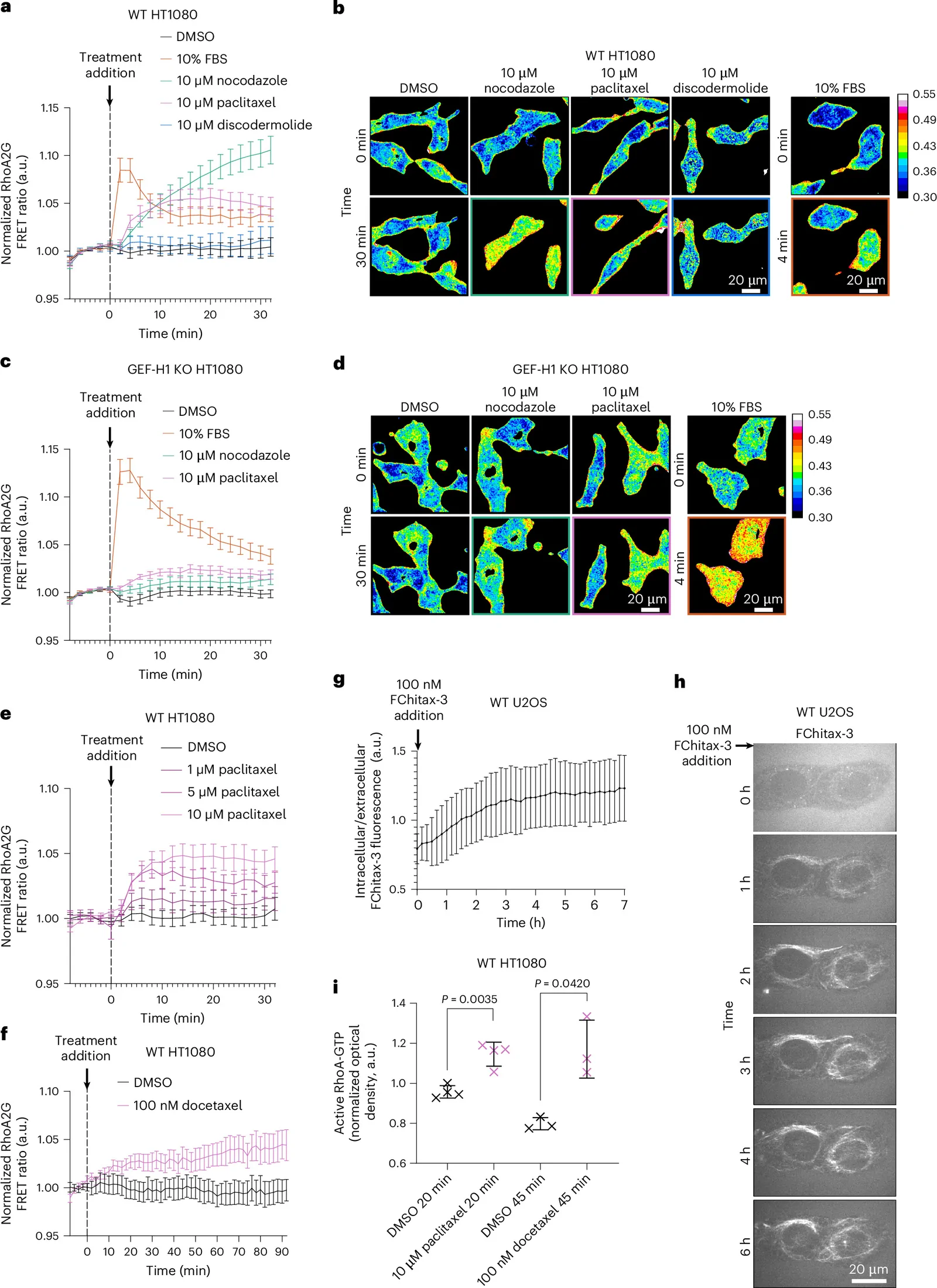
Paclitaxel activates RhoA in cells in a GEF-H1 dependent manner. **a**–**d**, Serum-starved HT1080 wild-type (WT) (**a**,**b**) or GEF-H1 knockout (KO) (**c**,**d**) cells stably expressing RhoA2G FRET sensor were first imaged live for 8 min without treatment to establish a baseline, then treated with either paclitaxel, nocodazole, discodermolide, serum (10% fetal calf serum) as a positive control or DMSO as a solvent control at time = 0 min and imaged for another 32 min. **a**,**c**, Graphs showing mean ± 95% confidence interval (WT: DMSO *n* = 42, FBS *n* = 41, nocodazole *n* = 43, paclitaxel *n* = 49, discodermolide *n* = 32; GEF-H1 KO: DMSO *n* = 48, FBS *n* = 45, nocodazole *n* = 48, paclitaxel *n* = 50 cells over three independent experiments) of the experimental means of three independent experiments. a.u., arbitary units. Data were normalized to the average baseline collected before treatment. **b**,**d**, Representative stills of the RhoA2G FRET ratio of cells before (time = 0 min) and after (time = 30 min or 4 min) treatment. RhoA2G FRET sensor fluorescence was used to create masks of the cells, and all signals outside the mask were cleared. **e**,**f**, Serum-starved HT1080 WT cells stably expressing RhoA2G FRET sensor were first imaged live for 8 min without treatment to establish a baseline, then treated with either 1, 5 or 10 µM paclitaxel or 100 nM docetaxel or DMSO as a solvent control and imaged for a further 32 min (**e**) or 92 min (**f**). FRET ratios were normalized to the average of the baseline collected before treatment addition. Plots show the mean ± 95% confidence interval (**e**: DMSO, 1 µM paclitaxel, 5 µM paclitaxel *n* = 30, 10 µM paclitaxel *n* = 37; **f**: DMSO *n* = 27, docetaxel *n* = 32 cells over three independent experiments). **g**,**h**, U2OS cells were imaged on a spinning disc microscope from the moment fluorescent taxane FChitax-3 was added to the cell medium for 7 h. **g**, Plot showing mean ± 95% confidence interval of the ratio of intracellular/extracellular FChitax-3 fluorescence (*n* = 5, from one representative experiment). **h**, Representative frames showing FChitax-3 accumulating on microtubules in cells from one experiment. **i**, Serum-starved HT1080 cells were treated with DMSO or MTAs, as shown, for 20 or 45 min before collecting lysates and quantifying active RhoA-GTP via a RhoA G-LISA assay; plots show the mean ± 95% confidence interval of the experimental means (DMSO and paclitaxel 20 min, *n* = 4 experiments, DMSO and docetaxel 45 min, *n* = 3 experiments). A two-tailed Welch’s *t*-test was used to test for statistical significance. Source data

**Extended Data Fig. 1:**
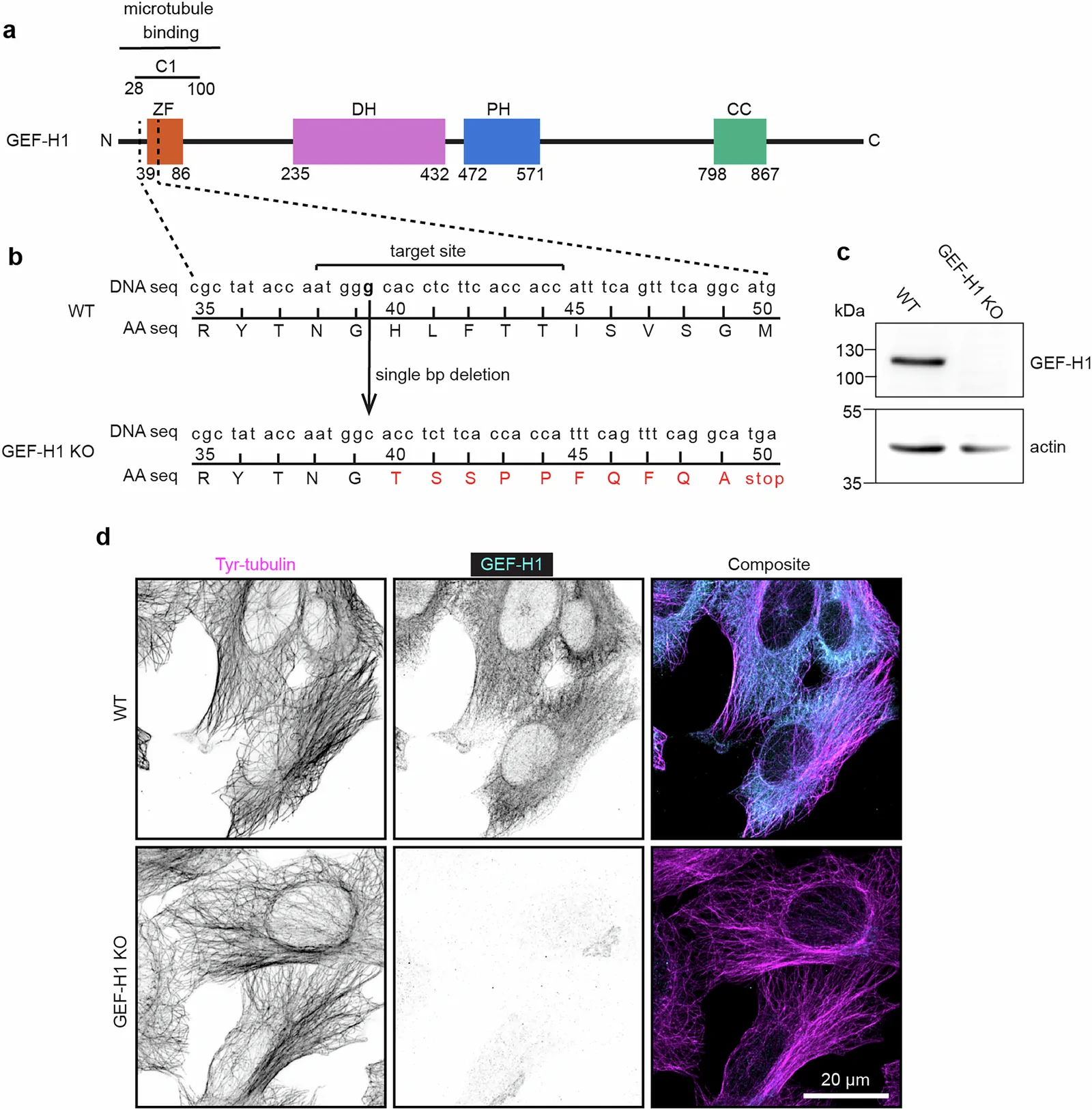
Characterization of HT1080 GEF-H1 knockout (KO) cell line. (**a**) Map of GEF-H1 protein domains, showing Zinc Finger (ZF), Dbl homology domain (DH), Pleckstrin homology domain (PH), Coiled Coil (CC) adapted from the Uniprot database, and the C1 microtubule binding domain described in^22^. (**b**) Scheme showing the location of the single DNA base pair (bp) deletion in the *ARHGEF2* (GEF-H1) gene of GEF-H1 KO cells, verified by sequencing. (**c**) Western blot of WT and GEF-H1 KO HT1080 lysates blotted for GEF-H1 and actin as a loading control. (**d**) WT and GEF-H1 KO HT1080 cells fixed and stained for GEF-H1 and tyrosinated α-tubulin.

To measure RhoA activation, we used HT1080 fibrosarcoma cells stably expressing the RhoA2G Förster resonance energy transfer (FRET) sensor^42^. For better reproducibility, we serum-starved cells to lower baseline RhoA activation, because lysophosphatidic acid in serum is a potent inducer of RhoA signalling^43^. As expected, RhoA activity was low in serum-starved cells, but showed a rapid transient increase with serum addition (Fig. 1a,b). The microtubule-destabilizing agent nocodazole (10 μM), which was used as a positive control for GEF-H1 activation^18^, caused complete microtubule disassembly and induced a slower, but persistent increase in RhoA activity (Fig. 1a,b). A similar, but weaker RhoA activation was also triggered by the addition of paclitaxel (10 μM; Fig. 1a,b). The effects of both nocodazole and paclitaxel, but not the effect of serum on RhoA activity, were largely suppressed in GEF-H1 knockout cells (Fig. 1c,d and Extended Data Fig. 1a–d), indicating that both MTAs activate RhoA through GEF-H1. However, GEF-H1 knockout cells could still trigger RhoA in response to serum addition, indicating that serum factors such as lysophosphatidic acid^44^ use alternative intermediates to activate RhoA. Interestingly, discodermolide, a taxane site microtubule stabilizer that, unlike taxanes, does not expand the microtubule lattice (refs. ^41,45,46^, and see below), did not induce RhoA activation (Fig. 1a,b).

In the short-term (30 min) assays described above, paclitaxel-induced activation of RhoA was weaker than that triggered by nocodazole. A compound concentration of at least 5 µM was needed to induce a substantial effect (Fig. 1a,e), whereas taxane concentrations in the plasma of patients with cancer during chemotherapy reach concentrations of between 0.1 and 1 µM (refs. ^47,48^). Importantly, docetaxel, a taxane with a higher affinity to microtubules^46^, and which is often used for cancer chemotherapy^49^, could activate RhoA signalling at a concentration of even 100 nM (Fig. 1f). The magnitude of RhoA activation by docetaxel increased over time (Fig. 1f), probably due to a slow but persistent accumulation of the drug. The idea that taxanes can gradually accumulate in cells at low concentrations was supported by 7-h-long live imaging of cell accumulation of a fluorescent taxane, Fchitax-3 (Fig. 1g,h). RhoA activation with 10 µM paclitaxel or 100 nM docetaxel was also confirmed using a RhoA G-LISA assay (Fig. 1i). Together, these results demonstrate that taxanes can trigger GEF-H1-mediated RhoA activation at clinically relevant concentrations.

### Paclitaxel causes GEF-H1-dependent actin remodelling

**Fig. 2:**
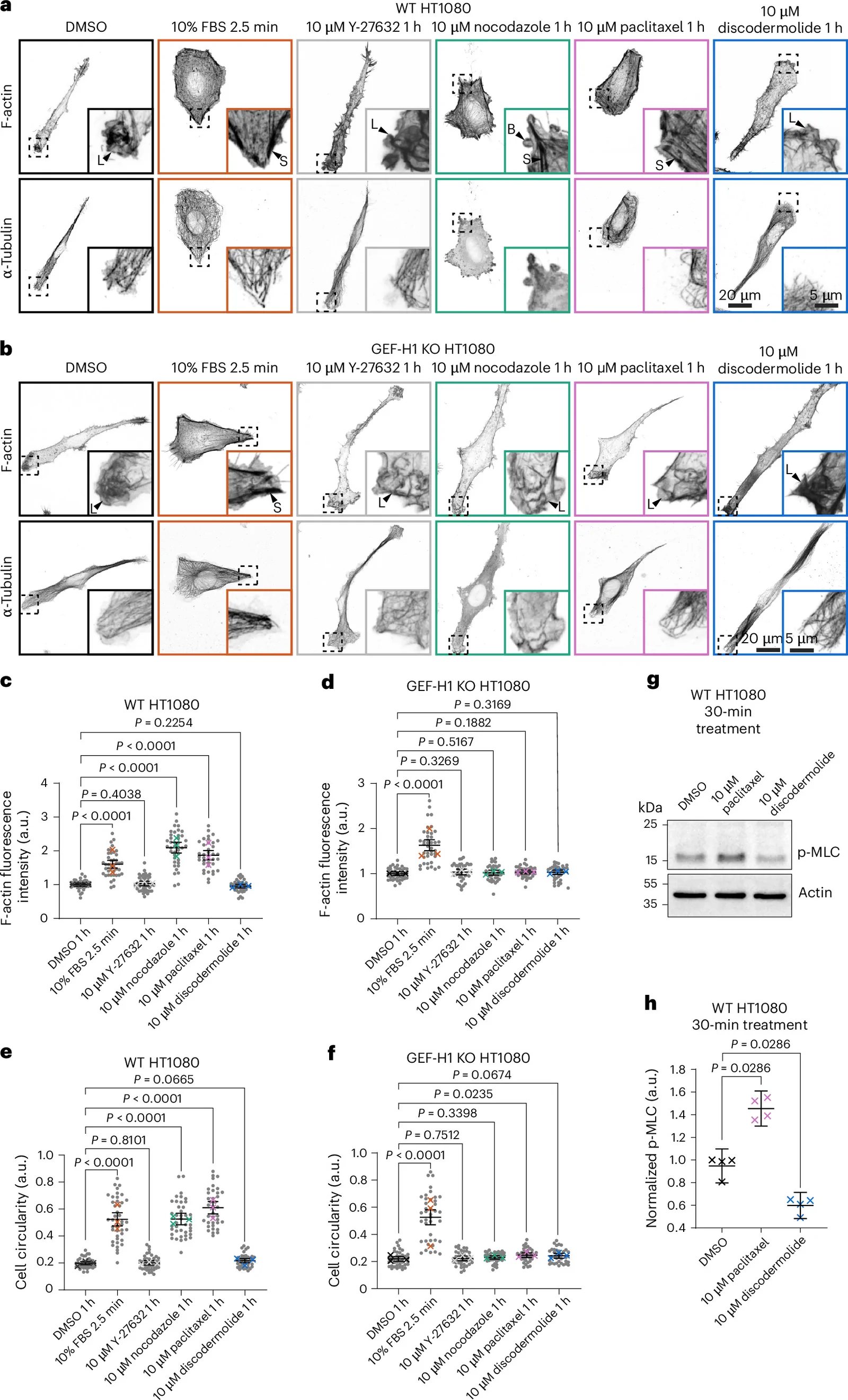
Paclitaxel activates downstream effectors of RhoA in a GEF-H1-dependent manner. **a**–**f**, Serum-starved HT1080 WT (**a**,**c**,**e**) or GEF-H1 KO (**b**,**d**,**f**) cells were treated with paclitaxel, nocodazole, discodermolide, Y-27632 (ROCK inhibitor) as a negative control, or DMSO as a solvent control for 1 h, or FCS as a positive control for 2.5 min, before fixing and staining for F-actin and α-tubulin. **a**,**b**, Representative images, where dashed boxes shows regions highlighted in the zoomed panels; examples of lamellipodia (L), blebs (B) and stress fibres (S) are highlighted with labelled black arrowheads. **c**–**f**, Plots showing F-actin staining intensity (**c**,**d**) or cell circularity (**e**,**f**) normalized to the experimental mean of the DMSO control; individual cells are shown as grey dots, crosses show experimental means, and brackets show 95% confidence intervals based on individual cell values. For **a**,**c**,**e**, DMSO *n* = 39, FBS *n* = 42, Y-27632 *n* = 42, nocodazole *n* = 40, paclitaxel *n* = 39, discodermolide *n* = 39 cells over three independent experiments; for **b**,**d**,**f**, DMSO *n* = 40, FBS *n* = 40, Y-27632 *n* = 37, nocodazole *n* = 38, paclitaxel *n* = 36, discodermolide *n* = 36 cells over three independent experiments. **g**,**h**, Serum-starved HT1080 WT cells were treated with paclitaxel, discodermolide or DMSO as a solvent control for 30 min before collecting cell lysates and blotting for p-MLC and actin as a loading control: representative blot from one experimental replicate (**g**); plot showing p-MLC levels quantified from western blots and normalized to the actin loading control (**h**). Brackets indicate mean ± 95% confidence interval. *n* = 4 experiments. **c**–**f**,**h**, A two-tailed Welch’s *t*-test (**c**–**f**) or two-tailed Mann–Whitney test (**h**) was used to test for statistical significance. Source data

**Extended Data Fig. 2:**
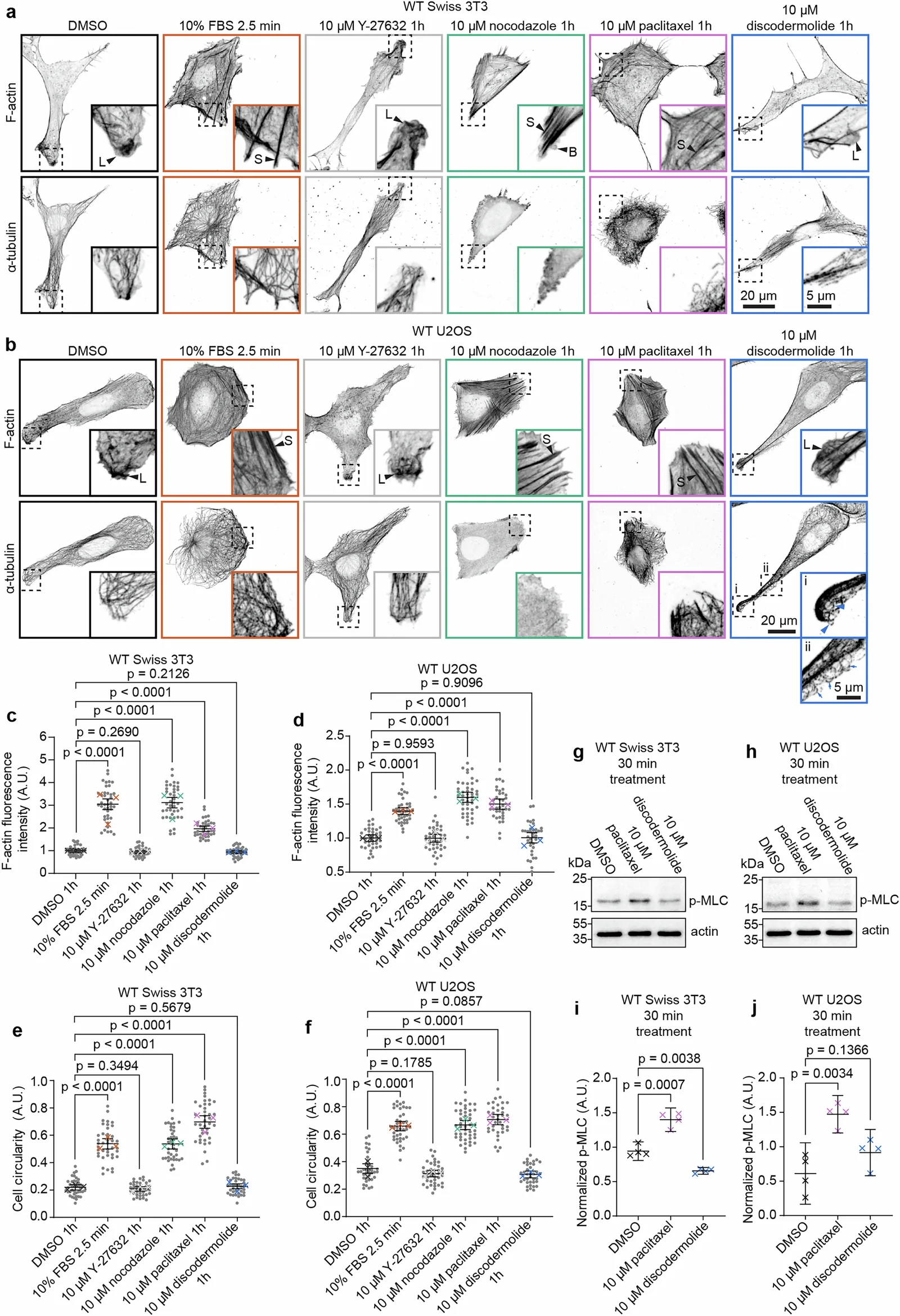
Paclitaxel activates downstream effectors of RhoA in Swiss 3T3 and U2OS cells. (**a**-**f**) Serum starved Swiss 3T3 (**a**, **c**, **e**) or U2OS (**b**, **d**, **f**) cells were treated with paclitaxel, nocodazole, discodermolide, Y-27632 (ROCK inhibitor) as a negative control, or DMSO as a solvent control for 1 hour, or FCS as a positive control for 2.5 min before fixing and staining for F-actin and α-tubulin. (**a**, **b**) Representative images, where dashed box shows region highlighted in zoom panel; examples of lamellipodia (L), blebs (B) and stress fibers (S) are highlighted with labelled arrows. (**b**(i-ii)) blue arrows highlight curved microtubules in discodermolide treated U2OS. (**c**-**f**) Plots show F-actin staining intensity (**c**, **d**) or cell circularity (**e**, **f**) normalized to experimental mean of DMSO control; individual cells are shown as grey dots, crosses show experimental means, brackets show 95% confidence interval based on individual cell values. For (**c**, **e**) DMSO n = 40, FBS n = 41, Y-27632 n = 36, nocodazole n = 40, paclitaxel n = 39, discodermolide n = 38 cells over 3 independent experiments; for (**d**, **f**) DMSO n = 37, FBS n = 41, Y-27632 n = 41, nocodazole n = 45, paclitaxel n = 36, discodermolide n = 36 cells over 3 independent experiments. (**g**-**j**) Serum starved Swiss 3T3 WT or U2OS WT cells were treated with paclitaxel, discodermolide or DMSO as a solvent control for 30 minutes before harvesting cell lysates and blotting for p-MLC and actin as a loading control. (**g,h**) Representative blots from 1 experimental replicate. (**i,j**) Plots show p-MLC levels quantified from Western blots and normalized to the actin loading control. Values represent the experimental mean, and brackets indicate the standard deviation. n = 4 experiments. A two-tailed Welch’s t-test was used to analyse Swiss 3T3 F-actin intensity, Swiss 3T3 circularity, U2OS F-actin intensity and p-MLC quantifications; a two-tailed Mann–Whitney test was used to analyse statistical significance for U2OS cell circularity quantifications. Source data

The main downstream effect of RhoA activation is the stabilization of filamentous actin and increased actomyosin contractility, typically visualized as an increase in stress fibre formation in two-dimensional (2D) cell cultures. Serum-starved HT1080, Swiss 3T3 and U2OS cells were elongated and had only a few stress fibres and lamellipodia at the leading edge (Fig. 2a and Extended Data Fig. 2a,b). Treatment with Y-27632, an inhibitor of Rho-associated protein kinase (ROCK), a kinase acting downstream of RhoA^50–52^, had no strong effect on actin organization or cell shape in these conditions (Fig. 2a,c,e and Extended Data Fig. 2a–f), in agreement with serum starvation reducing the baseline RhoA activity. In contrast, microtubule disassembly with nocodazole induced the formation of stress fibres, blebs and cell rounding (Fig. 2a,c,e and Extended Data Fig. 2a–f). A very similar effect was observed when microtubules were stabilized with paclitaxel (Fig. 2a,c,e). However, stabilization of microtubules by discodermolide did not induce any striking changes in F-actin organization nor cell morphology (Fig. 2a,c,e and Extended Data Fig. 2a–f), even though it did affect microtubule organization: for example, in U2OS cells, it induced tightly curved microtubules (Extended Data Fig. 2b(i,ii), blue arrowheads). We also performed live-cell imaging of actin in HT1080 fibrosarcoma, MDA-MB-231 breast cancer cells and 1205Lu melanoma cells treated with paclitaxel, and observed a fast cell rounding and blebbing response, followed by slight respreading of the cell and formation of prominent stress fibres, whereas no striking difference was observed upon treatment with discodermolide or DMSO (Supplementary Videos 1–6). In GEF-H1 knockout cells, MTA treatments had no pronounced effect on the actin cytoskeleton, and cells retained their elongated shape (Fig. 2b,d,f). In line with the changes in actin organization, paclitaxel also increased the abundance of phosphorylated myosin light chain (p-MLC), a downstream target of RhoA signalling^51^ (Fig. 2g,h and Extended Data Fig. 2g–j). Taken together, these data show that, similar to microtubule-depolymerizing agents, paclitaxel triggers GEF-H1-dependent actin reorganization.

### Paclitaxel induces the release of GEF-H1 from microtubules in cells

**Fig. 3:**
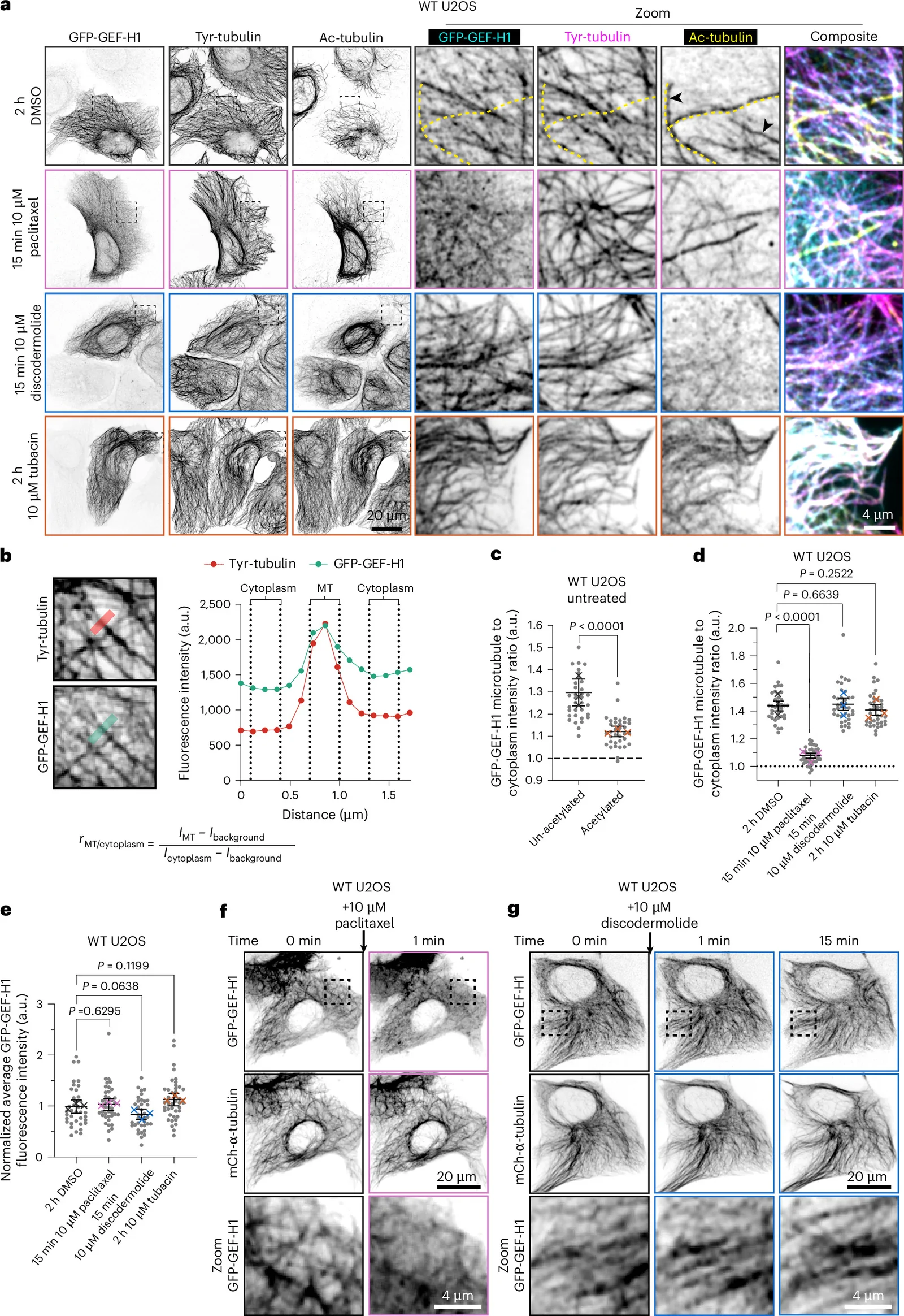
Paclitaxel displaces GEF-H1 from microtubules in cells. **a**–**e**, U2OS cells transfected with GFP-GEF-H1 at low levels were treated for 15 min with paclitaxel or discodermolide, or treated for 2 h with tubacin (inhibitor of tubulin deacetylase HDAC6) or DMSO solvent control before fixing and staining for tyrosinated α-tubulin (tyr-tubulin) or acetylated α-tubulin (ac-tubulin). **a**, Representative images, where dashed boxes indicate regions enlarged in the zoomed panels, and dashed yellow lines highlight acetylated microtubules devoid of GFP-GEF-H1. Experiments were repeated at least three times with similar results. **b**, GFP-GEF-H1 on microtubules was analysed by first drawing line scans perpendicularly across microtubules flanked by cytoplasm and using the maxima of the tyr-tubulin staining to define microtubule and cytoplasmic regions within GFP-GEF-H1 intensity values. The ratio of GFP-GEF-H1 on a microtubule was subsequently calculated by dividing the intensity on the microtubule by the intensity in the cytoplasm. **c**, Plot of GFP-GEF-H1 microtubule-to-cytoplasm ratios of acetylated versus un-acetylated microtubules in the same cell (*n* = 35 cells, over three independent experiments). **d**,**e**, Plot of GFP-GEF-H1 microtubule-to-cytoplasm intensity ratios (**d**) and plot of GFP-GEF-H1 intensity normalized to the experimental mean (DMSO *n* = 37; paclitaxel *n* = 41; discodermolide *n* = 37; tubacin *n* = 39 cells; from three independent experiments; **e**). In **c**–**e**, individual cell means are shown as grey dots, crosses show experimental means, and brackets show 95% confidence intervals based on individual cell values. A two-tailed Wilcoxon matched-pairs test was used to test for statistical significance of GFP-GEF-H1 microtubule binding between acetylated and non-acetylated microtubules, and a two-tailed Welch’s *t*-test was used to compare between DMSO- and drug-treated cells. **f**,**g**, U2OS cells were transfected with mCherry-α-tubulin and GFP-GEF-H1 and imaged live on a spinning disc microscope before (time = 0 min) and after (time = 1 min, 15 min) the addition of paclitaxel (**f**) or discodermolide (**g**) as a microtubule-stabilizing control that does not expand microtubule lattice. The experiment was repeated at least three times with similar results. Source data

**Extended Data Fig. 3:**
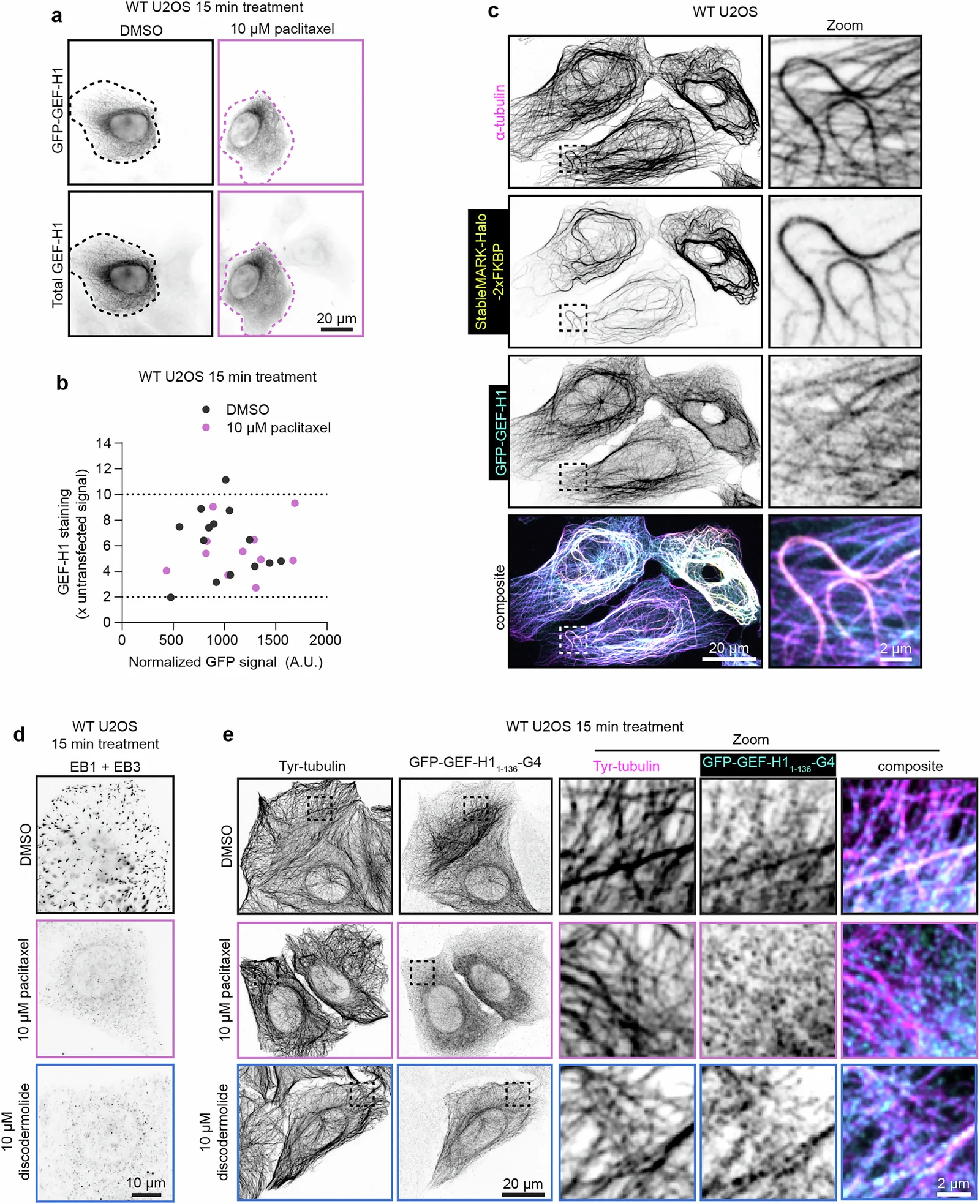
Characterization of cellular effects of MTAs and selectivity of GEF-H1 for expanded microtubules. (**a**, **b**) Analysis of GFP-GEF-H1 overexpression levels used to investigate its microtubule binding and release by paclitaxel. U2OS cells were transiently transfected with GFP-GEF-H1 and treated for 15 minutes with either DMSO or paclitaxel before fixing and staining for GEF-H1 to estimate GEF-H1 overexpression relative to endogenous levels. Intensities were quantified and normalized to untransfected cells; graph in (**b**) shows individual cells analysed. Cells with a similar range of GFP-GEF-H1 expression levels were used to investigate microtubule binding in different conditions. (**c**) U2OS cells transiently co-transfected with GFP-GEF-H1 and StableMARK-Halo-2xFKBP to label the stable microtubule subset, fixed and stained for α-tubulin. (**d**) U2OS cells were treated for 15 minutes with either DMSO, paclitaxel or discodermolide, fixed and stained with antibodies recognizing End Binding proteins EB1 and EB3, which visualize growing microtubule ends. (**e**) U2OS cells transiently transfected with GFP-GEF-H1_1-136_-G4 were treated for 15 minutes with either DMSO, paclitaxel or discodermolide, fixed and stained for tyrosinated tubulin. Source data

As microtubule-destabilizing agents activate GEF-H1 by inducing its unbinding from microtubules^18–21^, we next investigated whether paclitaxel could also release GEF-H1 from microtubules. GEF-H1 immunostaining in cells shows strong variability and ambiguous microtubule co-localization, making reliable quantitative analysis difficult (Extended Data Fig. 1d). To circumvent this problem, we expressed green fluorescent protein (GFP)-tagged full-length GEF-H1 (GFP-GEF-H1) at low and intermediate levels (~2–10 times endogenous levels in U2OS cells; Extended Data Fig. 3a,b) and quantified the GFP signal to ensure comparable expression levels between treatments (Fig. 3a–e). We chose U2OS cells for these experiments as they are more strongly adherent than HT1080 cells and therefore less prone to rounding up in response to MTA treatment. As expected, GFP-GEF-H1 bound along most microtubules in U2OS cells but was barely detected on acetylated microtubules (Fig. 3a, yellow dashed line, and Fig. 3c), in line with previous studies^32,33^. Similarly, GFP-GEF-H1 avoided microtubules labelled with the marker for stable expanded microtubules, StableMARK^39,53^ (Extended Data Fig. 3c). Treatment of cells with 10 µM paclitaxel resulted in dissociation of GEF-H1 from microtubules (Fig. 3a). This effect could be observed and quantified in fixed cells (Fig. 3a,d), and directly visualized by live imaging (Fig. 3f and Supplementary Video 7). GFP-GEF-H1 dissociation was not caused by suppression of microtubule growth, because discodermolide^45,46^, which also efficiently inhibits microtubule elongation, as detected by staining of EB1/EB3 positive microtubule plus ends (Extended Data Fig. 3d), had no effect on GFP-GEF-H1 binding to cellular microtubules (Fig. 3a,d,g and Supplementary Video 8). Similar observations were made in live HT1080 cells; however, paclitaxel-induced cell rounding precluded accurate quantification (Supplementary Videos 9 and 10).

To narrow down the origin of GEF-H1’s sensitivity for taxanes, we looked at the microtubule binding domain of GEF-H1, which consists of a zinc-finger C1 domain flanked by intrinsically disordered regions (amino acids 1–136; Extended Data Fig. 1a). Because full-length GEF-H1 is a coiled-coil dimer^22^, we artificially dimerized the shorter fragment using the dimeric leucine zipper coiled-coil domain of GCN4 (G4). The GFP-GEF-H1_1–136_-G4 construct was also released from microtubules by paclitaxel but not by discodermolide (Extended Data Fig. 3e). This suggests that the first 135 amino acids of GEF-H1 confer its sensitivity to taxane-induced lattice conformations. We also tested shorter fragments of GEF-H1, such as the GCN4-dimerized version of the C1 domain alone (amino acids 28–100), but with this construct we only detected very weak enrichment on microtubules, while versions without the leucine zipper did not show any detectable microtubule binding.

**Extended Data Fig. 4:**
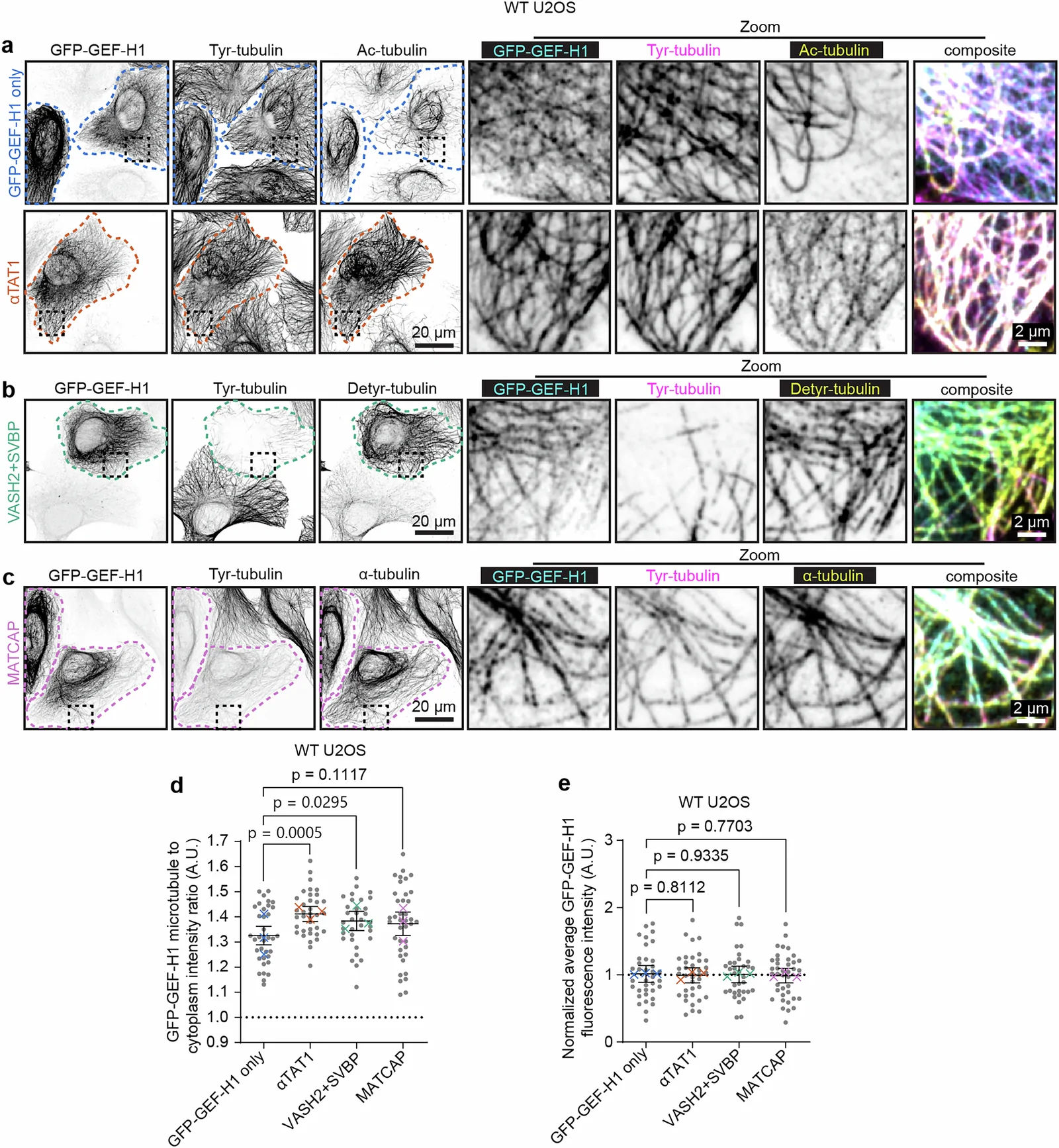
GEF-H1 shows little sensitivity to overexpression of enzymes modifying post-translational microtubule modifications. (**a**-**e**) U2OS stably overexpressing GFP-GEF-H1 at low levels were transiently transfected with αTAT1 (**a**) to induce acetylation, VASH2 + SVBP (**b**) to induce detyrosination or MATCAP (**c**) to induce removal of two C-terminal amino acids of α-tubulin (Δ2). Cells were fixed and stained for tyrosinated tubulin (tyr-tubulin) and either acetylated tubulin (**a**, ac-tubulin), detyrosinated tubulin (**b**, detyr-tubulin) or Δ2-tubulin (**c**). (A-C) Representative confocal images are shown, where coloured stippled lines mark transfected cells and dashed boxes indicate which regions are enlarged in the zoom panels. (**d**) Plot shows GFP-GEF-H1 microtubule to cytoplasm intensity ratio, analysed in untransfected or αTAT1 / VASH2 + SVBP / MATCAP transfected cells as shown in Fig. 3b. (**e**) Plot shows GFP-GEF-H1 intensity normalized to experimental mean of cells for which microtubule binding was analysed. (**d**, **e**) Individual cell means are shown as grey dots, crosses show experimental means and brackets show 95% confidence interval based on individual cell values (GFP-GEF-H1 only n = 36, αTAT1 n = 36, VASH2 + SVBP n = 35, MATCAP n = 39; from 3 experiments) A two-tailed Welch’s t-test was used to test for statistical significance. Source data

GEF-H1 dissociation from microtubules in paclitaxel-treated cells occurred within 1 min of drug addition (Fig. 3f and Supplementary Video 7), whereas discodermolide treatment had no effect (Fig. 3g and Supplementary Video 8). One minute is insufficient to trigger marked microtubule acetylation, as even cells treated with paclitaxel for 15 min did not show striking over-acetylation of microtubules (Fig. 3a). Moreover, treatment of cells with a tubulin deacetylase HDAC6 inhibitor (tubacin^54^) or overexpression of the tubulin acetylase αTAT1^55^, both of which caused acetylation of all cellular microtubules, did not inhibit the binding of GEF-H1 to microtubules (Fig. 3a,d and Extended Data Fig. 4a,d). In fact, we saw a subtle increase in GEF-H1 binding to microtubules upon αTAT1 overexpression (Extended Data Fig. 4d), despite there being no difference in GFP-GEF-H1 fluorescence intensities (Extended Data Fig. 4e). Induction of microtubule detyrosination (removal of the C-terminal tyrosine of α-tubulin), another modification associated with microtubule stability^31^, by overexpressing the detyrosinating enzymes VASH2-SVBP or MATCAP^56–58^, either did not impact the localization of GEF-H1 to microtubules or showed a slight increase in binding (Extended Data Fig. 4b–e). Together, these data indicate that paclitaxel induces the release of GEF-H1 from microtubules through a rapid mechanism that cannot be explained by altered microtubule dynamics or tubulin post-translational modifications.

### Characterization of GEF-H1–microtubule interactions by in vitro reconstitution assays

**Fig. 4:**
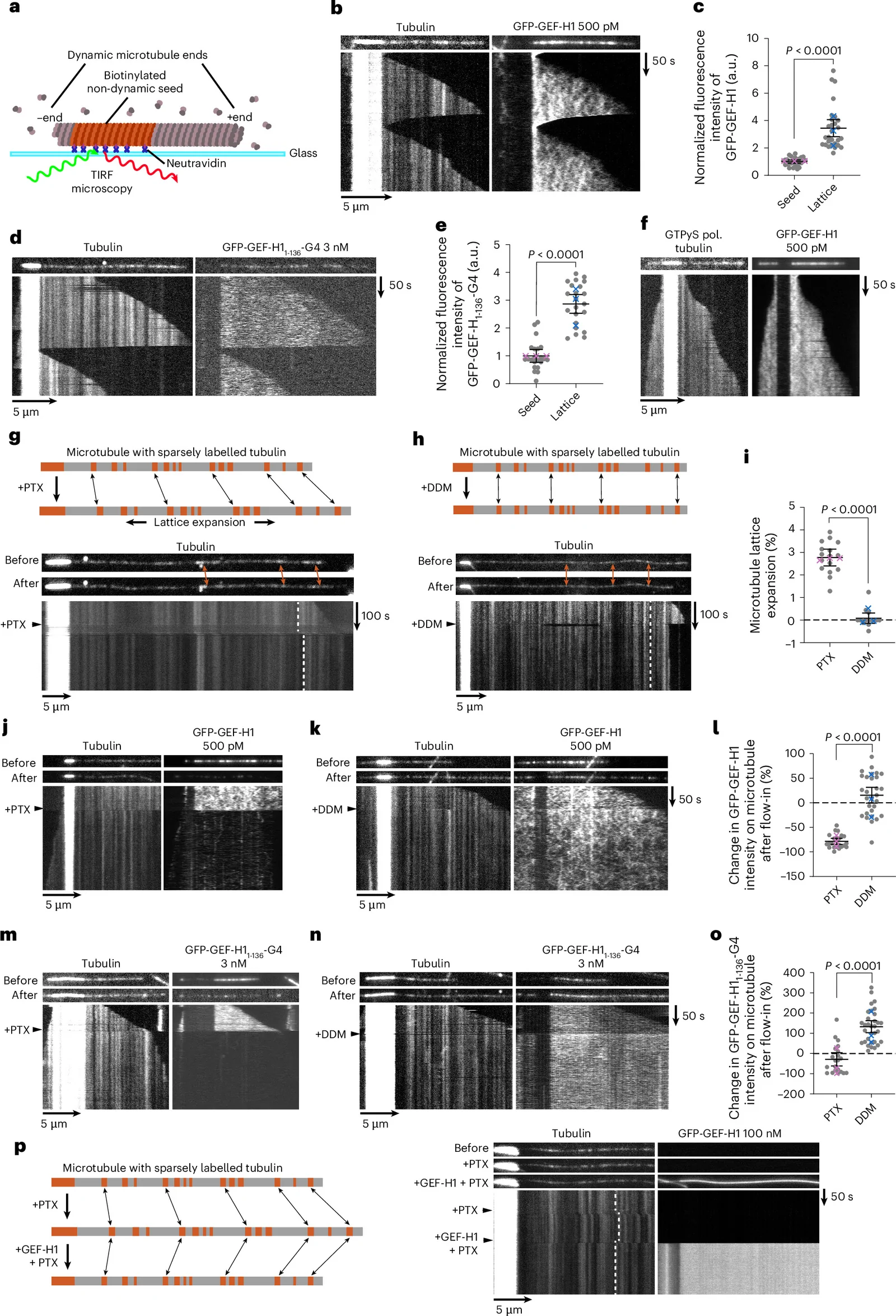
Characterization of GEF-H1–microtubule interactions by in vitro reconstitution assays. **a**, Scheme of the in vitro TIRF assay with dynamic microtubules: the sample is illuminated at an oblique angle (green arrow) so as to only excite fluorescent microtubules and associated proteins attached to the glass (the red arrow shows emissions/reflected light). **b**,**d**, Still images and kymographs of representative microtubules grown with 500 pM GFP-GEF-H1 (**b**) or 3 nM GFP-GEF-H1_1–136_-G4 (**d**). The tubulin channel shows bright GMPCPP seeds (left), and the GFP-GEF-H1 channel (right) shows binding to the dynamic GDP lattice but exclusion from GMPCPP seeds. **c**,**e**, Plots showing mean ± 95% confidence interval of intensity of GFP-GEF-H1 (*n* = 28; from three experiments; **c**) and GFP-GEF-H1_1–136_-G4 (*n* = 22, from three experiments; **e**) quantified at the GMPCPP seed versus GDP lattice and normalized to the mean intensity at the seed. Individual values are depicted as grey dots and experimental means as crosses. **f**, Still images and kymographs of a representative microtubule grown with 500 pM GEF-H1 using GTPγS instead of GTP show that GEF-H1 still avoids GMPCPP seeds but binds the GTPγS lattice. **g**,**h**, Top: schemes of sparsely labelled microtubules used to detect microtubule expansion (or lack thereof) upon drug wash-ins. Bottom: still images of representative microtubules before and after drug wash-ins, illustrating speckle shift upon the addition of paclitaxel (PTX; **g**) but not discodermolide (DDM; **h**). The frame of the wash-in is indicated in the kymographs with an arrowhead; dotted lines highlight speckle shift or lack thereof. **i**, Plot showing mean ± 95% confidence interval of percentage of expansion with PTX or DDM (PTX *n* = 16, DDM *n* = 14; from three experiments). **j**–**o**, Top: still images of representative microtubules before and after PTX or DDM wash-in in assays with microtubules grown in the presence of 500 pM GFP-GEF-H1 (**j**,**k**) or 3 nM GFP-GEF-H1_1–136_-G4 (**m**,**n**); tubulin (left) and GFP (right) channels are shown. Bottom panels: corresponding kymographs, with wash-in frame indicated with an arrowhead. **l**,**o**, Plots showing mean ± 95% confidence interval of percent change in GFP-GEF-H1 (PTX *n* = 22, DDM *n* = 29; from three experiments; **l**) or GFP-GEF-H1_1–135_-G4 (PTX *n* = 21, DDM *n* = 32; from three experiments; **o**) intensity upon PTX or DDM wash-in. **p**, Left: scheme of the experiment used to detect microtubule compaction by GEF-H1. Right: still images before, after PTX, and after 100 nM GEF-H1 addition; arrowheads indicate the frames of wash-ins in the kymographs; dotted lines indicate speckle shift. A two-tailed Mann–Whitney test was used to test for statistical significance. Source data

**Extended Data Fig. 5:**
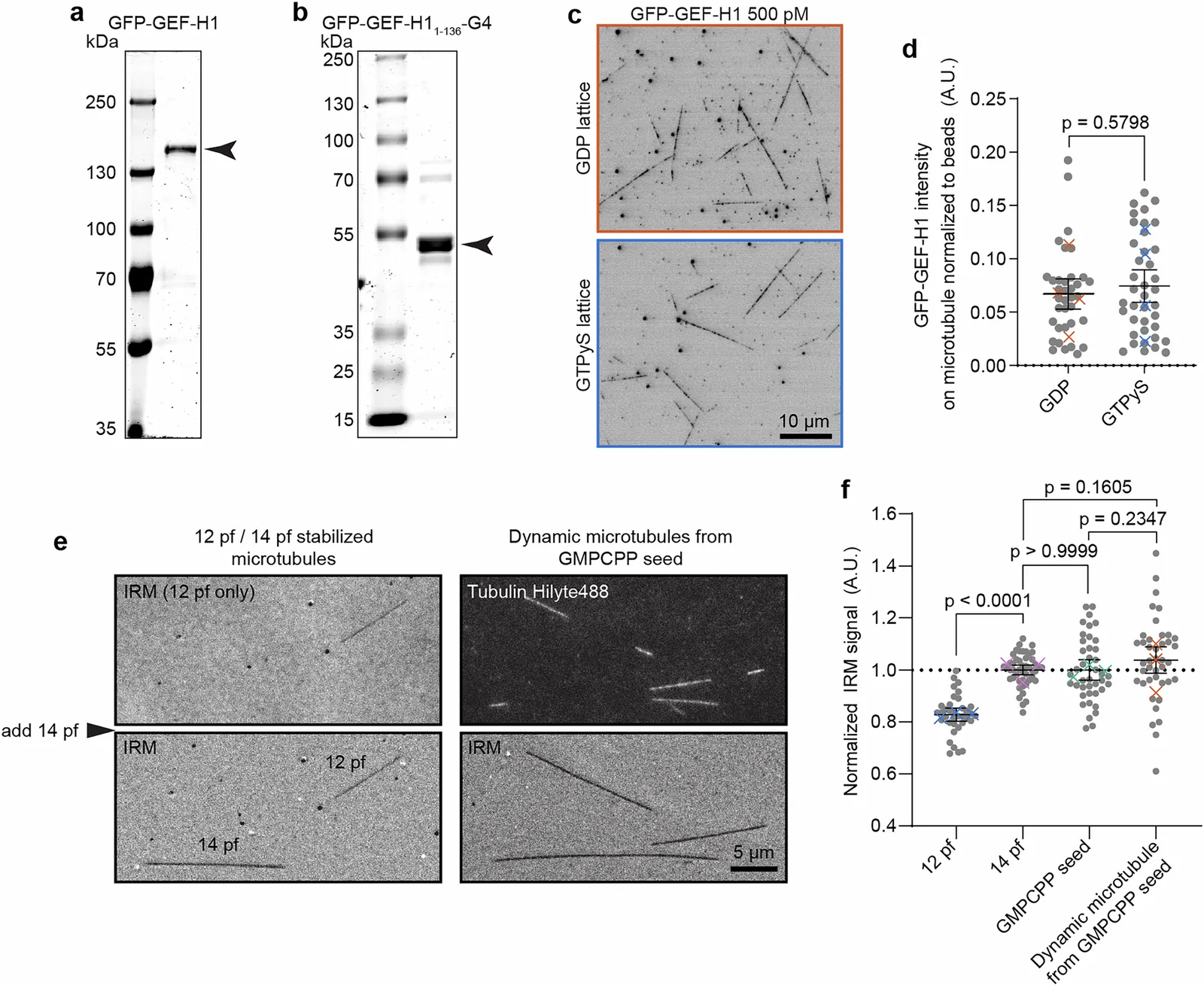
Purified GEF-H1 protein controls and IRM analysis to determine protofilament number. (**a**, **b**) Coomassie gels of purified (**a**) full length GFP-GEF-H1 protein and (**b**) GFP-GEF-H1_1-136_-G4 protein. (**c**, **d**) In vitro assays performed in parallel chambers on the same coverslip, containing fluorescent TetraSpeckTM beads and microtubules grown in the presence of 500 pM GEF-H1 and either 1 mM GTP (resulting in a GDP lattice) or 1 mM GTPyS. (**c**) Representative still images. (**d**) Dot plots showing mean ± 95% confidence interval based on individual values of intensity of GFP-GEF-H1 on GDP vs GTPyS lattice, normalized to the bead intensity per frame (GTPyS, n = 38; GDP, n = 36 from 4 experiments). (**e**, **f**) Interference Reflection Microscopy (IRM) was used to compare microtubules of known protofilament (pf) number and to compare dynamic GDP-microtubules polymerized from 14 pf GMPCPP microtubule seeds. (**f**) IRM signal was normalized to the average signal for GMPCPP 14 pf microtubules; plot shows mean ± 95% confidence interval based on individual microtubule values, with individual microtubules analysed shown as grey dots and experimental averages as crosses (12 pf n = 36, 14 pf n = 47, GMPCPP n = 41, dynamic microtubule n = 41; from 3 experiments). A two-tailed Mann–Whitney test was used to test for statistical significance for the GDP and GTPyS lattice GEF-H1 binding comparison and a Welch’s t-test was used to test the IRM data. Source data

Based on our cellular data, we hypothesized that GEF-H1 binding to microtubules is sensitive to changes in lattice conformation and set out to test this idea using assays with purified proteins. Dynamic microtubules were grown in the presence of fluorescently labelled tubulin from microtubule seeds stabilized with the slowly hydrolysable GTP analogue GMPCPP and observed by total internal reflection fluorescence (TIRF) microscopy (Fig. 4a)^59,60^. GFP-GEF-H1 and GFP-GEF-H1_1–136_-G4 were purified from transiently transfected HEK293T cells (Extended Data Fig. 5a,b). To evaluate protein purity, we used mass spectrometry and detected only minor contamination of the GFP-GEF-H1 preparation with HSP70 chaperones and 14-3-3 proteins (Supplementary Table 1). Both GFP-GEF-H1 and GFP-GEF-H1_1–136_-G4 efficiently accumulated on dynamic microtubule lattices even at very low concentrations (500 pM for the full-length GEF-H1 and 3 nM for GEF-H1_1–136_-G4) but strongly avoided GMPCPP-stabilized seeds (Fig. 4b–e). We used interference reflection microscopy (IRM) to confirm that GMPCPP-stabilized seeds and the dynamic lattices grown from these seeds consist of 14 protofilaments, as published previously^61,62^ (Extended Data Fig. 5e,f). In mammalian cells the majority of microtubules consist of 13 protofilaments^63^, so these data suggest that the GEF-H1–microtubule interaction is not particularly sensitive to protofilament number. Furthermore, avoidance of GMPCPP seeds could not be due to the lack of GTP hydrolysis by β-tubulin within the seed, because 500 pM GFP-GEF-H1-decorated microtubules polymerized in the presence of non-hydrolysable GTP analogue GTPγS, with an efficiency comparable to that of the GDP lattice (Fig. 4f and Extended Data Fig. 5c,d).

**Extended Data Fig. 6:**
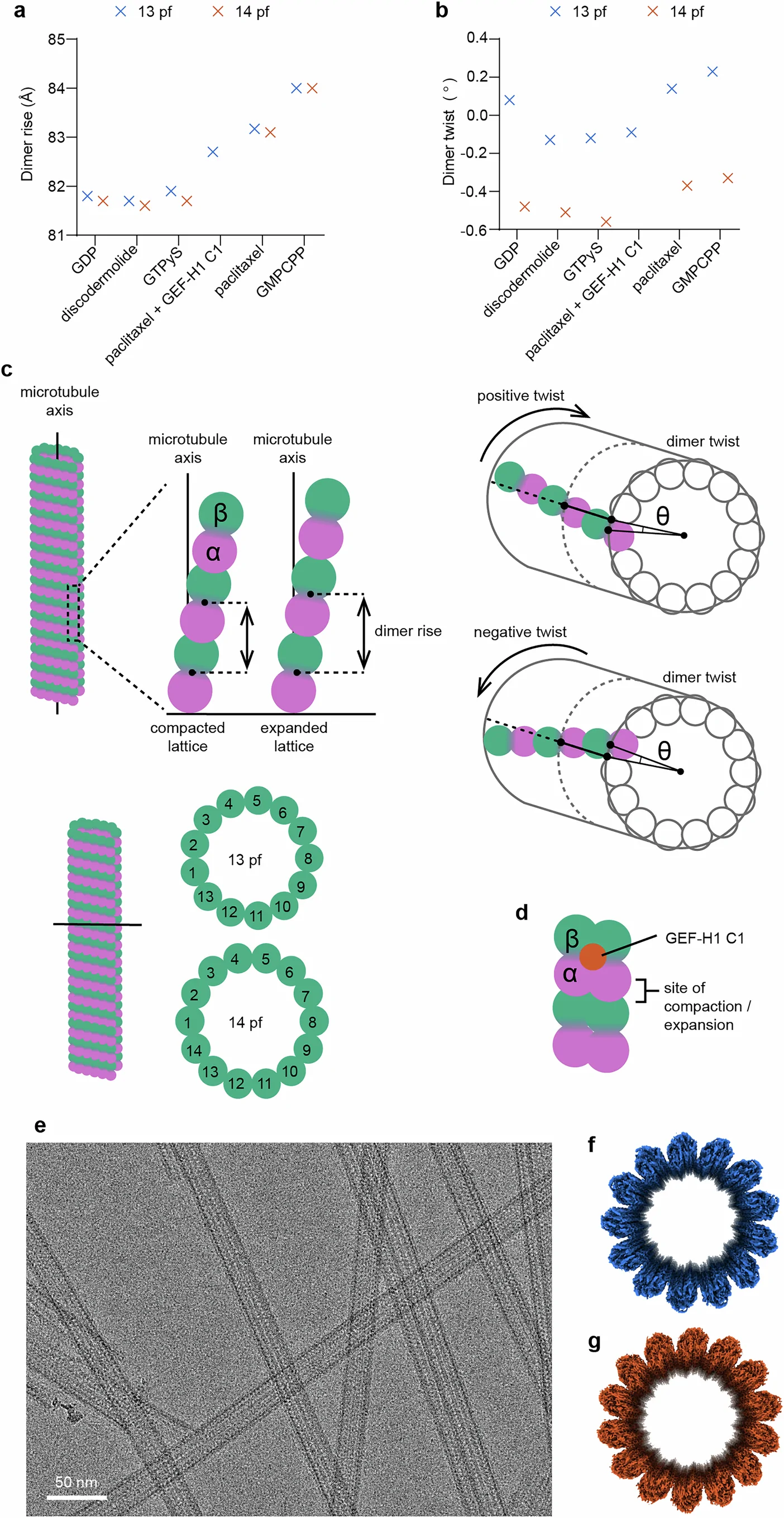
Comparison of microtubule lattice parameters. (**a**, **b**) Plot of dimer rise (**a**) and dimer twist (**b**) for 13 pf and 14 pf microtubules with either dynamic GDP lattice or in the presence of discodermolide (this study), GTPyS, paclitaxel + GEF-H1-C1, paclitaxel alone or GMPCPP. Values for GMPCPP, GTPyS and GDP lattices are adopted from^67^, paclitaxel adopted from^101^, and paclitaxel + GEF-H1-C1 adopted from^22^. (**c**) Illustration of microtubule lattice parameters: dimer rise, which reflects lattice expansion and compaction, protofilament (pf) number and dimer twist. (**d**) Schematic representation of GEF-H1 C1 binding to the microtubule lattice, based on^22^. **(e)** Representative micrograph from 8512 micrographs. Scale bar is 50 nm. **(f)** Cryo-EM map of discodermolide stabilised 13 pf microtubule (3.47 Å). Contour level 0.05. **(g)** Cryo-EM map of discodermolide stabilised 14 pf microtubule (2.73 Å). Contour level 0.07. Source data

To test whether microtubule-stabilizing agents directly affect GEF-H1 binding, we performed wash-in assays with 20 µM paclitaxel or 200 nM discodermolide. Discodermolide has a notably higher affinity to microtubules than paclitaxel and is a known hypernucleator of microtubules in vitro^64,65^. Hence, to avoid spontaneous nucleation interfering with our assays, we used a lower concentration that was still able to prevent depolymerization in vitro. Additionally, to prevent spontaneous microtubule nucleation, the concentration of soluble tubulin during wash-in was reduced from 17 µM to 5 µM. Paclitaxel is known to expand the microtubule lattice in the longitudinal direction^40,66^, an effect that could be readily observed by imaging microtubules sparsely labelled by fluorescent tubulin (Fig. 4g,i). In contrast, discodermolide did not expand the microtubule lattice (Fig. 4h,i). To strengthen this conclusion, we determined the lattice parameters of 13- and 14-protofilament microtubules stabilized with discodermolide using cryo-electron microscopy (cryo-EM) followed by single-particle analysis. We found that discodermolide-stabilized microtubules indeed had a compacted lattice, and had lattice parameters that were highly similar to those of compacted GTPγS-bound lattices (Extended Data Fig. 6a,b; 81.7-Å and 81.6-Å dimer rise, and −0.13° and −0.51° dimer twist, for 13- and 14-protofilament discodermolide-stabilized microtubules, compared to 81.9 Å and 81.7 Å, and −0.12° and −0.56°, for 13- and 14-protofilament microtubules assembled with GTPγS^67^). The binding of GFP-GEF-H1 and GFP-GEF-H1_1–136_-G4 to microtubules was not altered by discodermolide addition, but both proteins very rapidly dissociated after paclitaxel wash-in (Fig. 4j–o). Furthermore, the addition of a 200-fold higher concentration (100 nM) of GFP-GEF-H1 rapidly compacted microtubules that were expanded by paclitaxel addition (Fig. 4p).

Together, these data strongly support the notion that GEF-H1 binding to microtubules is very sensitive to microtubule lattice conformation: it is favoured by compacted, GDP-, GTPγS- or discodermolide-bound lattices, but is incompatible with expanded, GMPCPP- or paclitaxel-bound lattices. However, at sufficiently high concentrations, GEF-H1 can impose its preferred compacted lattice state even on microtubules expanded by paclitaxel.

## Discussion

In this Article we have demonstrated that taxanes can rapidly stimulate RhoA signalling by inducing the release of the RhoA activator GEF-H1 from microtubules, thereby triggering actomyosin contractility and cell rounding. By combining experiments in cells with in vitro reconstitutions using purified components, we revealed that GEF-H1 detachment is driven by taxane-induced conformational changes in the microtubule lattice. Next to microtubule post-translational modifications and tubulin isotypes, lattice conformation is emerging as an important, understudied constituent of the ‘tubulin code’, the structural, biochemical and functional diversification of microtubule subsets in the same cell^68–70^.

Diversity of microtubule conformations includes differences in protofilament number, longitudinal compaction or expansion, and dimer twist^63,66,71,72^ (Extended Data Fig. 6c). Our data indicate that GEF-H1 binds well to 13-protofilament microtubules in cells and 14-protofilament microtubules in vitro. It is the case that 13- and 14-protofilament microtubules have quite different dimer twists (Extended Data Fig. 6b), indicating that GEF-H1 may not be sensitive to this lattice parameter. As GEF-H1 avoids expanded GMPCPP- and paclitaxel-stabilized microtubule lattices but binds well to compacted GDP-, discodermolide- and GTPyS-bound lattices, our data point to GEF-H1 sensitivity to longitudinal expansion/compaction. This conformational change in microtubule lattice mostly affects the interface around the exchangeable nucleotide E-site of β-tubulin (Extended Data Fig. 6c)^40^. However, recent cryo-EM structural analyses have revealed that the C1 domain of GEF-H1 (amino acids 28–100) binds to 13-protofilament, paclitaxel-stabilized microtubules at a site between two αβ-tubulin heterodimers located in adjacent protofilaments^22^. This binding site is located close to the non-exchangeable nucleotide N-sites on the two adjacent α-tubulins and is distant from the E-sites on the corresponding β-tubulins (Extended Data Fig. 6d). Interestingly, the structure of the microtubule–GEF-H1-C1 complex revealed a partially compacted lattice and a dimer twist similar to what is seen in GTPγS- and discodermolide-stabilized microtubules (Extended Data Fig. 6a,b). Although the specific intradimer/interprotofilament binding mode of the GEF-H1 C1 domain may account for its effect on dimer twist, it is unclear how it promotes lattice compaction in paclitaxel-expanded microtubules. One possibility is that binding of the GEF-H1 C1 domain triggers an allosteric effect at its binding site, which is then transmitted to the β-tubulin E-site. Further studies using longer GEF-H1 fragments with stronger microtubule-binding affinity and microtubules that bind GEF-H1 well (for example, GTPγS-microtubules) could help clarify the structural basis for the preference of GEF-H1 for certain lattice conformations.

Our cellular, in vitro and cryo-EM work has established that discodermolide, a compound that stabilizes microtubules and perturbs cell division similar to taxanes^65^, is a very useful control for taxanes. Cryo-EM data showed that, unlike taxanes, discodermolide stabilizes compacted microtubule lattices; this is in line with recent analysis based on fibre diffraction^41^. Similar to paclitaxel, discodermolide inhibited microtubule growth in cells but did not trigger GEF-H1 detachment from microtubules. This control will be valuable for distinguishing the impact of changes in lattice conformation from effects on microtubule growth and mitotic arrest in future investigations into the mechanism of action of MTAs. Additionally, discodermolide can be utilized to distinguish the sensitivity of microtubule-binding proteins to lattice conformation versus microtubule dynamics. Our study hence provides a useful framework for elucidating the sensitivity of microtubule-binding proteins and cellular processes to the conformational component of the tubulin code.

An important feature of microtubule lattice plasticity, in contrast to biochemical modifications, is that proteins serving as ‘conformational code readers’ can become ‘code writers’ at a high concentration^73^. This explains why the sensitivity of GEF-H1 to taxanes has been overlooked previously^19^: when strongly overexpressed, GEF-H1 can induce its preferred lattice conformation, which cannot be altered by paclitaxel. However, endogenous GEF-H1 is a signalling molecule present at levels that are sufficient to detect but not to alter microtubule lattice compaction. In cells, GEF-H1 avoids acetylated, stable microtubules^32,33^, probably because their lattices are expanded^39,53^ and are thus more similar in conformation to paclitaxel- and GMPCPP-stabilized microtubules. Instead, GEF-H1 prefers compacted microtubules, a lattice conformation characteristic of dynamic, short-lived microtubules. This conformational preference is optimal for targeting GEF-H1 to cell protrusions where microtubules are actively remodelled, and prevents its sequestration on stable perinuclear microtubules. In this way, GEF-H1 can control RhoA activity in response to local changes in the density of dynamic microtubules near the cell margin, for example, to promote cell invasion into a dense matrix or trigger cell retraction and help cells with long and elaborate protrusions, such as migrating dendritic cells, to maintain their coherence^21,24–26^. Furthermore, local stabilization and expansion of microtubules in the vicinity of focal adhesions^32,74^ can potentially trigger GEF-H1 release and RhoA-dependent focal adhesion disassembly during cell movement. Taxane treatment overrides these native pathways of localized RhoA activation and causes overall RhoA-dependent cell blebbing and rounding, explaining why it has a strong inhibitory effect on cell motility.

Activation of RhoA by taxanes was probably overlooked previously because, in cycling cultured cells, this effect is quickly overshadowed by mitotic arrest, especially at low concentrations, when taxane accumulation in cells is slow. The ability of taxanes to activate RhoA can be relevant for understanding their efficacy in cancer therapy, because RhoA can promote anti-tumour immune responses^19,75,76^. RhoA signalling might also potentially contribute to cell death, because it can promote apoptosis in low-adhesion environments by increasing cell contractility, explaining why ROCK inhibitors are a standard component of media for stem cells and organoid isolation and maintenance in culture^77–81^. On the other hand, misregulation of RhoA might contribute to the side effects of taxanes, such as neuropathy^82,83^. On a broader scale, taxane-induced changes of microtubule lattice conformation may alter binding of additional microtubule-associated proteins and thus perturb other signalling pathways besides RhoA—a concept deserving future study.

## Methods

### Cell culture

HT1080 (a gift from J. Klumperman, UMC Utrecht), U2OS (ATCC; HTB-96), Swiss 3T3 (ATCC; CCL-92), MDA-MB-231 (mCh-Lifeact; described in ref. ^84^), 1205Lu (Lifeact-eGFP; described in refs. ^85,86^) and HEK293T cells (ATCC; CRL-3216) were cultured in Dulbecco’s modified Eagle medium (DMEM; Capricorn Scientific or Gibco) supplemented with 10% fetal bovine serum (FBS; Corning) and 100 U ml^−1^ penicillin and 100 µg ml^−1^ streptomycin (1% Pen Strep; Sigma). Cells were kept at 37 °C with 5% CO_2_ and routinely tested for mycoplasma using a commercial assay (Mycoaltert assay, LT07-518; Lonza). No commonly misidentified cell lines were used, and cells were not authenticated.

### Serum starvation

For assays directly or indirectly assessing RhoA activation, cells were first serum-starved to lower the baseline RhoA activity as follows: cells were plated at 10–15% confluency in normal culture medium containing 10% FBS, transferred to medium containing 0.5% FBS 24 h after plating and changed to FBS free medium a further 24 h after that, then incubated overnight (16–18 h) before commencing the experiment. Swiss 3T3 cells were directly transferred from full medium to serum-free medium and starved for 24 h.

### DNA constructs

GFP-GEF-H1 was a gift from S. Etienne-Manneville (Institut Pasteur, Paris, France). For protein purification, a strepII-tagged GFP-GEF-H1 was generated by Gibson assembly using GFP-GEF-H1 as a template to clone into a SII-GFP expression vector. For GFP-GEF-H1_28–100_, GFP-GEF-H1_28–100_-G4, GFP-GEF-H1_1–136_ and GFP-GEF-H1_1–136_-G4, a synthetic gene encoding amino acids 1–136 of GEF-H1 (UniProt Q92974-1), comprising the C1 domain and its adjacent disordered regions, was purchased from Genewiz-Azenta. This was used as a template to amplify GEF-H1_28–100_ and GEF-H1_1–136_. Gibson assembly was used to clone both GEF-H1_1–136_ and GEF-H1_28–100_ into either an SII-GFP expression vector or a modified SII-GFP vector containing a GCN4 coiled-coil domain to promote dimerization. F-tractin-mStayGold was a gift from A. Miyawaki (Addgene 212019)^87,88^. mCh-α-tubulin consisted of a human α-tubulin fused with mCherry^89^. pLenti-RhoA2G^42^ was a gift from O. Pertz (Addgene 40179), FLAG-aTAT1^32^ was a gift from S. Etienne-Manneville (Institut Pasteur, Paris, France), VASH2-FLAG and SVBP-FLAG were a gift from M. Martin (University of Brussels, Belgium), and HA-MATCAP^58^ was a kind gift from T. Brummelkamp (Netherlands Cancer Institute, Amsterdam, the Netherlands). psPAX2 (Addgene 12260) and pMD2.G (Addgene 12259) lentiviral packaging constructs were a gift from D. Trono (EPFL, Lausanne, Switzerland). pSpCas9(BB)–2A-Puro (PX459) V2.0^90^ (Addgene 62988) was a gift from F. Zhang (Broad Institute of MIT and Harvard, Cambridge, USA).

### Cell transfection

Cells were plated at 15% confluency the day before transfection. A ratio of 1 µg plasmid DNA to 3 µl Fugene6 (Promega) transfection reagent was used, and the transfection mix was prepared in optiMEM (Gibco) according to the manufacturer’s instructions. Cells were used for experiments 24 h after transfection.

### Western blotting

Cell lysates were collected on ice using RIPA buffer (20 mM Tris pH 7.5, 100 mM NaCl, 1% NP-40, 0.1% sodium dodecyl sulfate (SDS), 0.2% sodium deoxycholate, 10 mM ethylenediaminetetraacetic acid (EDTA)) containing freshly added broad spectrum protease inhibitors (cOmplete protease inhibitor cocktail, Roche, 11873580001) and phosphatase inhibitors (PhosSTOP, Roche, 4906837001), mixed with loading dye (50 mM Tris pH 6.5, 2% SDS, 1.5 mM bromophenol blue, 8% glycerol) and 5% β-mercaptoethanol, then boiled for 10 min. Lysates were run on 5–16% acrylamide gels and transferred, to polyvinylidene difluoride (PVDF) membrane (0.45-µm pore size, Merck Millipore, IPVH00010) or nitrocellulose membrane (0.2-µm pore size, Amersham, 10600006) in the case of p-MLC, using wet transfer at 37 V and 4 °C overnight. Membranes were blocked using 5% bovine serum albumin (BSA) in Tris-buffered saline with 0.1% Tween 20 (TBST) for 2–8 h, then incubated in primary antibody diluted in blocking buffer, rabbit anti-p-MLC (Cell Signaling, 3671S, dilution 1:250) or rabbit anti-GEF-H1 (Abcam, ab155785, dilution 1:1,000) overnight or mouse anti-actin (Sigma, MAB1501, dilution 1:4,000) for 1 h. Blots were washed three times for 5 min each with TBST after primary antibody incubation, then incubated with goat anti-mouse horseradish peroxidase (HRP)-conjugated secondary (Agilent, P044701-2, 1:2,500 dilution) or swine anti-rabbit HRP-conjugated secondary (Agilent, P039901-2, 1:2,500 dilution). Blots were again washed three times for 5 min each with TBST, then developed using ECL western blotting substrate (Promega, W1015) and imaged using an Amersham ImageQuant 800 set-up, taking care not to saturate the signal. Actin was used as a loading control. Quantifications of p-MLC blots were performed in Image J. The p-MLC signal was first normalized to the actin loading control, and then to the experiment average.

### GEF-H1 knockout cell line generation

Anti-GEF-H1 targeting sequence 5′-ATGGGCACCTCTTCACCACC-3′ was cloned into the pSpCas9(BB)–2A-Puro (PX459) V2.0 plasmid using the BbsI restriction site. This GEF-H1 targeting CAS9 plasmid was then transfected into HT1080 cells as described above. At 24 h after transfection, 3 μg ml^−1^ puromycin (InvivoGen, ant-pr-1) was added to the medium to select for transfected cells for 24 h. From the surviving cell pool, single cells were plated in a 96-well plate and expanded into clonal cell lines. These were screened for GEF-H1 knockout (KO) by immunofluorescence, western blotting and by performing targeted sequencing of genomic DNA. Genomic DNA was extracted using the GeneJET Genomic DNA Purification Kit (Thermo Scientific, K0722), then amplified using the following primers—forward 5′-GCTCTGCTTTAGGAACTGGTGT-3′ and reverse 5′-GAGAGAAGAGTGACCCTCATGG-3′—then repurified and sent for sequencing using the reverse primer.

### RhoA2G stable cell line generation

Lentivirus was used to generate stable cell lines expressing the RhoA2G FRET sensor. Lentivirus was produced in HEK293T cells. For this purpose, cells were plated in a 10-cm dish to be 90% confluent on the day of transfection. Cells were transfected with 15 μg of pLenti-RhoA2G, 10 μg of psPAX2 and 5 μg of pMD2.G using 90 μl of MaxPEI (1 mg ml^−1^ polyethylenimine, Polysciences) in optiMEM (1.2 ml total volume). Transfection mix was vortexed briefly and incubated at room temperature (r.t.) for 10 min before adding to the cells. Cell medium was refreshed the next day using 7 ml of medium, and the conditioned medium was discarded. Two days after transfection, conditioned medium was collected and stored at 4 °C, and 7 ml of fresh medium was applied. Three days afterwards, transfection medium was harvested and combined with medium harvested the previous day, spun for 5 min at 3,000*g* to pellet any floating cells, then supernatant was filtered through a 0.045-μm filter to remove debris. The filtrate was applied to an Amicon Ultra-15 filter column (Merck, UFC903029) and centrifuged for 30 min at 3,000*g* to purify virus. The remaining supernatant with virus was aliquoted and stored at −80 °C until use for cell transduction.

HT1080 WT or GEF-H1 KO cells were plated in a 12-well plate such that they would be 40–50% confluent on the day of transduction. Medium was refreshed with medium containing 6 μg ml^−1^ polybrene (Merck, TRI-1003-G) before applying virus. Lentivirus was thawed on ice and 5 μl applied per well. At 24 h after transduction, the medium was refreshed with medium containing 3 μg ml^−1^ puromycin (InvivoGen, anti-pr-1) to select for transduced cells. After 24 h of antibiotic selection, the cells were returned to normal cell culture medium, expanded, and frozen down for future use.

### RhoA2G FRET experiments

RhoA2G HT1080 WT or GEF-H1 KO cells were plated on 25-mm coverslips and serum-starved as described above. Just before the start of the experiment, coverslips were mounted inside prewarmed imaging rings (Thermo Scientific, A7816) with 1 ml of serum-free medium. FRET experiments were conducted on a Zeiss LSM880 Airyscan microscope fitted with an S1 (Pecon) temperature control and CO_2_ system set to 37 °C and 5% CO_2_, and a ×100/1.46 Alpha Plan-APO oil immersion objective. ZEN black v2.3 software was used to operate the microscope, a 445-nm laser was used to excite the monomeric teal fluorescent protein (mTFP), the emission for mTFP was collected between 472 and 500 nm, and the emission for mVenus was collected between 565 and 654 nm. These settings had been optimized previously to minimize crosstalk using cells containing only mTFP or mVenus as controls. One frame was acquired every 2 min, and five frames were acquired immediately before drug/dimethyl sulfoxide (DMSO) or serum addition.

### RhoA G-LISA GTPase activation assay

A RhoA G-LISA GTPase activation assay kit purchased from Cytoskeleton (BK121) was used according to the manufacturer’s instructions to quantify active RhoA in cells. HT1080 cells were plated at 10% confluency in 10-cm dishes and serum-starved as described above. Cells were treated with either DMSO, paclitaxel or docetaxel, for the concentrations and times indicated, before the cells were washed on ice in a refrigerated room (4 °C) with ice-cold phosphate-buffered saline (PBS; taking care to remove all the PBS by aspirating at an angle on ice for 1 min) and lysed with ice-cold lysis buffer from the kit. Cell lysates were homogenized using a syringe with a 27-G needle and pre-cleared by centrifuging in a centrifuge pre-cooled to 4 °C for 2 min at 10,000*g*. The supernatant was aliquoted on ice into ice-cold Eppendorf tubes, snap-frozen, and stored at −80 °C until the start of the G-LISA essay. One of the aliquots was used for quantifying the protein concentration using a BCA assay. A final concentration of 250 ng protein µl^−1^ was used in the assay after dilution with the binding buffer. An enzyme-linked immunosorbent assay (ELISA) was conducted carefully following the manufacturer’s instructions. A FLUOstar OPTIMA plate reader was used to quantify luminescence. Each experiment contained duplicates, the average of the background (lysis buffer only) was subtracted from the average of the sample, and the result was normalized to the mean of the experiment.

### Immunofluorescence cell staining

To stain F-actin, 4% paraformaldehyde (PFA) fixation was used, and to stain microtubule binding proteins such as GEF-H1 or EB1 + EB3, methanol fixation was used. For PFA fixation, cells were fixed at r.t. for 15 min using 4% PFA in MRB80 buffer (80 mM K-Pipes, 1 mM EGTA, 4 mM MgCl_2_; pH 6.80 with KOH) prewarmed to 37 °C. For methanol fixation, cells were fixed on ice for 15 min using −20 °C methanol. After fixation, cells were washed three times with PBS. In the case of 4% PFA fixation, cells were permeabilized with 0.2% Triton-X in PBS for 2.5 min. The cells were then blocked in blocking buffer (2% BSA in PBS with 0.05% Tween 20) for 1 h. Primary antibodies were diluted in blocking buffer and incubated on cells for 1 h; the antibodies used were rat anti-tyrosinated-α-tubulin (Thermo, MA1-80017; dilution 1:200), mouse anti-α-tubulin (Sigma, T6199; dilution 1:200), mouse anti-acetylated-tubulin (Sigma, T7451; dilution 1:200), rabbit anti-de-tyrosinated-tubulin (Abcam, ab48389, dilution 1:200), rat EB1 + EB3 (Absea, 15H11; dilution 1:10), rabbit anti-GEF-H1 (Abcam, ab155785; dilution 1:150) and mouse anti GFP (Sigma, 11814460001; dilution 1:200). Coverslips were washed by dunking in two different containers of PBS with 0.05% Tween 20, then secondary antibodies or phalloidin diluted in blocking buffer were incubated on cells for 1 h; the secondary antibodies or phalloidin used were donkey anti-rat-Alexafluor-488 (Thermo, A21208; dilution 1:200), goat anti-rat-Alexafluor-594 (Thermo, A11007; dilution 1:200), AlexaFluor-594 conjugated phalloidin (Thermo, A12381; dilution 1:200), goat anti-mouse-AlexaFluor-488 (Thermo, A11029; dilution 1:200) goat anti-mouse-DyLight-405 (Jackson ImmunoResearch, 115-475-166; dilution 1:150), goat anti-rabbit-Alexafluor-405-plus (Thermo A48253; dilution 1:200) and goat anti-rabbit-AlexaFluor-488-plus (Thermo, A32731; dilution 1:200). The samples were mounted with Mowiol mounting medium (10% Mowiol, Sigma, 81381; 30% glycerol, 60% 0.2 M Tris, pH 8.5) with 2.5% DABCO (Sigma, D2522) and allowed to set overnight.

Images of fixed cells for analysis were acquired on a Nikon Eclipse 80i widefield microscope using a Photometrics CoolSNAP MYO CCD camera and a Plan Apo VC ×100/1.40 oil objective. A CoolLED pE-300 system was used to excite the sample. A Chroma ET-DAPI (49000) filter was used to image AlexaFluor-405 and DyLight-405, a Chroma ET-GFP (49002) filter was used to image AlexaFluor-488 or GFP, and a Chroma ET-mCherry (49008) filter was used to image AlexaFluor-594. Nikon NIS Br software was used to operate the microscope.

The images of cells in Figs. 2 and 3a and Extended Figs. 3 and 5 were acquired on a Leica TCS SP8 confocal microscope using a ×100 (oil) HC PL APO ×100/1.40 objective. The microscope was equipped with a 405-nm direct-modulated (DMOD) flexible laser and a white laser (programmable between 470 and 670 nm), two photomultiplier tube detectors and three hybrid detectors. LAS X software was used to control the microscope.

### Live-cell imaging with drug addition

Cells were seeded on 25-mm glass coverslips or glass-bottomed dishes (Cellvis) and, if not already stably expressing fluorescent markers, transfected with GFP-GEF-H1 and mCh-α-tubulin or F-tractin-mStayGold as described above, and serum-starved if applicable. At 48 h after transfection, the coverslips were mounted in prewarmed imaging rings (Thermo, A7816) with 1 ml of medium, and cells plated on glass-bottomed dishes were used directly. Cells were imaged either on a custom Yokogawa spinning disc or an Andor Dragonfly spinning disc.

The Yokogawa spinning disc microscope had an Eclipse Ti-E body (Nikon) with perfect focus, a MS-2000-XYZ stage with Piexo Top Plate (ASI), a CSU-X1-A1 spinning disc unit (Yokogawa) and STXG-PLAMX-SETZ21L temperature and CO_2_ control (TokaiHit). An S Fluor ×100 oil objective (Nikon) was used to acquire images. Voltran Stradus 488-nm (Vortran) and Coherent OBIS 561-nm (Coherent) lasers were used to excite the sample, and ET525/50m (GFP) and ET630/75m (mCherry) filters were used to filter the signal. A prime BSI sCMOS camera (Teledyne Photometrics) was used to take images, and MetaMorph 7.10 (Molecular Devices) software was used to control the microscope.

The Andor Dragonfly spinning disc microscope had an Eclipse Ti-E (Nikon) body with perfect focus, dual Andor Zyla 4.2 sCMOS cameras, and was fitted with a ×60 Plan apo oil objective. A 488-nm laser line was used to excite GFP or mStayGold, with a 525/50 filter to filter the signal, and a 561-nm laser was used to excite mCherry, with a 600/50 filter to filter the signal. The microscope was operated with Fusion software (v2.3.0.36).

In both cases, microscope incubation chambers were preheated to 37 °C with 5% CO_2_. One image was taken every 30 s (GFP-GEF-H1 imaging) or 1 min (F-actin imaging); the first 10 min acquired served as a baseline, after which either 10 µM paclitaxel (PTX) or discodermolide (DDM) was applied to cells and cells were imaged for another 10–15 min or 1 h.

### Analysis of cell images

#### FRET analysis

The fluorescence intensities of the mTFP/donor and mVenus/acceptor channels for FRET experiments were quantified in Image J. Cell outlines were created, and the mean pixel intensity (mean grey value) quantified for both channels at all time points, alongside the respective background measurements (taken for an area without cells). The FRET ratio *r*_FRET_ was subsequently calculated using the following equation, where *I*_donor_ and *I*_acceptor_ are the intensities of the donor and acceptor channels, respectively:

$${r}_{\mathrm{FRET}}=\,\frac{{I}_{\mathrm{acceptor}\mathrm{cell}}-{I}_{\mathrm{acceptor}\mathrm{background}}}{\left({I}_{\mathrm{acceptor}\mathrm{cell}}-{I}_{\mathrm{acceptor}\mathrm{background}}\right)+\left({I}_{\mathrm{donor}\mathrm{cell}}-{I}_{\mathrm{donor}\mathrm{background}}\right)}$$

##### Quantification of fluorescence intensity

The GFP-GEF-H1 fluorescence intensity or Phalloidin staining intensity was measured in Image J by outlining the cell and quantifying the mean pixel intensity (mean grey value) and subtracting the mean pixel intensity of an area without cells (background). Final values were normalized to the experiment’s mean.

#### Quantification of GFP-GEF-H1 binding to microtubules

To ensure comparable GFP-GEF-H1 expression levels between treatments, upper and lower thresholds for the GFP-GEF-H1 signal were set for each experiment, and cells with GFP-GEF-H1 signals above and below the threshold were excluded. The GFP-GEF-H1 microtubule-to-cytoplasmic ratio *r*_MT/cytoplasm_ was quantified in Image J. First, the channels were aligned using the template-matching plugin, then the mean GFP-GEF-H1 fluorescence was determined for all cells as described above, and only cells with comparable expression levels across different treatment groups were used for subsequent analysis. For each cell, ten line scans, three pixels wide, were drawn perpendicular to the microtubule in regions where the cytoplasm was devoid of microtubules on either side of the microtubule. The GFP-GEF-H1 intensity on the microtubule, *I*_MT_, was calculated by taking the mean pixel intensity at the location with the maximum value of the tubulin staining plus the value directly on either side of that maximum; the GFP-GEF-H1 intensity in the cytoplasm, *I*_cytoplasm_, was defined as the values 4–6 pixels before and after the maximum of the tubulin staining. These distances were determined by looking at line scans across microtubules, as shown in Fig. 3b. The mean background pixel intensity, *I*_background_, was measured in a region devoid of cells. The background was subtracted from the GFP-GEF-H1 intensity on the microtubule and in the cytoplasm, then the ratio of GFP-GEF-H1 on the microtubule and GFP-GEF-H1 in the cytoplasm was taken as follows:

$${r}_{\mathrm{MT}/\mathrm{cytoplasm}}=\frac{{I}_{\mathrm{MT}}-{I}_{\mathrm{background}}}{{I}_{\mathrm{cytoplasm}}-{I}_{\mathrm{background}}}$$

The mean cell microtubule-to-cytoplasm ratio over the ten line scans was plotted.

#### Protein purification

The proteins were purified from HEK293T cells using Strep(II)-streptactin affinity purification as described previously^60^. The cells were transfected with a 1:3 wt/wt mix of DNA construct and PEI (Polysciences) and harvested 48 h after transfection in lysis buffer (50 mM HEPES, 1 mM MgCl_2_, 1 mM dithiothreitol (DTT), 300 mM NaCl and 0.5% Triton X-100; pH 7.4) containing protease inhibitors (Roche). They were kept on ice for 15 min and centrifuged to clear the debris. The lysate was incubated with StrepTactin beads (IBA Lifesciences) for 45 min. The beads were washed five times with lysis buffer (high salt washes) and three times with lysis buffer but with 150 mM NaCl instead of 300 mM (low salt washes). The protein was then eluted in elution buffer (50 mM HEPES, 150 mM NaCl, 1 mM MgCl_2_, 1 mM EGTA, 1 mM DTT, 0.05% Triton X-100 and 2.5 mM d-desthiobiotin (Sigma-Aldrich), pH 7.4). All purified proteins were snap-frozen and stored at −80 °C.

#### Mass spectrometry

##### Protein digestion

For liquid chromatography–tandem mass spectrometry (LC–MS/MS) analysis, 2 mg of GFP-GEF-H1 purified protein was used. The volume was adjusted to 20 µl using elution buffer (50 mM HEPES pH 7.4, 150 mM NaCl, 1 mM MgCl_2_, 1 mM EGTA, 1 mM DTT, 0.05% Triton X-100, 2.5 mM d-desthiobiotin). The sample was first reduced and alkylated by adding Tris(2-carboxyethyl)phosphine (TCEP) and chloroacetamide (CAA) to a final concentration of ~10 mM and 40 mM, respectively. Incubation first took place at 99 °C for 10 min, then in the dark for 20 min at r.t.

The sample was digested using the single-pot, solid-phase-enhance sample preparation method (SP3)^91^. For this, Cytiva Sera-Mag carboxylate-modified magnetic beads—hydrophilic and hydrophobic beads—were used in a 1:1 ratio. The beads were washed with ultrapure water (MQ), repeated twice. To ensure that the bead concentration remained above 0.5 µg μl^−1^, a total of 75 µg of beads were added to the sample, then 100% ethanol (EtOH) was added to a final concentration of 75% EtOH. The sample was incubated in a Thermomixer at 1,000 r.p.m. for 20 min at r.t. The tube was placed in a magnetic rack, and the supernatant removed. The beads were washed twice with 80% EtOH, followed by a washing step with 100% acetonitrile (ACN). For digestion, the beads were resuspended in 200 µl of 100 mM ammonium bicarbonate (AMBIC) by pushing the particles into solution; the digestion-bead solution was not pipetted or homogenized. The sample was then sonicated for 2 min in a water bath and digested using the proteases trypsin and lys-C (in 1:25 and 1:75 protease-to-protein ratios, respectively). Digestion was performed overnight in a Thermomixer at 1,000 r.p.m., at 37 °C. The sample was spun down and acidified by adding 10% trifluoracetic acid (TFA) to a final concentration of 5% TFA. The beads were immobilized on a magnetic rack, and the acidified peptide solution was transferred to a new Eppendorf tube. The peptide sample was dried in a vacuum centrifuge.

#### LC–MS/MS

Samples were resuspended in 50 µl of 2% formic acid (FA), then 5 µl was used for analysis by LC–MS/MS. Peptides were separated on a nanospray UHPLC system (Ultimate 3000, Thermo) using a 50-cm reversed-phase analytical column with an integrated emitter (75-µm inner diameter), packed in-house with ReproSilPur C18-AQ 1.9-µm resin (Dr Maisch). Mobile-phase buffer A was composed of 0.1% FA (vol/vol) and mobile-phase buffer B consisted of 80% ACN (vol/vol) and 0.1% FA (vol/vol). The Acclaim Pepmap 100 C18 (5 mm × 0.3 mm, 5 μm, Thermo) trap column was operated at a temperature of 32 °C, and the analytical column at a temperature of 50 °C. A flow rate of 0.3 μl min^−1^ was used. Starting at 4.0%, buffer B was increased over a total gradient time of 45 min using the following stepwise increases: 11% buffer B (3–35 min), 30% nuffer B (35–40 min), 44% buffer B (40–44 min) and 55% buffer B (44–45 min). The column was then washed out with 99% buffer B for 5 min, followed by a 10-min re-equilibration step with 4.0% buffer B.

The Ultimate 3000 system was coupled to a Thermo Scientific Orbitrap Exploris 480 instrument. Data were acquired in SII for Xcalibur software using a data-dependent mode (DDA), and MS1 scans were acquired at 60,000 resolution using a scan range of 375–1,600 *m*/*z*. With an isolation window of 1.4 *m*/*z*, precursor ions with an intensity threshold higher than 200,000 and charge states between 2+ and 6+ were selected for fragmentation. Precursors were fragmented by stepped higher-energy collisional dissociation (HCD) with a normalized collision energy (NCE) of 28%. The MS2 scans were acquired at 15,000 resolution, with maximum injection time set to auto, and the scan range set to a first mass of 120 *m*/*z*. The dynamic exclusion time was set to custom, with a 10-ppm upper and lower tolerance and excluding isotypes.

#### MaxQuant analysis

Raw data were analysed using MaxQuant-Andromeda software (v.2.4.7.0) for protein identification, using iBaq values for quantification. MS/MS spectra were searched against the UniProtKB Human Proteome (organism_id: 9606, reviewed, canonical & isoform FASTA, downloaded 12 March 2025) and a separate FASTA file containing the recombinant protein StrepII-GFP-GEF-H1. Default parameters were used for the precursor mass tolerance (20 ppm first search, 4.5 ppm main search), and the false discovery rate was set to 1%. Up to three missed cleavages for both proteases were allowed. Carbamidomethylation of cysteine residues was set as a fixed modification, and oxidation of methionine and acetylation of the protein N terminus were set as variable modifications, with a maximum of five modifications per peptide.

The iBaq values from the MaxQuant search were imported into Perseus software (v.2.0.11.0), and the protein groups were filtered out for ‘Reverse’ (false positives), ‘Contaminants’ and ‘Only identified by site’, as well as having only one peptide hit, and transferred to excel files for further analysis. The iBaq values were normalized to percentages, with the highest iBaq value set to 100%.

#### In vitro reconstitution assays

##### Sample preparation

The flow chambers were prepared by plasma-cleaning the coverslips and attaching them to glass slides using strips of double-sided tape to create chambers with a volume of ~10 µl. The surface was functionalized by 5-min incubations with 0.2 mg ml^−1^ PLL-PEG-biotin (Susos AG) and then 0.83 mg ml^−1^ neutravidin (Invitrogen), both dissolved in MRB80 buffer (80 mM K-Pipes, 1 mM EGTA, 4 mM MgCl_2_; pH 6.80 with KOH). Double-cycled biotinylated GMPCPP seeds prepared as described previously^53^ were then flowed in and incubated for 2 min, followed by a >3-min incubation with K-casein.

All tubulin used were from Cytoskeleton Inc. All mixes contained a freshly prepared master mix composed of 0.1% (wt/vol) methylcellulose, 0.5 mg ml^−1^ K-casein, 50 mM KCL, 50 mM glucose, 0.2 mg ml^−1^ catalase, 0.5 mg ml^−1^ glucose oxidase and 10 mM DTT, and were mixed in MRB80 buffer. For the GEF-H1 binding assays, the master mix was supplemented with 17 µM porcine brain tubulin, 0.6 µM TRITC rhodamine-labelled tubulin, 1 mM GTP/GTPyS and the required concentration of GEF-H1 proteins. For the paclitaxel and discodermolide expansion assays, the microtubule mix had master mix supplemented with 17 µM porcine brain tubulin, 0.6 µM TRITC rhodamine-labelled tubulin and 1 mM GTP, and the wash-in mix had master mix with 20 μM paclitaxel/200 nM discodermolide. For the experiments with GEF-H1 and paclitaxel/discodermolide, the microtubule mix was the same as for the binding assays, and the wash-in mix had 4.85 µM porcine brain tubulin, 0.15 μM TRITC rhodamine-labelled tubulin, 20 µM paclitaxel/200 nM discodermolide and the same concentration of GEF-H1 proteins. For the GTPyS-GDP parallel chamber experiment, fluorescent TetraSpeckTM beads were incubated for 2 min before PLL-PEG-biotin incubation. in parallel chambers prepared on the same coverslip. A second mix was made containing master mix, 17 µM porcine brain tubulin, 0.6 μM TRITC rhodamine-labelled tubulin and 500 pM GFP-GEF-H1. The mixture was divided equally, and either GTP or GTPγS was added to each half. All mixes were spun in an Airfuge set-up for 5 min at 20 psi. The microtubule mixes were flowed into the chambers, and the wash-in mixes were kept on ice before manually washing-in during image acquisition.

##### TIRF microscopy

TIRF microscopy was performed on an inverted Nikon Eclipse Ti-E research microscope equipped with azimuthal spinning TIRF illumination, a perfect focus system, and an ASI motorized MS-2000-XY stage. Imaging was carried out using a Nikon APO TIRF ×100/1.49 NA oil immersion objective in combination with an iLas2 illumination system (Roper Scientific, now Gataca Systems). Images were acquired with a CoolSNAP MYO CCD camera (Teledyne Photometrics), providing a pixel size of 0.045 μm pixel^−1^. Samples were maintained at 30 °C for all experiments using a Tokai Hit STXG-PLAMX-SETZ21L stage top incubator. Excitation was achieved using the following lasers: Coherent OBIS 561 nm (100 mW), Vortran Stradus 642 nm (110 mW) and Vortran Stradus 488 nm (150 mW). Emission was collected through the corresponding Chroma filters ET-GFP (49002), ET-mCherry (49008) and ET-647. Image acquisition was controlled using Metamorph software (version 7.10.2.240, Molecular Devices).

##### Image processing

To calculate intensity, videos were averaged over frames, and the mean intensity was calculated at the required region of interest. The ImageJ plugin KymoResliceWide v.04 was used to generate kymographs (https://github.com/ekatrukha/KymoResliceWide). To calculate lattice expansion, a straight line was drawn from the end of the bright seed to a speckle towards the plus end of the kymograph before wash-in. After flow-in, a second line was drawn between the same two points. The difference in lengths, reflecting the speckle shift, was used to calculate the percentage of expansion.

#### IRM to determine microtubule protofilament number

Porcine brain tubulin was isolated using the high-molarity PIPES method^92^. Biotin-labelled tubulin (Cytoskeleton Inc, T333P) was diluted 1:50 with unlabelled porcine brain tubulin to obtain biotin-labelled tubulin mix. Twelve protofilament microtubules were polymerized from 4 mg ml^−1^ of biotin-labelled tubulin for 1 h at 37 °C in 10 mM phosphate buffer supplemented with 6 mM MgCl_2_, 2 μM paclitaxel and 1 mM GTP (NU-1012, Jena Bioscience), according to ref. ^93^. Polymerized microtubules were centrifuged for 30 min at 18,000*g* in a Microfuge 18 centrifuge (Beckman Coulter). After centrifugation, the pellet was resuspended and kept in BRB80 supplemented with 10 μM paclitaxel at r.t. GMPCPP-stabilized 14-protofilament microtubules (and seeds for the dynamic microtubule assay) were polymerized from 4 mg ml^−1^ biotin-labelled tubulin for at least 3 h at 37 °C in BRB80 supplemented with 1 mM MgCl_2_ and 1 mM GMPCPP (Jena Bioscience, NU-405). Polymerized microtubules were centrifuged, and the pellet was resuspended as above.

For the imaging experiments, chambers were assembled by melting thin strips of parafilm between two glass coverslips silanized with hexamethyldisilazane (Sigma, 379212) and functionalized with 20 µg ml^−1^ anti-biotin antibodies (in BRB80; Sigma, B3640), incubated for 5 min, and 1% pluronic (F127 in BRB80; Sigma, P2443), incubated for at least 30 min.

TIRF microscopy was performed on an inverted Nikon Eclipse Ti2 (Nikon) equipped with a TIRF module and iLas illumination device (Gataca Systems), a perfect focus system and a motorized XY stage. Imaging was carried out using a Nikon APO TIRF ×60/1.49 NA oil immersion objective. Images were acquired with a PRIME BSI camera (Teledyne Photometrics), providing a pixel size of 0.072 × 0.072 μm^2^. Microtubules were visualized by IRM using a CoolLED pE-300 system, and excitation of fluorescently labelled tubulin was achieved using the Chroma TRF89901v2 ET 488-nm Laser Quad Band Set. Image acquisition was controlled using NIS-Elements software (version v5.42).

To measure the IRM signal of the microtubules, the background signal was first measured, then the 12-protofilament microtubules were immobilized in the chamber and their position imaged. The 14-protofilament GMPCPP-stabilized microtubules were then added to the chamber and the microtubules imaged. The dynamic microtubule assay was performed by immobilizing GMPCPP seeds on the coverslip surface, then 8 mg ml^−1^ of free fluorescent tubulin HiLyte488 (Cytoskeleton Inc, TL488M) was added in BRB80-based assay buffer (10 mM DTT, 20 mM D-glucose, 0.1% Tween 20, 0.05 mg ml^−1^ casein, 1 mM ATP, 1 mM GTP, glucose oxidase and catalase). Microtubule dynamics were measured for 5 min using both IRM and TIRF microcopy. The IRM signal of the microtubules was measured from the last frame.

The background-subtracted IRM signal, proportional to the local protein density, was used to analyse the microtubule thickness (protofilament number)^94^, then the mean IRM signal was measured by drawing a thick line along the microtubule. The resulting number was normalized to the signal of the 14-protofilament microtubules for the 12- versus 14-protofilament experiment as well as for the dynamic assay experiment.

#### Cryo-EM

##### Sample preparation

To ensure that the tubulin was fully depolymerized, after thawing, 50 µM porcine tubulin (Cytoskeleton) was incubated with 2 mM GTP in MRB80 on ice for 5 min. To reduce the formation of sheet-like structures, this mix was first polymerized for 10 min at 37 °C in the absence of discodermolide, followed by further polymerization for 20 min at 37 °C in the presence of 100 µM discodermolide. The polymerized microtubules were then pelleted using an Airfuge Air-Driven ultracentrifuge (5 min at 119,000*g*). The pellets were resuspended in MRB80 supplemented with 100 µM discodermolide to an approximate concentration of 8 µM tubulin (assuming 80% recovery). The concentration of polymerized tubulin was determined by depolymerization of a sample in MRB80 with 50 mM CaCl_2_ and 1 mM DTT, and measurement at *A*_280_ (extinction coefficient: 115,000 M^−1^ cm^−1^) on a NanoDrop instrument. Finally, discodermolide-bound microtubules were diluted to a concentration of 2 µM tubulin in MRB80 + 100 µM discodermolide.

##### Cryo-EM grid preparation and data acquisition

QUANTIFOIL holey carbon R3.5/1 Cu 200 mesh grids (Quantifoil Micro Tools) were glow-discharged at 25 mA for 45 s at 0.39 mbar (PELCO easiGlow), then 4 µl of discodermolide-stabilized microtubules were applied to the grid of an EM GP2 automatic plunge freezer (Leica) chamber. The grids were incubated at 30 °C and 95% humidity for 30 s, back-side blotted for 1.5 s and then plunged into liquid ethane. Data were recorded on a Titan Krios I set-up (The Netherlands Centre for Electron Nanoscopy (NeCEN), Leiden University, The Netherlands) equipped with a K3 summit direct electron detector and operated at 300 keV using EPU software. A magnification of 105,000×, corresponding to a nominal pixel size of 0.836 Å at the sample, was used, with a total dose of 50 e^−^/Å^2^ per exposure collected over 50 fractions (Supplementary Data 1). A defocus range of −0.6 to −2.0 µm was used (Supplementary Data 1).

##### Cryo-EM image processing

Videos were motion-corrected and CTF estimation was performed using cryoSPARC^95,96^ (Extended Data Fig. 6e). Approximately 500 particles from movies with varied defocus values and CTF estimations were manually picked, 2D-classified, and used as input for automatic filament tracing in cryoSPARC^95,96^. Filament tracing was performed with the separation distance set to the length of a single dimer, 82 Å. Particles were extracted in a box size twice as big as the microtubule filament diameter (~500 Å, 576 pixels) and binned by 2 (288 pixels). The particles were sorted into 11- to 16-protofilament classes by heterogeneous refinement. Particles belonging to 13- and 14-protofilament microtubules were selected for further separate processing. First, helical refinement was performed (twist = 0°, rise = 82.5 Å), followed by local refinement. Unbinned particles were then re-extracted in cryoSPARC^95,96^, and refined without any imposed symmetry (C1). The pixel size was calibrated in UCSF ChimeraX by maximizing the correlation of the final map with a map of a tubulin monomer generated from a previous tubulin structure (PDB 5XLT)^97^, to give a calibrated pixel size of 0.849 Å. Lattice parameters were calculated from a helical search of the C1 map (Extended Data Fig. 6f,g) using the Relion helix toolbox in Relion 5.0^98^. Molecular graphics and analyses performed with UCSF ChimeraX^99^.

##### Statistics and reproducibility

No statistical methods were used to predetermine sample sizes, but our sample sizes are similar to those reported in similar studies^71,73^. All experiments were performed on either cultured cells or purified proteins, so there was no variability introduced by our model that would need to be balanced with randomization. For data where most treatments fit a normal distribution and one group would fit a normal distribution with the removal of one or two outliers, these outliers were excluded. Researchers performing the analysis were not blind to the treatment groups associated with the data. In many cases this was for practical reasons, for example, to check that cells transfected with a microtubule acetylating enzyme indeed had acetylated microtubules. However, the researchers involved also had no preconceived ideas as to what the results should look like.

The Kolmogorov–Smirnov test was performed to determine whether the data fit a normal distribution for >4 data points and the Shapiro Wilk test was used for 3–4 data points. If data were determined not to fit a normal distribution, a two-tailed Mann–Whitney U-test was used to test for significance between treatment and control groups. Data on acetylated and non-acetylated GEF-H1 microtubule-to-cytoplasmic ratio were also determined not to follow a normal distribution. In this case, a two-tailed Wilcoxon matched-pairs signed rank test was used, as GEF-H1 binding on both acetylated and un-acetylated microtubules was quantified in each cell. If data were determined to follow a normal distribution, a two-tailed Welch’s *t*-test that does not assume equal variance was used to test for significance between treatment and control groups. A *P* value below 0.05 was determined to be significant for all of the above-mentioned tests.

### Reporting Summary

Further information on research design is available in the Nature Portfolio Reporting Summary linked to this Article.

## Online content

Any methods, additional references, Nature Portfolio reporting summaries, source data, extended data, supplementary information, acknowledgements, peer review information; details of author contributions and competing interests; and statements of data and code availability are available at https://doi.org/10.1038/s41556-026-02079-4.

## Supplementary information

Reporting Summary

Peer Review file

Supplementary Video 1 **Paclitaxel addition induces cell contraction and stress fibre formation in MDA-MB-231 cells**. MDA-MB-231 cells stably expressing the live-cell F-actin marker, Lifeact-mCherry (Lifeact-mCh), were imaged for 10 min before the addition of 10 µM paclitaxel, indicated by a pink border and ‘+PTX’, and subsequently imaged for another 1 h. Time is indicated as hh:mm.

Supplementary Video 2 **Discodermolide addition does not induce cell contraction or stress fibre formation in MDA-MB-231**. MDA-MB-231 cells stably expressing the live-cell F-actin marker, Lifeact-mCherry (Lifeact-mCh), were imaged for 10 min before the addition of 10 µM discodermolide, indicated by a blue border and ‘+DDM’, and subsequently imaged for another 1 h. Time is indicated as hh:mm.

Supplementary Video 3 **Paclitaxel addition induces cell contraction, blebbing and stress fibre formation in 1205Lu melanoma cells**. 1205Lu cells stably expressing the live-cell F-actin marker, eGFP-Lifeact, were imaged for 10 min before the addition of 10 µM paclitaxel, indicated by a pink border and ‘+PTX’, and subsequently imaged for another 1 h. Time is indicated as hh:mm.

Supplementary Video 4 **DMSO addition does not induce cell contraction, blebbing and stress fibre formation in 1205Lu cells**. 1205Lu cells stably expressing the live-cell F-actin marker, eGFP-Lifeact, were imaged for 10 min before the addition of DMSO, indicated by a blue border and ‘+DMSO’, and subsequently imaged for another 1 h. Time is indicated as hh:mm.

Supplementary Video 5 **Paclitaxel addition induces cell contraction, blebbing and stress fibre formation in HT1080 cells**. HT1080 cells transiently transfected with the cell F-actin marker, F-tractin-mStayGold, were imaged for 10 min before the addition of 10 µM paclitaxel, indicated by a pink border and ‘+PTX’, and subsequently imaged for another 1 h. Time is indicated as hh:mm.

Supplementary Video 6 **DMSO addition does not induce cell contraction, blebbing and stress fibre formation in HT1080 cells**. HT1080 cells transiently transfected with the cell F-actin marker, F-tractin-mStayGold, were imaged for 10 min before the addition of DMSO, indicated by a blue border and ‘+DMSO’, and subsequently imaged for another 1 h. Time is indicated as hh:mm.

Supplementary Video 7 **Paclitaxel addition displaces GFP-GEF-H1 from microtubules in U2OS cells**. U2OS cells transiently transfected with GFP-GEF-H1 (left) and mCherry-α-tubulin (mCh-α-tubulin; right) were imaged for 10 min before the addition of 10 µM paclitaxel, indicated by pink border and ‘+PTX’, and subsequently imaged for another 20 min. Time is indicated as mm:ss.

Supplementary Video 8 **Discodermolide addition does not displace GFP-GEF-H1 from microtubules in U2OS cells**. U2OS cells transiently transfected with GFP-GEF-H1 (left) and mCherry-α-tubulin (mCh-α-tubulin; right) were imaged for 10 min before the addition of 10 µM discodermolide, indicated by a blue border and ‘+DDM’, and subsequently imaged for another 20 min. Time is indicated as mm:ss.

Supplementary Video 9 **Paclitaxel addition displaces GFP-GEF-H1 from microtubules in HT1080 cells**. HT1080 cells transiently transfected with GFP-GEF-H1 (left) and mCherry-α-tubulin (mCh-α-tubulin; right) were imaged for 10 min before the addition of 10 µM paclitaxel, indicated by a pink border and ‘+PTX’, and subsequently imaged for another 10 min. Time is indicated as mm:ss.

Supplementary Video 10 **DMSO addition does not displace GFP-GEF-H1 from microtubules in HT1080 cells**. HT1080 cells transiently transfected with GFP-GEF-H1 (left) and mCherry-α-tubulin (mCh-α-tubulin; right) were imaged for 10 min before the addition of 10 µM discodermolide, indicated by a blue border and ‘+DMSO’, and subsequently imaged for another 10 min. Time is indicated as mm:ss.

Supplementary Table 1 **Mass spectrometry analysis of purified GFP-GEF-H1 protein**.

Supplementary Table 2 **Light microscopy reporting table**.

Supplementary Data 1 **Cryo-EM data collection, refinement and validation statistics**.

## Source data

Source Data Fig. 1 Statistical source data

Source Data Fig. 2 Statistical source data

Source Data Fig. 3 Statistical source data

Source Data Fig. 4 Statistical source data

Source Data Extended Data Fig./Table 2 Statistical source data

Source Data Extended Data Fig./Table 3 Statistical source data

Source Data Extended Data Fig./Table 4 Statistical source data

Source Data Extended Data Fig./Table 5 Statistical source data

Source Data Extended Data Fig./Table 6 Statistical source data

Unprocessed Blots Unprocessed western blots

## Data Availability

The cryo-EM maps for the discodermolide-stabilized 13-protofilament and 14-protofilament microtubules have been deposited in the EMDB under accession codes EMD-58509 and EMD-58507, respectively. The MS proteomics data have been deposited to the ProteomeXchange Consortium via the PRIDE^100^ partner repository with the dataset identifier PXD079708. Data are available from the authors on request. All unique and stable reagents generated in this study are available from the lead contact without restriction. Source data are provided with this paper.

## References

[1] Dumontet, C. & Jordan, M. A. Microtubule-binding agents: a dynamic field of cancer therapeutics. *Nat. Rev. Drug Discov.* **9**, 790–803 10.1038/nrd3253 (2010). PMC3194401 20885410

[2] Wilson, L. & Jordan, M. A. New microtubule/tubulin-targeted anticancer drugs and novel chemotherapeutic strategies. *J. Chemother.* **16**, 83–85 10.1179/joc.2004.16.Supplement-1.83 (2004). 15688618

[3] Jordan, M. A. & Wilson, L. Microtubules as a target for anticancer drugs. *Nat. Rev. Cancer* **4**, 253–265 10.1038/nrc1317 (2004). 15057285

[4] Steinmetz, M. O. & Prota, A. E. Microtubule-targeting agents: strategies to hijack the cytoskeleton. *Trends Cell Biol.* **28**, 776–792 10.1016/j.tcb.2018.05.001 (2018). 29871823

[5] Florian, S. & Mitchison, T. J. Anti-microtubule drugs. *Methods Mol. Biol.* **1413**, 403–421 10.1007/978-1-4939-3542-0_25 (2016). 27193863

[6] Perez, E. A. Microtubule inhibitors: differentiating tubulin-inhibiting agents based on mechanisms of action, clinical activity and resistance. *Mol. Cancer Ther.* **8**, 2086–2095 10.1158/1535-7163.MCT-09-0366 (2009). 19671735

[7] Jordan, M. A., Toso, R. J., Thrower, D. & Wilson, L. Mechanism of mitotic block and inhibition of cell proliferation by taxol at low concentrations. *Proc. Natl Acad. Sci. USA* **90**, 9552–9556 10.1073/pnas.90.20.9552 (1993). PMC47607 8105478

[8] Weaver, B. A. How Taxol/paclitaxel kills cancer cells. *Mol. Biol. Cell* **25**, 2677–2681 10.1091/mbc.E14-04-0916 (2014). PMC4161504 25213191

[9] Terzis, A. J. et al. Proliferation, migration and invasion of human glioma cells exposed to paclitaxel (Taxol) in vitro. *Br. J. Cancer* **75**, 1744–1752 10.1038/bjc.1997.298 (1997). PMC2223616 9192976

[10] Yang, H., Ganguly, A. & Cabral, F. Inhibition of cell migration and cell division correlates with distinct effects of microtubule inhibiting drugs. *J. Biol. Chem.* **285**, 32242–32250 10.1074/jbc.M110.160820 (2010). PMC2952225 20696757

[11] Komlodi-Pasztor, E., Sackett, D. L. & Fojo, A. T. Inhibitors targeting mitosis: tales of how great drugs against a promising target were brought down by a flawed rationale. *Clin. Cancer Res.* **18**, 51–63 10.1158/1078-0432.CCR-11-0999 (2012). 22215906

[12] Shi, J. & Mitchison, T. J. Cell death response to anti-mitotic drug treatment in cell culture, mouse tumor model and the clinic. *Endocr. Relat. Cancer* **24**, T83–T96 10.1530/ERC-17-0003 (2017). PMC5557680 28249963

[13] Janssen, A., Beerling, E., Medema, R. & van Rheenen, J. Intravital FRET imaging of tumor cell viability and mitosis during chemotherapy. *PLoS ONE* **8**, e64029 10.1371/journal.pone.0064029 (2013). PMC3654962 23691140

[14] Mertens, S. et al. Drug-repurposing screen on patient-derived organoids identifies therapy-induced vulnerability in KRAS-mutant colon cancer. *Cell Rep.* **42**, 112324 10.1016/j.celrep.2023.112324 (2023). 37000626

[15] Hellal, F. et al. Microtubule stabilization reduces scarring and causes axon regeneration after spinal cord injury. *Science* **331**, 928–931 10.1126/science.1201148 (2011). PMC3330754 21273450

[16] Tomasek, J. J. & Hay, E. D. Analysis of the role of microfilaments and microtubules in acquisition of bipolarity and elongation of fibroblasts in hydrated collagen gels. *J. Cell Biol.* **99**, 536–549 10.1083/jcb.99.2.536 (1984). PMC2113263 6146628

[17] Horin, L. J., Sonnett, M., Li, B. & Mitchison, T. J. Diverse microtubule-destabilizing drugs induce equivalent molecular pathway responses in endothelial cells. Preprint at 10.1101/2025.01.22.632572 (2025).

[18] Krendel, M., Zenke, F. T. & Bokoch, G. M. Nucleotide exchange factor GEF-H1 mediates cross-talk between microtubules and the actin cytoskeleton. *Nat. Cell Biol.* **4**, 294–301 (2002). 10.1038/ncb773 11912491

[19] Kashyap, A. S. et al. GEF-H1 signaling upon microtubule destabilization is required for dendritic cell activation and specific anti-tumor responses. *Cell Rep.* **28**, 3367–3380 10.1016/j.celrep.2019.08.057 (2019). PMC6876861 31553907

[20] Birkenfeld, J., Nalbant, P., Yoon, S. H. & Bokoch, G. M. Cellular functions of GEF-H1, a microtubule-regulated Rho-GEF: is altered GEF-H1 activity a crucial determinant of disease pathogenesis? *Trends Cell Biol.* **18**, 210–219 10.1016/j.tcb.2008.02.006 (2008). 18394899

[21] Azoitei, M. L. et al. Spatiotemporal dynamics of GEF-H1 activation controlled by microtubule- and Src-mediated pathways. *J. Cell Biol.* **218**, 3077–3097 10.1083/jcb.201812073 (2019). PMC6719461 31420453

[22] Choi, S. R. et al. Structural basis of microtubule-mediated signal transduction. *Cell* **189**, 461–477 10.1016/j.cell.2025.11.011 (2026). 41365297

[23] Tsapara, A. et al. The RhoA activator GEF-H1/Lfc is a transforming growth factor-beta target gene and effector that regulates alpha-smooth muscle actin expression and cell migration. *Mol. Biol. Cell* **21**, 860–870 10.1091/mbc.e09-07-0567 (2010). PMC2836967 20089843

[24] Heck, J. N. et al. Microtubules regulate GEF-H1 in response to extracellular matrix stiffness. *Mol. Biol. Cell* **23**, 2583–2592 10.1091/mbc.E11-10-0876 (2012). PMC3386221 22593214

[25] Nalbant, P., Chang, Y. C., Birkenfeld, J., Chang, Z. F. & Bokoch, G. M. Guanine nucleotide exchange factor-H1 regulates cell migration via localized activation of RhoA at the leading edge. *Mol. Biol. Cell* **20**, 4070–4082 10.1091/mbc.e09-01-0041 (2009). PMC2743625 19625450

[26] Kopf, A. et al. Microtubules control cellular shape and coherence in amoeboid migrating cells. *J. Cell Biol.* **219**, e201907154 10.1083/jcb.201907154 (2020). PMC7265309 32379884

[27] Cullis, J. et al. The RhoGEF GEF-H1 is required for oncogenic RAS signaling via KSR-1. *Cancer Cell* **25**, 181–195 10.1016/j.ccr.2014.01.025 (2014). 24525234

[28] Cheng, I. K. et al. GEF-H1 over-expression in hepatocellular carcinoma promotes cell motility via activation of RhoA signalling. *J. Pathol.* **228**, 575–585 10.1002/path.4084 (2012). 22847784

[29] Zhu, Y. et al. ARHGEF2/EDN1 pathway participates in ER stress-related drug resistance of hepatocellular carcinoma by promoting angiogenesis and malignant proliferation. *Cell Death Dis.* **13**, 652 10.1038/s41419-022-05099-8 (2022). PMC9329363 35896520

[30] de Seze, J., Gatin, J. & Coppey, M. RhoA regulation in space and time. *FEBS Lett.* **597**, 836–849 10.1002/1873-3468.14578 (2023). 36658753

[31] Magiera, M. M., Singh, P., Gadadhar, S. & Janke, C. Tubulin posttranslational modifications and emerging links to human disease. *Cell* **173**, 1323–1327 10.1016/j.cell.2018.05.018 (2018). 29856952

[32] Seetharaman, S. et al. Microtubules tune mechanosensitive cell responses. *Nat. Mater.* **21**, 366–377 10.1038/s41563-021-01108-x (2022). 34663953

[33] Deb Roy, A. et al. OptoTAT reveals microtubule acetylation as a rapid trigger for GEF-H1–mediated cell migration. *J. Cell Biol.* **225**, e202508095 10.1083/jcb.202508095 (2026). PMC13004174 41854498

[34] Howes, S. C., Alushin, G. M., Shida, T., Nachury, M. V. & Nogales, E. Effects of tubulin acetylation and tubulin acetyltransferase binding on microtubule structure. *Mol. Biol. Cell* **25**, 257–266 10.1091/mbc.E13-07-0387 (2014). PMC3890346 24227885

[35] Luo, J. et al. Tubulin acetyltransferases access and modify the microtubule luminal K40 residue through anchors in taxane-binding pockets. *Nat. Struct. Mol. Biol.* **32**, 358–368 10.1038/s41594-024-01406-3 (2025). 39496813

[36] Iuzzolino, A., Pellegrini, F. R., Rotili, D., Degrassi, F. & Trisciuoglio, D. The alpha-tubulin acetyltransferase ATAT1: structure, cellular functions, and its emerging role in human diseases. *Cell. Mol. Life Sci.* **81**, 193 10.1007/s00018-024-05227-x (2024). PMC11039541 38652325

[37] Piperno, G., LeDizet, M. & Chang, X. J. Microtubules containing acetylated alpha-tubulin in mammalian cells in culture. *J. Cell Biol.* **104**, 289–302 10.1083/jcb.104.2.289 (1987). PMC2114420 2879846

[38] Cambray-Deakin, M. A. & Burgoyne, R. D. Acetylated and detyrosinated alpha-tubulins are co-localized in stable microtubules in rat meningeal fibroblasts. *Cell Motil. Cytoskeleton* **8**, 284–291 10.1002/cm.970080309 (1987). 3319198

[39] de Jager, L. et al. StableMARK-decorated microtubules in cells have expanded lattices. *J. Cell Biol.* **224**, e202206143 10.1083/jcb.202206143 (2025). PMC11471893 39387699

[40] Alushin, G. M. et al. High-resolution microtubule structures reveal the structural transitions in αβ-tubulin upon GTP hydrolysis. *Cell* **157**, 1117–1129 10.1016/j.cell.2014.03.053 (2014). PMC4054694 24855948

[41] Lucena-Agell, D. et al. An atlas of microtubule lattice parameters regulated through ligand binding to the microtubule-stabilizing sites. *Proc. Natl Acad. Sci. USA* **123**, e2532791123 10.1073/pnas.2532791123 (2026). PMC13291687 42301786

[42] Fritz, R. D. et al. A versatile toolkit to produce sensitive FRET biosensors to visualize signaling in time and space. *Sci. Signal.* **6**, rs12 10.1126/scisignal.2004135 (2013). 23882122

[43] Ridley, A. J. & Hall, A. The small GTP-binding protein rho regulates the assembly of focal adhesions and actin stress fibers in response to growth factors. *Cell* **70**, 389–399 10.1016/0092-8674(92)90163-7 (1992). 1643657

[44] Moolenaar, W. H. Lysophosphatidic acid, a multifunctional phospholipid messenger. *J. Biol. Chem.* **270**, 12949–12952 10.1074/jbc.270.22.12949 (1995). 7768880

[45] Longley, R. E., Caddigan, D., Harmody, D., Gunasekera, M. & Gunasekera, S. P. Discodermolide—a new, marine-derived immunosuppressive compound. I. In vitro studies. *Transplantation* **52**, 650–656 10.1097/00007890-199110000-00014 (1991). 1833864

[46] Buey, R. M. et al. Microtubule interactions with chemically diverse stabilizing agents: thermodynamics of binding to the paclitaxel site predicts cytotoxicity. *Chem. Biol.* **12**, 1269–1279 10.1016/j.chembiol.2005.09.010 (2005). 16356844

[47] Gardner, E. R. et al. Randomized crossover pharmacokinetic study of solvent-based paclitaxel and *nab*-paclitaxel. *Clin. Cancer Res.* **14**, 4200–4205 10.1158/1078-0432.CCR-07-4592 (2008). PMC2661025 18594000

[48] Brouwer, E. et al. Measurement of fraction unbound paclitaxel in human plasma. *Drug Metab. Dispos.* **28**, 1141–1145 10.1016/S0026-895X(24)12040-8 (2000). 10997930

[49] Imran, M., Saleem, S., Chaudhuri, A., Ali, J. & Baboota, S. Docetaxel: an update on its molecular mechanisms, therapeutic trajectory and nanotechnology in the treatment of breast, lung and prostate cancer. *J. Drug Delivery Sci. Technol.* **60**, 101959 10.1016/j.jddst.2020.101959 (2020).

[50] Amano, M., Nakayama, M. & Kaibuchi, K. Rho-kinase/ROCK: a key regulator of the cytoskeleton and cell polarity. *Cytoskeleton (Hoboken)* **67**, 545–554 10.1002/cm.20472 (2010). PMC3038199 20803696

[51] Lawson, C. D. & Ridley, A. J. Rho GTPase signaling complexes in cell migration and invasion. *J. Cell Biol.* **217**, 447–457 10.1083/jcb.201612069 (2018). PMC5800797 29233866

[52] Riento, K. & Ridley, A. J. Rocks: multifunctional kinases in cell behaviour. *Nat. Rev. Mol. Cell Biol.* **4**, 446–456 10.1038/nrm1128 (2003). 12778124

[53] Jansen, K. I., Iwanski, M. K., Burute, M. & Kapitein, L. C. A live-cell marker to visualize the dynamics of stable microtubules throughout the cell cycle. *J. Cell Biol.* **222**, e202106105 10.1083/jcb.202106105 (2023). PMC9998657 36880745

[54] Haggarty, S. J., Koeller, K. M., Wong, J. C., Grozinger, C. M. & Schreiber, S. L. Domain-selective small-molecule inhibitor of histone deacetylase 6 (HDAC6)-mediated tubulin deacetylation. *Proc. Natl Acad. Sci. USA* **100**, 4389–4394 10.1073/pnas.0430973100 (2003). PMC153564 12677000

[55] Akella, J. S. et al. MEC-17 is an alpha-tubulin acetyltransferase. *Nature* **467**, 218–222 10.1038/nature09324 (2010). PMC2938957 20829795

[56] Aillaud, C. et al. Vasohibins/SVBP are tubulin carboxypeptidases (TCPs) that regulate neuron differentiation. *Science* **358**, 1448–1453 10.1126/science.aao4165 (2017). 29146868

[57] Nieuwenhuis, J. et al. Vasohibins encode tubulin detyrosinating activity. *Science* **358**, 1453–1456 10.1126/science.aao5676 (2017). 29146869

[58] Landskron, L. et al. Posttranslational modification of microtubules by the MATCAP detyrosinase. *Science* **376**, eabn6020 10.1126/science.abn6020 (2022). 35482892

[59] Bieling, P. et al. Reconstitution of a microtubule plus-end tracking system in vitro. *Nature* **450**, 1100–1105 10.1038/nature06386 (2007). 18059460

[60] Saunders, H. A. J. et al. A network of interacting ciliary tip proteins with opposing activities imparts slow and processive microtubule growth. *Nat. Struct. Mol. Biol.* **32**, 979–994 10.1038/s41594-025-01483-y (2025). PMC12170345 39856351

[61] Hyman, A. A., Chretien, D., Arnal, I. & Wade, R. H. Structural changes accompanying GTP hydrolysis in microtubules: information from a slowly hydrolyzable analogue guanylyl-(alpha,beta)-methylene-diphosphonate. *J. Cell Biol.* **128**, 117–125 10.1083/jcb.128.1.117 (1995). PMC2120325 7822409

[62] Rai, A. et al. Lattice defects induced by microtubule-stabilizing agents exert a long-range effect on microtubule growth by promoting catastrophes. *Proc. Natl Acad. Sci. USA* **118**, e2112261118 10.1073/pnas.2112261118 (2021). PMC8713758 34916292

[63] Chaaban, S. & Brouhard, G. J. A microtubule bestiary: structural diversity in tubulin polymers. *Mol. Biol. Cell* **28**, 2924–2931 10.1091/mbc.E16-05-0271 (2017). PMC5662251 29084910

[64] Hung, D. T., Chen, J. & Schreiber, S. L. (+)-Discodermolide binds to microtubules in stoichiometric ratio to tubulin dimers, blocks taxol binding and results in mitotic arrest. *Chem. Biol.* **3**, 287–293 10.1016/s1074-5521(96)90108-8 (1996). 8807856

[65] ter Haar, E. et al. Discodermolide, a cytotoxic marine agent that stabilizes microtubules more potently than taxol. *Biochemistry* **35**, 243–250 10.1021/bi9515127 (1996). 8555181

[66] Kellogg, E. H. et al. Insights into the distinct mechanisms of action of taxane and non-taxane microtubule stabilizers from cryo-EM structures. *J. Mol. Biol.* **429**, 633–646 10.1016/j.jmb.2017.01.001 (2017). PMC5325780 28104363

[67] Zhang, R., LaFrance, B. & Nogales, E. Separating the effects of nucleotide and EB binding on microtubule structure. *Proc. Natl Acad. Sci. USA* **115**, E6191–E6200 10.1073/pnas.1802637115 (2018). PMC6142192 29915050

[68] Janke, C. & Magiera, M. M. The tubulin code and its role in controlling microtubule properties and functions. *Nat. Rev. Mol. Cell Biol.* **21**, 307–326 10.1038/s41580-020-0214-3 (2020). 32107477

[69] Verhey, K. J. & Ohi, R. Causes, costs and consequences of kinesin motors communicating through the microtubule lattice. *J. Cell Sci.* **136**, jcs260735 10.1242/jcs.260735 (2023). PMC10022682 36866642

[70] Akhmanova, A. & Kapitein, L. C. Mechanisms of microtubule organization in differentiated animal cells. *Nat. Rev. Mol. Cell Biol.* **23**, 541–558 10.1038/s41580-022-00473-y (2022). 35383336

[71] Paquette, A. L. et al. Competition for microtubule lattice spacing between a microtubule expander and compactor. *Curr. Biol.* **35**, 4442–4452 e4444 10.1016/j.cub.2025.07.080 (2025). 40885194

[72] LaFrance, B. J. et al. Structural transitions in the GTP cap visualized by cryo-electron microscopy of catalytically inactive microtubules. *Proc. Natl Acad. Sci. USA* **119**, e2114994119 10.1073/pnas.2114994119 (2022). PMC8764682 34996871

[73] Siahaan, V. et al. Microtubule lattice spacing governs cohesive envelope formation of tau family proteins. *Nat. Chem. Biol.* **18**, 1224–1235 10.1038/s41589-022-01096-2 (2022). PMC9613621 35996000

[74] Stehbens, S. & Wittmann, T. Targeting and transport: how microtubules control focal adhesion dynamics. *J. Cell Biol.* **198**, 481–489 10.1083/jcb.201206050 (2012). PMC3514042 22908306

[75] Bros, M., Haas, K., Moll, L. & Grabbe, S. RhoA as a key regulator of innate and adaptive immunity. *Cells* **8**, 733 10.3390/cells8070733 (2019). PMC6678964 31319592

[76] Kalim, K. W. et al. Graded RhoA GTPase expression in Treg cells distinguishes tumor immunity from autoimmunity. *Front. Immunol.* **12**, 726393 10.3389/fimmu.2021.726393 (2021). PMC8554290 34721389

[77] Martin-Ibanez, R. et al. Novel cryopreservation method for dissociated human embryonic stem cells in the presence of a ROCK inhibitor. *Hum. Reprod.* **23**, 2744–2754 10.1093/humrep/den316 (2008). 18716037

[78] Sato, T. et al. Single Lgr5 stem cells build crypt-villus structures in vitro without a mesenchymal niche. *Nature* **459**, 262–265 10.1038/nature07935 (2009). 19329995

[79] Koyanagi, M. et al. Inhibition of the Rho/ROCK pathway reduces apoptosis during transplantation of embryonic stem cell-derived neural precursors. *J. Neurosci. Res.* **86**, 270–280 10.1002/jnr.21502 (2008). 17828770

[80] Minambres, R., Guasch, R. M., Perez-Arago, A. & Guerri, C. The RhoA/ROCK-I/MLC pathway is involved in the ethanol-induced apoptosis by anoikis in astrocytes. *J. Cell Sci.* **119**, 271–282 10.1242/jcs.02723 (2006). 16390872

[81] Walker, A. et al. Non-muscle myosin II regulates survival threshold of pluripotent stem cells. *Nat. Commun.* **1**, 71 10.1038/ncomms1074 (2010). PMC3430968 20842192

[82] McCray, B. A. et al. Neuropathy-causing TRPV4 mutations disrupt TRPV4-RhoA interactions and impair neurite extension. *Nat. Commun.* **12**, 1444 10.1038/s41467-021-21699-y (2021). PMC7933254 33664271

[83] Ohsawa, M., Aasato, M., Hayashi, S. S. & Kamei, J. RhoA/Rho kinase pathway contributes to the pathogenesis of thermal hyperalgesia in diabetic mice. *Pain* **152**, 114–122 10.1016/j.pain.2010.10.005 (2011). 20980102

[84] Bouchet, B. P. et al. Mesenchymal cell invasion requires cooperative regulation of persistent microtubule growth by SLAIN2 and CLASP1. *Dev. Cell* **39**, 708–723 10.1016/j.devcel.2016.11.009 (2016). PMC5178967 27939686

[85] Ju, R. J. et al. Compression-dependent microtubule reinforcement enables cells to navigate confined environments. *Nat. Cell Biol.* **26**, 1520–1534 10.1038/s41556-024-01476-x (2024). PMC13186247 39160291

[86] Riedl, J. et al. Lifeact: a versatile marker to visualize F-actin. *Nat. Methods* **5**, 605–607 10.1038/nmeth.1220 (2008). PMC2814344 18536722

[87] Johnson, H. W. & Schell, M. J. Neuronal IP3 3-kinase is an F-actin-bundling protein: role in dendritic targeting and regulation of spine morphology. *Mol. Biol. Cell* **20**, 5166–5180 10.1091/mbc.e09-01-0083 (2009). PMC2793293 19846664

[88] Ando, R. et al. StayGold variants for molecular fusion and membrane-targeting applications. *Nat. Methods* **21**, 648–656 10.1038/s41592-023-02085-6 (2024). PMC11009113 38036853

[89] van der Vaart, B. et al. SLAIN2 links microtubule plus end-tracking proteins and controls microtubule growth in interphase. *J. Cell Biol.* **193**, 1083–1099 10.1083/jcb.201012179 (2011). PMC3115796 21646404

[90] Ran, F. A. et al. Genome engineering using the CRISPR-Cas9 system. *Nat. Protoc.* **8**, 2281–2308 10.1038/nprot.2013.143 (2013). PMC3969860 24157548

[91] Hughes, C. S. et al. Single-pot, solid-phase-enhanced sample preparation for proteomics experiments. *Nat. Protoc.* **14**, 68–85 10.1038/s41596-018-0082-x (2019). 30464214

[92] Castoldi, M. & Popov, A. V. Purification of brain tubulin through two cycles of polymerization-depolymerization in a high-molarity buffer. *Protein Expr. Purif.* **32**, 83–88 10.1016/S1046-5928(03)00218-3 (2003). 14680943

[93] Ray, S., Meyhofer, E., Milligan, R. A. & Howard, J. Kinesin follows the microtubule’s protofilament axis. *J. Cell Biol.* **121**, 1083–1093 10.1083/jcb.121.5.1083 (1993). PMC2119687 8099076

[94] Mahamdeh, M. & Howard, J. Implementation of interference reflection microscopy for label-free, high-speed imaging of microtubules. *J. Vis. Exp* 10.3791/59520 (2019). PMC6858481 31449260

[95] Rubinstein, J. L. & Brubaker, M. A. Alignment of cryo-EM movies of individual particles by optimization of image translations. *J. Struct. Biol.* **192**, 188–195 10.1016/j.jsb.2015.08.007 (2015). 26296328

[96] Punjani, A., Rubinstein, J. L., Fleet, D. J. & Brubaker, M. A. cryoSPARC: algorithms for rapid unsupervised cryo-EM structure determination. *Nat. Methods* **14**, 290–296 10.1038/nmeth.4169 (2017). 28165473

[97] Prota, A. E. et al. Structural basis of microtubule stabilization by discodermolide. *ChemBioChem* **18**, 905–909 10.1002/cbic.201600696 (2017). PMC5559732 28207984

[98] He, S. & Scheres, S. H. W. Helical reconstruction in RELION. *J. Struct. Biol.* **198**, 163–176 10.1016/j.jsb.2017.02.003 (2017). PMC5479445 28193500

[99] Meng, E. C. et al. UCSF ChimeraX: tools for structure building and analysis. *Protein Sci.* **32**, e4792 10.1002/pro.4792 (2023). PMC10588335 37774136

[100] Perez-Riverol, Y. et al. The PRIDE database at 20 years: 2025 update. *Nucleic Acids Res.* **53**, D543–D553 10.1093/nar/gkae1011 (2025). PMC11701690 39494541

[101] Debs, G. E., Cha, M., Liu, X., Huehn, A. R. & Sindelar, C. V. Dynamic and asymmetric fluctuations in the microtubule wall captured by high-resolution cryoelectron microscopy. *Proc. Natl Acad. Sci. USA* **117**, 16976–16984 10.1073/pnas.2001546117 (2020). PMC7382274 32636254

