# Supplementary material for: Taxane-induced conformational changes in the microtubule lattice activate GEF-H1-dependent RhoA signalling
Source: Nat Cell Biol. 2026 Sep 24;28(11):2219–30. doi: 10.1038/s41556-026-02079-4 (PMC13751295; doi:10.1038/s41556-026-02079-4)
Supplement: Supplementary file 1 [file 41556_2026_2079_MOESM15_ESM.pdf]

Cryo-EM data collection, refinement and validation statistics

	#1 Discodermolide stabilised 13-protofilament microtubule C1 reconstruction (EMD-58509)	#2 Discodermolide stabilised 14-protofilament microtubule C1 reconstruction (EMD-58507)
**Data collection and processing**		
Magnification	105000	105000
Voltage (kV)	300	300
Electron exposure (e–/Å2)	50	50
Defocus range (μm)	-0.6 to -2.0	-0.6 to -2.0
Pixel size (Å)	0.849	0.849
Symmetry imposed	C1	C1
Initial particle images (no.)	820009	820009
Final particle images (no.)	164768	391452
Map resolution (Å)	3.47	2.73
FSC threshold	(0.143)	(0.143)
Map resolution range (Å)	1.81 to 27.17	1.84 to 4.60
