# Supplementary material for: Taxane-induced conformational changes in the microtubule lattice activate GEF-H1-dependent RhoA signalling
Source: Nat Cell Biol. 2026 Sep 24;28(11):2219–30. doi: 10.1038/s41556-026-02079-4 (PMC13751295; doi:10.1038/s41556-026-02079-4)
Supplement: Supplementary file 2 [file 41556_2026_2079_MOESM1_ESM.pdf]

nature portfolio

Corresponding author(s): Anna Akhmanova, Joyce C. M. Meiring

Last updated by author(s): Aug 11, 2026

Reporting Summary

Nature Portfolio wishes to improve the reproducibility of the work that we publish. This form provides structure for consistency and transparency in reporting. For further information on Nature Portfolio policies, see our Editorial Policies and the Editorial Policy Checklist.

Statistics

For all statistical analyses, confirm that the following items are present in the figure legend, table legend, main text, or Methods section.

n/a	Confirmed	
☐	☒	The exact sample size (*n*) for each experimental group/condition, given as a discrete number and unit of measurement
☐	☒	A statement on whether measurements were taken from distinct samples or whether the same sample was measured repeatedly
☐	☒	The statistical test(s) used AND whether they are one- or two-sided<br>*Only common tests should be described solely by name; describe more complex techniques in the Methods section.*
☒	☐	A description of all covariates tested
☐	☒	A description of any assumptions or corrections, such as tests of normality and adjustment for multiple comparisons
☐	☒	A full description of the statistical parameters including central tendency (e.g. means) or other basic estimates (e.g. regression coefficient) AND variation (e.g. standard deviation) or associated estimates of uncertainty (e.g. confidence intervals)
☐	☒	For null hypothesis testing, the test statistic (e.g. *F*, *t*, *r*) with confidence intervals, effect sizes, degrees of freedom and *P* value noted<br>*Give P values as exact values whenever suitable.*
☒	☐	For Bayesian analysis, information on the choice of priors and Markov chain Monte Carlo settings
☒	☐	For hierarchical and complex designs, identification of the appropriate level for tests and full reporting of outcomes
☒	☐	Estimates of effect sizes (e.g. Cohen's *d*, Pearson's *r*), indicating how they were calculated

Our web collection on statistics for biologists contains articles on many of the points above.

Software and code

Policy information about availability of computer code

Data collection	Microscope acquisition software: RhoA2G FRET experiments with ZEN black v2.3; Live F-Chitax3 accumulation/impact of Paclitaxel/Discodermolide on GEFH1 in U2OS/Lifeact-mCh in MDA-MB-231 imaging with MetaMorph v7.10; Live cell GFP-Lifeact imaging in 1205Lu, F-tractin-mStayGold imaging in HT1080, GFP-GEF-H1 and mCh-a-tubulin imaging in HT1080 with Fusion software v2.3.0.36; Actin and microtubule staining widefield images for quantification using Nikon NIS Br, confocal images for figure panels with LAS X; TIRF in vitro assays with MetaMorph v7.10, IRM in vitro assays with NIS-Elements v5.42<br>Western blot imaging software: IQ800 control software<br>Mass spectrometry: SII for Xcalibur<br>Cryo Electron Microscopy: EPU
Data analysis	All microscopy data and westernblot data except for Cryo Electron microscopy was quantified in ImageJ.<br>ImageJ plugins: KymoResliceWide v.04 (https://imagej.net/plugins/kymoreslicewide), template matching (https://imagej.net/ij/plugins/template-matching.html)<br>Output was further processed in Excel and graphs were made using Graphpad Prism v8.4.3<br>Cryo Electron Microscopy data processing: cryoSPARC<br>Mass spectrometry data processing: MaxQuant-Andromeda v.2.4.7.0 & Perseus v.2.0.11.0

For manuscripts utilizing custom algorithms or software that are central to the research but not yet described in published literature, software must be made available to editors and reviewers. We strongly encourage code deposition in a community repository (e.g. GitHub). See the Nature Portfolio guidelines for submitting code & software for further information.

Data

Policy information about availability of data

All manuscripts must include a data availability statement. This statement should provide the following information, where applicable:

- Accession codes, unique identifiers, or web links for publicly available datasets
- A description of any restrictions on data availability
- For clinical datasets or third party data, please ensure that the statement adheres to our policy

The cryo-EM maps for the discodermolide stabilized 13-protofilament and 14-protofilament microtubules have been deposited in the EMDB under the accession codes EMD-58509 and EMD-58507, respectively. The mass spectrometry proteomics data have been deposited to the ProteomeXchange Consortium via the PRIDE 100 partner repository with the dataset identifier PXD079708. Numerical source data are available in this manuscript; all other data are available from the authors on request. All unique and stable reagents generated in this study are available from the lead contact without restriction.

Research involving human participants, their data, or biological material

Policy information about studies with human participants or human data. See also policy information about sex, gender (identity/presentation), and sexual orientation and race, ethnicity and racism.

Reporting on sex and gender	*Use the terms sex (biological attribute) and gender (shaped by social and cultural circumstances) carefully in order to avoid confusing both terms. Indicate if findings apply to only one sex or gender; describe whether sex and gender were considered in study design; whether sex and/or gender was determined based on self-reporting or assigned and methods used. Provide in the source data disaggregated sex and gender data, where this information has been collected, and if consent has been obtained for sharing of individual-level data; provide overall numbers in this Reporting Summary. Please state if this information has not been collected. Report sex- and gender-based analyses where performed, justify reasons for lack of sex- and gender-based analysis.*
Reporting on race, ethnicity, or other socially relevant groupings	*Please specify the socially constructed or socially relevant categorization variable(s) used in your manuscript and explain why they were used. Please note that such variables should not be used as proxies for other socially constructed/relevant variables (for example, race or ethnicity should not be used as a proxy for socioeconomic status). Provide clear definitions of the relevant terms used, how they were provided (by the participants/respondents, the researchers, or third parties), and the method(s) used to classify people into the different categories (e.g. self-report, census or administrative data, social media data, etc.) Please provide details about how you controlled for confounding variables in your analyses.*
Population characteristics	*Describe the covariate-relevant population characteristics of the human research participants (e.g. age, genotypic information, past and current diagnosis and treatment categories). If you filled out the behavioural & social sciences study design questions and have nothing to add here, write "See above."*
Recruitment	*Describe how participants were recruited. Outline any potential self-selection bias or other biases that may be present and how these are likely to impact results.*
Ethics oversight	*Identify the organization(s) that approved the study protocol.*

Note that full information on the approval of the study protocol must also be provided in the manuscript.

Field-specific reporting

Please select the one below that is the best fit for your research. If you are not sure, read the appropriate sections before making your selection.

☒ Life sciences ☐ Behavioural & social sciences ☐ Ecological, evolutionary & environmental sciences

For a reference copy of the document with all sections, see nature.com/documents/nr-reporting-summary-flat.pdf

Life sciences study design

All studies must disclose on these points even when the disclosure is negative.

Sample size	Sample size was limited to practical resource and time constraints, where possible positive controls were used to test that true differences would be quantifiable. Given that we were able to measure significant differences between treatments with our assays we believe the sample sizes have been justified.
Data exclusions	In order to ensure comparable GFP-GEFH1 expression levels between treatments upper and lower thresholds for GFP-GEFH1 signal were set for each experiment and cells with GFP-GEFH1 signal above and below the threshold were excluded. For data where most treatments fit a normal distribution and 1 group would fit a normal distribution with the removal of 1 or 2 outliers, these outliers were excluded.
Replication	All experiments with the exception of the F-Chitax3 slow accumulation on microtubules experiment and the EB1+EB3 staining with DMSO/ Paclitaxel/Discodermolde treatment was repeated at least 3 times. The EB1+EB3 comet controls and the F-Chitax3 experiment did not show any unexpected new insights, nor were quantifications used for statistical analyses, hence repetition was not deemed necessary.

Randomization	All experiments were performed on either cultured cells or purified proteins so there was no variability introduced by our model that would need to be balanced with randomization.
Blinding	Researchers performing the analysis were not blind to the treatment groups associated with the data. In many cases this was also for practical reasons, for example to check that cells transfected with a microtubule acetylating enzyme indeed had acetylated microtubules. However the researchers involved also had no preconceived ideas as to what the results should look like.

Reporting for specific materials, systems and methods

We require information from authors about some types of materials, experimental systems and methods used in many studies. Here, indicate whether each material, system or method listed is relevant to your study. If you are not sure if a list item applies to your research, read the appropriate section before selecting a response.

Materials & experimental systems

n/a	Involved in the study	
☐	☒	Antibodies
☐	☒	Eukaryotic cell lines
☒	☐	Palaeontology and archaeology
☒	☐	Animals and other organisms
☒	☐	Clinical data
☒	☐	Dual use research of concern
☒	☐	Plants

Methods

n/a	Involved in the study	
☒	☐	ChIP-seq
☒	☐	Flow cytometry
☒	☐	MRI-based neuroimaging

Antibodies

Antibodies used	rabbit anti-p-MLC (Cell Signaling, 3671S), rabbit anti-GEF-H1 (Abcam, ab155785), Rat anti-tyrosinated-α-tubulin (Thermo, MA1-80017), Mouse anti-α-tubulin (Sigma, T6199), Mouse anti-acetylated-tubulin (Sigma, T7451), rabbit anti-de-tyrosinated-tubulin (Abcam, ab48389), rat EB1 + EB3 (Absea, 15H11), mouse anti GFP (Sigma, 11814460001)
Validation	anti-p-MLC validated by supplier; anti-GEF-H1 demonstrated in this study (also shown to have a little background); anti-tyrosinated-tubulin validated in this study with overexpression of tubulin detyrosinating enzymes; anti-α-tubulin validated in this study with Nocodazole experiment; anti-acetylated tubulin validated in this study with Tubacin treatment and tubulin acetylase overexpresion; anti-de-tyrosinated tubulin validated in this study by overexpression of de-tyrosinating enzyme complex VASH2+SVBP; anti-EB1+EB3 was verified in Komarova et al. Mol Biol Cell 2005.

Eukaryotic cell lines

Policy information about cell lines and Sex and Gender in Research

Cell line source(s)	HT1080 from J. Klumperman, UMC Utrecht (CVCL_0317); U2OS from ATCC (CVCL_0042); HEK293T from ATCC (CVCL_0063) ;Swiss 3T3 from ATCC (CVCL_0120); MDA-MB-231 from J.W.M. Martens, Erasmus MC Rotterdam (mCh-Lifeact) (CVCL_0062); 1205Lu from Wistar (Lifeact-eGFP) (CVCL_5239).
Authentication	Cell lines were not authenticated
Mycoplasma contamination	Cell lines were tested negative for mycoplasma contamination.
Commonly misidentified lines (See ICLAC register)	No commonly misidentified lines used.

Plants

Seed stocks	*Report on the source of all seed stocks or other plant material used. If applicable, state the seed stock centre and catalogue number. If plant specimens were collected from the field, describe the collection location, date and sampling procedures.*
Novel plant genotypes	*Describe the methods by which all novel plant genotypes were produced. This includes those generated by transgenic approaches, gene editing, chemical/radiation-based mutagenesis and hybridization. For transgenic lines, describe the transformation method, the number of independent lines analyzed and the generation upon which experiments were performed. For gene-edited lines, describe the editor used, the endogenous sequence targeted for editing, the targeting guide RNA sequence (if applicable) and how the editor was applied.*
Authentication	*Describe any authentication procedures for each seed stock used or novel genotype generated. Describe any experiments used to assess the effect of a mutation and, where applicable, how potential secondary effects (e.g. second site T-DNA insertions, mosiacism, off-target gene editing) were examined.*
