# Supplementary material for: Taxane-induced conformational changes in the microtubule lattice activate GEF-H1-dependent RhoA signalling
Source: Nat Cell Biol. 2026 Sep 24;28(11):2219–30. doi: 10.1038/s41556-026-02079-4 (PMC13751295; doi:10.1038/s41556-026-02079-4)

Unprocessed Blots - Meiring et al. 2026

Fig. 2g, h

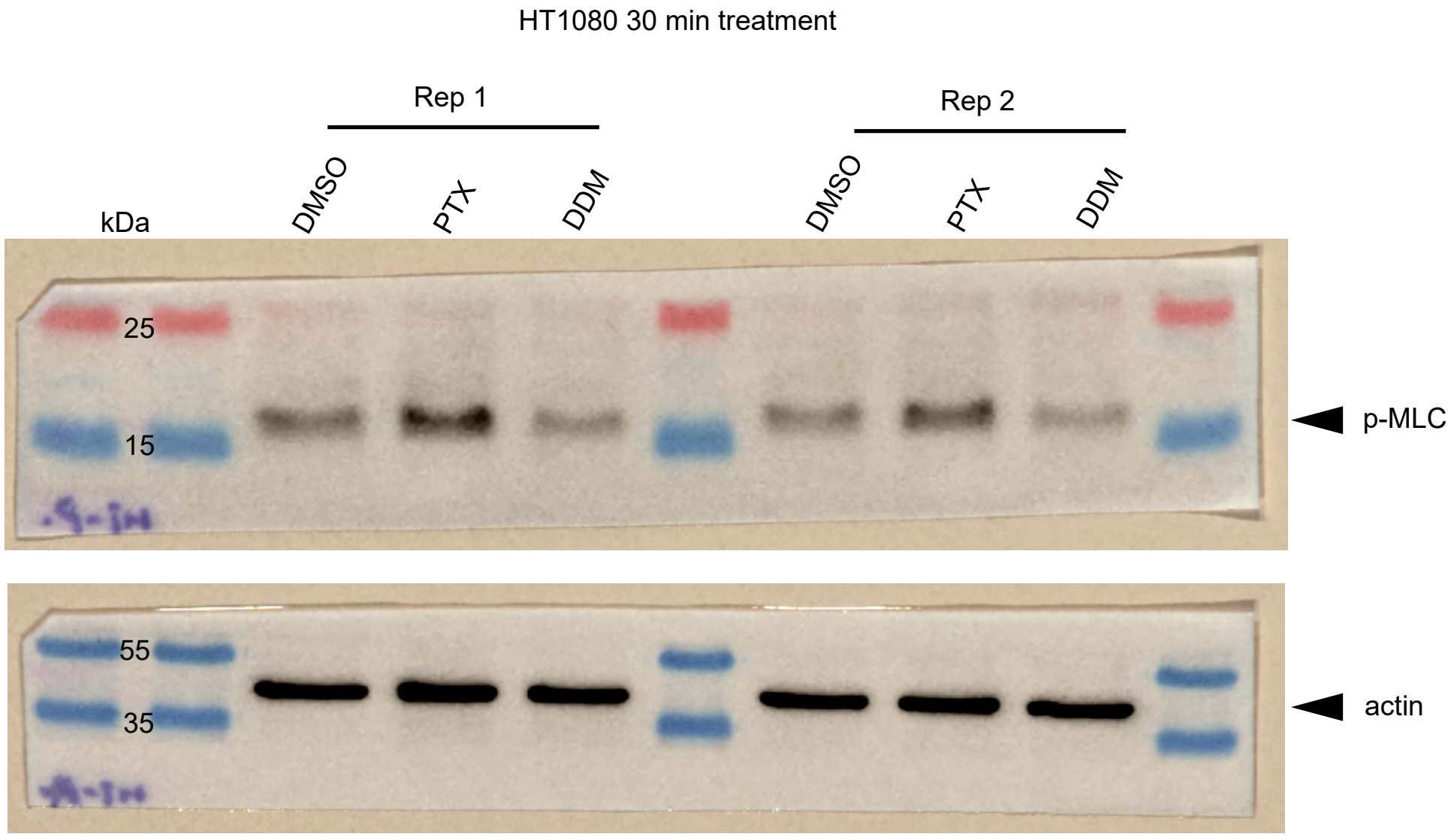

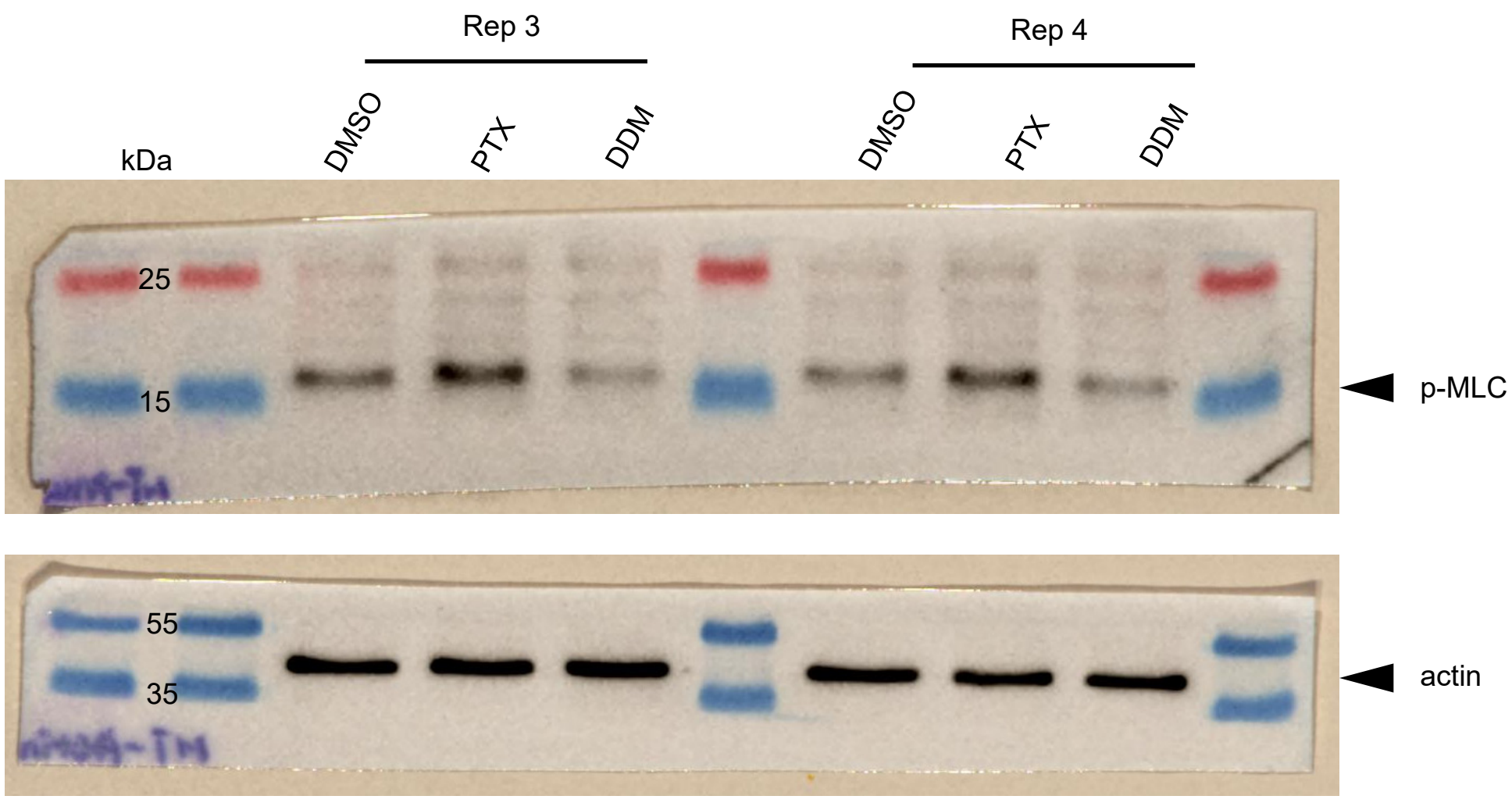

Unprocessed Blots - Meiring et al. 2026

Extended Data Fig. 1c

HT1080 WT and GEF-H1 KO

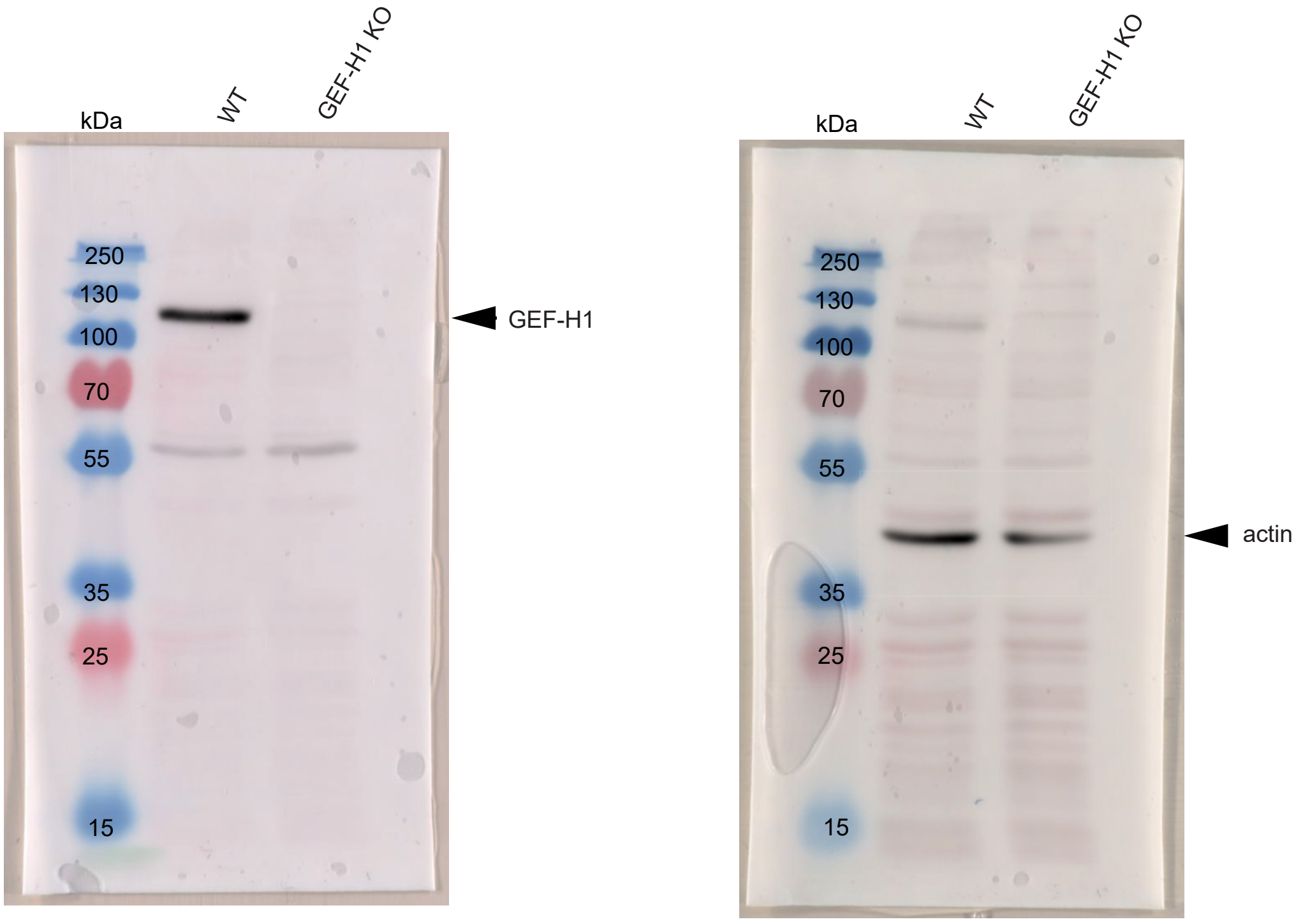

Unprocessed Blots - Meiring et al. 2026

Extended Data Fig. 2g, i

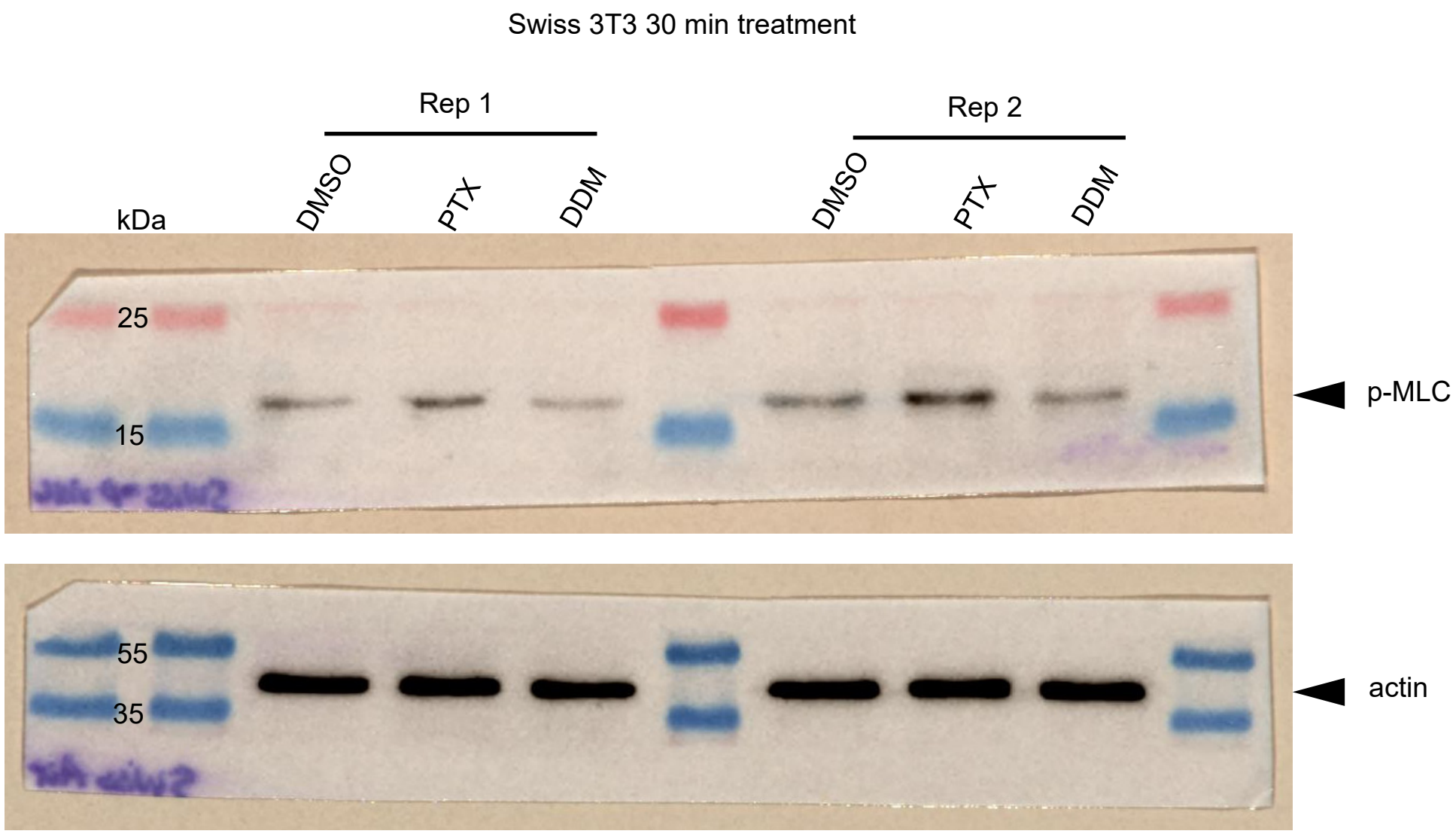

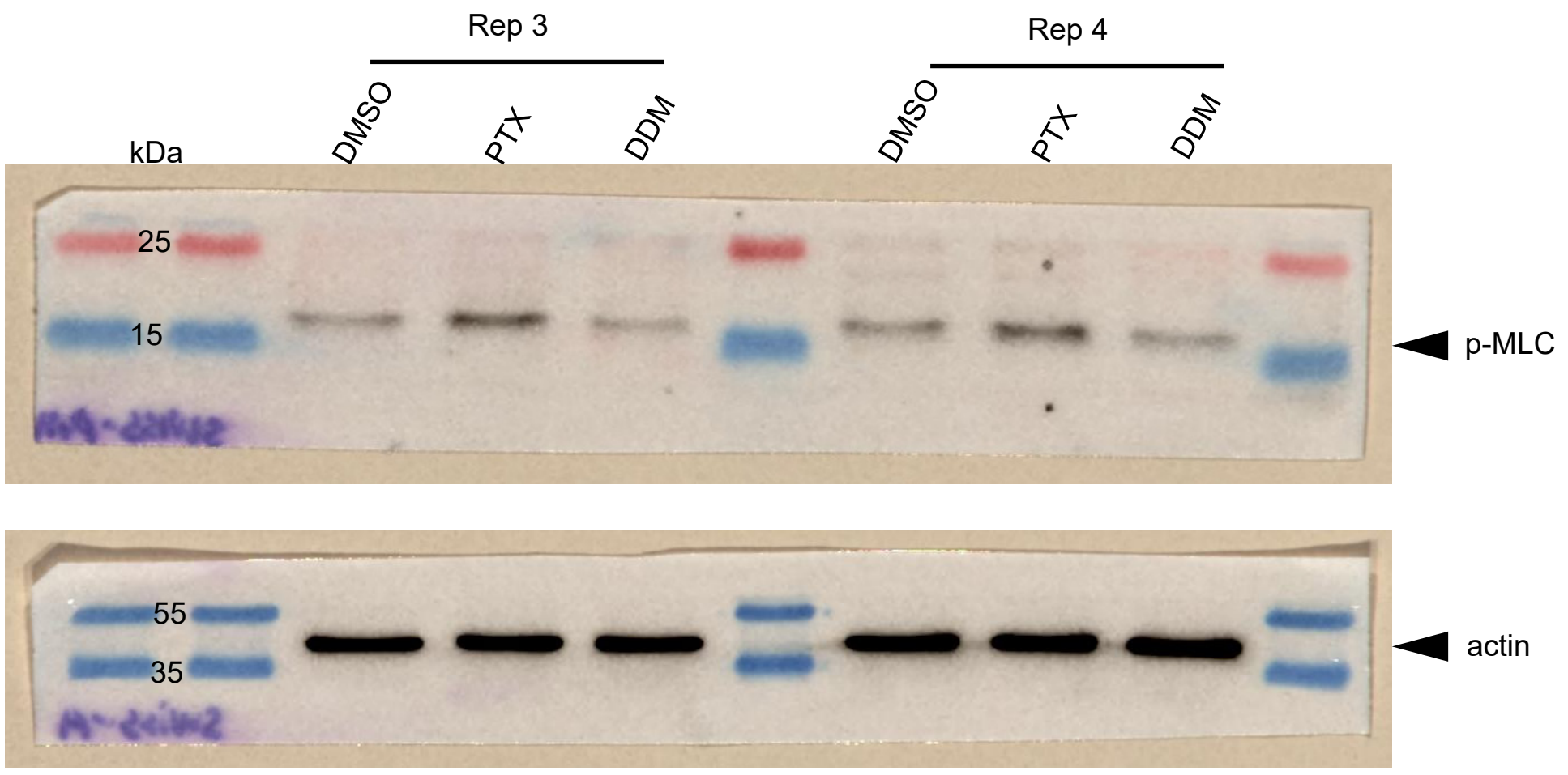

Unprocessed Blots - Meiring et al. 2026

Extended Data Fig. 2h, j

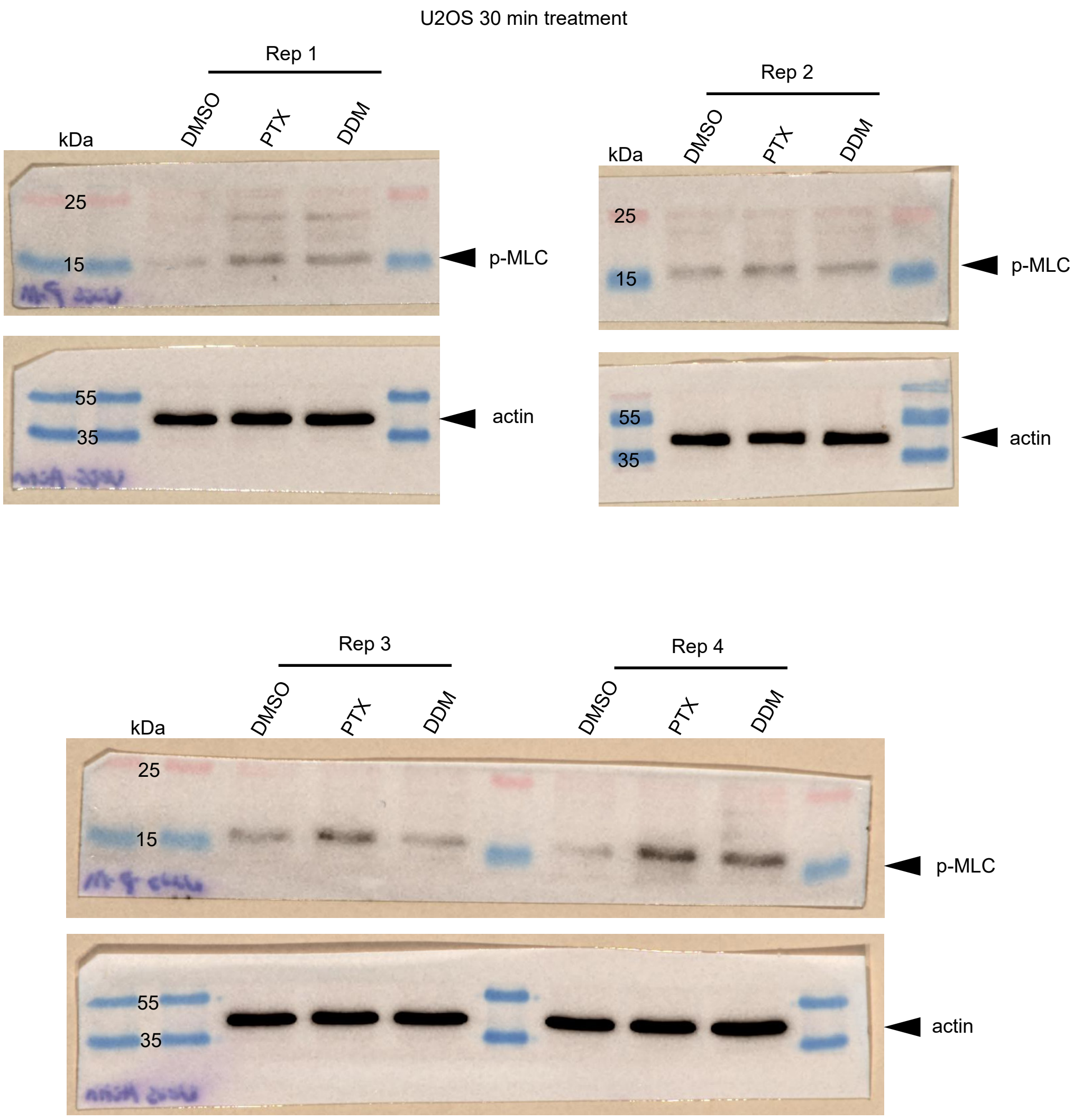

Supplement: Supplementary file 3 [file 41556_2026_2079_MOESM25_ESM.pdf]
