# Supplementary material for: Taxane-induced conformational changes in the microtubule lattice activate GEF-H1-dependent RhoA signalling
Source: Nat Cell Biol. 2026 Sep 24;28(11):2219–30. doi: 10.1038/s41556-026-02079-4 (PMC13751295; doi:10.1038/s41556-026-02079-4)
Supplement: Supplementary file 4 [file 41556_2026_2079_MOESM2_ESM.pdf]

nature portfolio

Peer Review File

Taxane-Induced Conformational Changes in the Microtubule Lattice Activate GEF-H1-Dependent RhoA Signaling.

Corresponding Author: Professor Anna Akhmanova

Version 0:

Decision Letter:

*Please delete the link to your author homepage if you wish to forward this email to co-authors.

Dear Professor Akhmanova,

Your manuscript, "Taxane-Induced Conformational Changes in the Microtubule Lattice Activate GEF-H1-Dependent RhoA Signaling.", has now been seen by 3 referees, who are experts in microtubule dynamics and structure (referee 1); microtubule dynamics and transport (referee 2); and MTOC and microtubule organization (referee 3). As you will see from their comments (attached below) they find this work of potential interest, but have raised substantial concerns, which in our view would need to be addressed with considerable revisions before we can consider publication in Nature Cell Biology.

Nature Cell Biology editors discuss the referee reports in detail within the editorial team, including the chief editor, to identify key referee points that should be addressed with priority, and requests that are overruled as being beyond the scope of the current study. To guide the scope of the revisions, I have listed these points below. We are committed to providing a fair and constructive peer-review process, so please feel free to contact me if you would like to discuss any of the referee comments further.

In particular, it would be essential to:

A) Provide additional experimental data regarding the autoinhibition mechanisms involving GEF-H1 (all Reviewers)

B) Address concerns about generalizable principles with additional experimental data in additional cell lines (Reviewers #2 and #3)

C) Provide additional data with live imaging to assess dynamics (Reviewer #1) and interaction with RhoA (all Reviewers).

D) All other referee concerns pertaining to strengthening existing data, providing controls, methodological details, clarifications and textual changes, should also be addressed.

E) Finally please pay close attention to our guidelines on statistical and methodological reporting (listed below) as failure to do so may delay the reconsideration of the revised manuscript. In particular please provide:

- a Supplementary Figure including unprocessed images of all gels/blots in the form of a multi-page pdf file. Please ensure that blots/gels are labeled and the sections presented in the figures are clearly indicated.

- a Supplementary Table including all numerical source data in Excel format, with data for different figures provided as different sheets within a single Excel file. The file should include source data giving rise to graphical representations and statistical descriptions in the paper and for all instances where the figures present representative experiments of multiple independent repeats, the source data of all repeats should be provided.

We would be happy to consider a revised manuscript that would satisfactorily address these points, unless a similar paper is published elsewhere, or is accepted for publication in Nature Cell Biology in the meantime.

When revising the manuscript please:

- ensure that it conforms to our format instructions and publication policies (see below and https://www.nature.com/nature/for-authors).

- provide a point-by-point rebuttal to the full referee reports verbatim, as provided at the end of this letter.

- for any revision that includes light microscopy data, we ask our authors to please include a completed light microscopy reporting table https://www.nature.com/documents/Light_microscopy_reporting_table.xlsx to ensure the methods are described thoroughly. The table will be available to reviewers and ultimately published should the manuscript be accepted at the journal.

- provide the completed Reporting Summary (found here https://www.nature.com/documents/nr-reporting-summary.pdf). This is essential for reconsideration of the manuscript will be available to editors and referees in the event of peer review. For more information see http://www.nature.com/authors/policies/availability.html or contact me.

When submitting the revised version of your manuscript, please pay close attention to our href="https://www.nature.com/nature-portfolio/editorial-policies/image-integrity">Digital Image Integrity Guidelines. and to the following points below:

-- that unprocessed scans are clearly labelled and match the gels and western blots presented in figures.
-- that control panels for gels and western blots are appropriately described as loading on sample processing controls
-- all images in the paper are checked for duplication of panels and for splicing of gel lanes.

Finally, please ensure that you retain unprocessed data and metadata files after publication, ideally archiving data in perpetuity, as these may be requested during the peer review and production process or after publication if any issues arise.

Nature Cell Biology is committed to improving transparency in authorship. As part of our efforts in this direction, we are now requesting that all authors identified as ‘corresponding author’ on published papers create and link their Open Researcher and Contributor Identifier (ORCID) with their account on the Manuscript Tracking System (MTS), prior to acceptance. ORCID helps the scientific community achieve unambiguous attribution of all scholarly contributions. You can create and link your ORCID from the home page of the MTS by clicking on ‘Modify my Springer Nature account’. For more information please visit please visit www.springernature.com/orcid.

This journal strongly supports public availability of data. Please place the data used in your paper into a public data repository, or alternatively, present the data as Supplementary Information. If data can only be shared on request, please explain why in your Data Availability Statement, and also in the correspondence with your editor. Please note that for some data types, deposition in a public repository is mandatory - more information on our data deposition policies and available repositories appears below.

Please submit the revised manuscript files and the point-by-point rebuttal to the referee comments using this link:

Link Redacted

*This url links to your confidential home page and associated information about manuscripts you may have submitted or be reviewing for us. If you wish to forward this email to co-authors, please delete the link to your homepage.

We would like to receive a revised submission within six months.

We hope that you will find our referees' comments, and editorial guidance helpful. Please do not hesitate to contact me if there is anything you would like to discuss.

Best wishes,

Daryl

Daryl Jason Verzosa David, PhD

Senior Editor, Nature Cell Biology
Advisory Editor, npj Biological Physics and Mechanics
Nature Portfolio

Heidelberger Platz 3, 14197 Berlin, Germany
Email: daryl.david@nature.com
ORCID: https://orcid.org/0000-0002-9253-4805

Reviewers' Comments:

Reviewer #1 (Remarks to the Author):

Dynamic microtubules regulate eukaryotic cell polarity, in part by influencing F-actin polymerization dynamics and retrograde flow. However, the signaling pathways linking microtubule dynamics to F-actin regulation remain poorly understood. Rho GTPases, activated by guanine nucleotide exchange factors (GEFs) such as GEF-H1 (a Rho-specific GEF), modulate the actin cytoskeleton and myosin-driven contractility.
In this manuscript, Akhmanova and colleagues investigate how the microtubule-stabilizing drug Taxol (paclitaxel) and its derivatives affect GEF-H1 binding to microtubules, Rho activation, and subsequent F-actin cytoskeleton organization. The authors show that Taxol binding induces GEF-H1 dissociation from microtubules (likely via allosteric effects on the microtubule lattice), which potentially activates GEF-H1, alters F-actin organization, and changes cell shape. They support this with in vitro reconstitution assays demonstrating Taxol's impact on GEF-H1 localization, combined with in vivo cell biology experiments (primarily using fixed cells or fluorescently tagged actin/microtubules).
The manuscript is of interest to wide audiences, but the authors must revise the manuscript to address the experimental and conceptual concerns:

Strengths
1)The study integrates in vitro and in vivo approaches to address how microtubule stabilization by Taxol releases GEF-H1.
2)It provides novel insights into an unexpected mechanism: Taxol-induced microtubule lattice changes can displace GEF-H1, contrasting with the more typical activation by microtubule depolymerization.
3)The topic is broadly relevant to cytoskeletal regulation, Rho signaling, and the cellular effects of microtubule-targeting drugs used in cancer therapy.

Major Concerns
1)The manuscript focuses narrowly on the mechanistic effect of Taxol on GEF-H1 release but does not sufficiently address key biological questions, such as whether GEF-H1 release leads to its activation, and the downstream implications for cell motility (e.g., in fibroblasts) or long-term processes like cell division.
2)Cell biology data rely heavily on fixed samples or static imaging of fluorescently tagged actin/microtubules; this limits insights into dynamic processes.
3)The study equates GEF-H1 binding to microtubules with inactivation but does not explore the multi-domain nature of GEF-H1 or potential autoinhibitory mechanisms beyond simple sequestration.
4)The discussion largely overlooks the native biological role of microtubule-associated GEF-H1 autoinhibition in regulating F-actin dynamics and cell motility. It does not adequately discuss physiological signals (e.g., native substrates or microtubule depolymerization) that release GEF-H1 in normal cells, nor does it frame Taxol's effects in the context of perturbing a native pathway. Instead, the discussion emphasizes Taxol's cancerous/cellular effects without sufficient grounding in endogenous regulation.
Specific Recommendations for Revision
1.Enhance biological impact studies with live cell imaging of motile and dividing cells:
A)Perform live-cell imaging to assess the effects of Taxol on cell motility (e.g., migration assays in fibroblasts) and cell division.
B)Conduct additional live imaging to directly examine the dynamics of f-actin mediated Rho activation downstream of GEF-H1 release from microtubules.

2.Investigate GEF-H1 autoinhibition mechanism:
A) Explore structural homology models comparing GEF-H1 to other GEFs, focusing on how microtubule binding inhibits GEF activity and how release induces activation.
B) Test mutants that disrupt putative autoinhibitory domains or the microtubule-binding site to determine whether microtubule-bound GEF-H1 can be active and how this affects the F-actin cytoskeleton in cells.
3.Revise and expand the discussion:
A)Prioritize the native biological pathway: Discuss the physiological role of coupling GEF-H1 auto-inhibition to microtubules in regulating F-actin dynamics and key processes like cell motility.
B)Address open questions: Are there endogenous signals/substrates that release GEF-H1 from microtubules? Is microtubule depolymerization the primary physiological trigger for GEF-H1 activation?
C)Reposition Taxol effects as perturbation of this native pathway, rather than treating them as the primary focus.

These revisions would strengthen the manuscript's conceptual depth, broaden its biological relevance, and better justify its interest to a wide audience. The work has potential but requires these revisions to fully resolve experimental and conceptual gaps.

Reviewer #2 (Remarks to the Author):

The manuscript by Meiring et al addresses a long-standing question in cell biology (how is RhoA activated by changes in microtubules) by studying an emerging idea in the microtubule field (that the conformation of tubulin subunits within the microtubule lattice is locally written and read as part of the tubulin code). Over 20 years ago, it was shown that microtubule-destabilizing agents activate RhoA by releasing GEF-H1 from microtubules. With the emerging ideas in the microtubule

field, Meiring et al asked whether conformational changes in tubulin subunits in the lattice can also release GEF-H1 and activate RhoA. The authors find that treating cells with paclitaxel, with causes tubulin to adopt an extended conformation, causes activation of RhoA signaling and release of GEF-H1 from microtubules and that the effects are dependent on GEF-H1. They then reconstitute GEF-H1 binding to microtubules and show a preference for the compacted state. This is a really strong paper, the results are exciting, and the information is timely although a few control experiments and some text revisions would strengthen and clarify the manuscript.

Major comments:

1) Why HT1080 fibrosarcoma cells? It would strengthen the findings if the authors could repeat key experiments in cells commonly used to investigate Rho signaling such as mouse embryonic fibroblasts, U2OS, or NIH3T3 cells. Especially since GEF-H1 KO cells have higher FBS response, there may be quite a bit of variability between cell lines and it would be good to see whether the effects are reproducible. Maybe it's too much with the Rho FRET sensor but at least repeating experiments with the stress fibers and GEF-H1 release in another cell line.

2) I don't understand the FChitax-3 experiments. The impact on RhoA activation is not measured directly and these experiments were done with a different drug in different cell line at different conc than paclitaxel and require a much longer response time. Also, according to Lucena-Agell et al (2025), FChitax3 treatment should result in a compacted microtubule lattice.

3) Since one of the points the authors want to make is that discodermolide is a lattice compactor that can be used as a control for paclitaxel treatment, the authors need to show that discodermolide does not activate RhoA. The FChitax3, if needed, could be moved to the supplement.

4) Likewise, it would be good to show that discodermolide does not cause stabilization of filamentous actin and increased actomyosin contractility. Also, for Fig 2, FBS would be a better control since it activates RhoA signaling without GEF-H1.

5) For Fig 3, the authors rely on overexpression studies and state that they image cells at low levels, but no information or quantification is provided for how much overexpression there is, even for low levels of expression. For such important experiments, I am surprised the authors did not make a knockin cell line so they can look at GEF-H1 under endogenous conditions. At a minimum, they should show the effects of various treatments on GEF-H1 localization at different levels of expression. It seems likely that at high levels of expression, GEF-H1 can resist paclitaxel, perhaps by compacting the lattice (e.g. cell in top left corner of Fig 3F). The data in Fig 4P also suggest that GEF-H1 can compact a GMPCPP seed. Can the authors delve into this more fully, for example using experiments similar to Paquette et al 2025 where they vary the concentrations of GEF-H1 and paclitaxel.

5) One point the authors want to make is that GEF-H1 avoids acetylated microtubules. Their results do support the hypothesis that GEF-H1 binding to microtubules is not dependent on PTMs but the relationship between PTM state and conformational state is unclear. Recent work in the field suggests that the tubulin expanded conformation can drive PTM (e.g. Yue et al 2023, Yue et al 2025, Egoldt et al 2025) but it is not clear whether overexpression of PTM enzymes and/or the modification drives tubulin to an expanded conformation. Indeed, if they overexpress alphaTAT to increase acetylation, then GEF-H1 is on all the microtubules, suggesting that acetylated microtubules could be compacted.

6) To fully support the hypothesis that the expanded tubulin state in cells releases GEF-H1, the authors should utilize methods that other papers have recently used such as stableMARK or a MAP or osmotic shock.

7) The reconstitution experiments suggest that GEF-H1 (full length and truncation) prefers GTPgS > GDP > GMPCPP microtubules. Can the authors quantify this? If so, then this is consistent with GEF-H1 preferring a compacted lattice state. But then why doesn't GEF-H1 track plus ends in cells and in vitro? Note that the authors use GTPgS as a GTP analog, but recent work indicates that GTPgS is a compacted lattice.

8) It is surprising that paclitaxel knocks both EB and GEF-H1 off of the lattice whereas discodermolide knocks off EB but not GEF-H1. Both EB and GEF-H1 prefer to bind to a GTPgS lattice and the dimer rise of a discodermolide lattice is similar to that of GTPgS lattice (Fig S4).

10) The authors state that discodermolide generates a compacted lattice based on cryoEM but there is no data backing up this claim. For Fig S4, the authors should state how they arrived at each point on the graphs (i.e. they need references).

Minor comments:

1) Out of curiosity, are GEF-H1 mutations associated with cancer and or taxol resistance?

2) The authors state that paclitaxel expands lattice and reference Kellogg et al 2017. But the Kellogg et al manuscript states that paclitaxel does not expand the lattice if added after polymerization.

3) The authors show that activation of RhoA via FBS is not dependent on GEF-H1. What is known about how FBS does activate RhoA signaling? This would help the reader interpret the results.

4) For Fig 1, what are the authors' thoughts on needing such high levels of paclitaxel if clinical levels are nM and they see response at uM levels

5) For Fig 2, the authors use actin as gel loading control but they are measuring changes in actin fluorescence intensity in response to the treatments. It looks in the gel that actin changes in response to paclitaxel (like in the intensity measurements) so a different loading control for the western blot is needed.

6) Can the authors comment on the differences between nocodazole and paclitaxel in terms of RhoA in Fig 1A vs actin and circularity in Fig 2? The treatments in Fig 1A result in very different RhoA activation profiles in terms of time of increase and whether the signal decays over time. At 30 min, RhoA is still increasing in nocodazole treatment where it has almost returned to baseline in paclitaxel. Yet both treatments give the same response in Fig 2. If a short Rho activation is sufficient, then stimulating with FBS should give the same results in Fig 2.

7) For the graphs with individual data plotted, the different shapes and colors are not needed and the yellow x is very difficult to see. It would be better to use black spots for every plot and then a bright color for the averages. Are the statistics done comparing all the spots or the averages? Also, please show 95% confidence intervals rather than SD.

8) For the experiments where they overexpress detyrosination enzymes (Fig S2), it would be better to stain for total tubulin (something that is not changing) instead of Tyr-tubulin (which is decreasing).

9) In Fig 3G, the images do not match up.

10) What are red and green arrows in Fig 4A?

Reviewer #3 (Remarks to the Author):

The study by Meiring et al. investigates how effects of different microtubule-targeted drugs are linked to activation of RhoA-dependent signalling. This is a highly relevant question in the context of cancer chemotherapy where the therapeutic effects of such drugs are still incompletely understood. There is strong evidence that interference with mitotic spindle assembly and function is not the only therapeutically relevant effect, and that additional non-mitotic effects such as activation of Rho-dependent pathways play a role. This could modulate cancer cell survival through reduced adhesion, immune cell function, or blood vessel sprouting.

Based on previous work showing that sequestration of the RhoA activator GEF-H1 through binding to microtubule lattices inhibits Rho signalling, the authors show that, somewhat counter-intuitively, not only depolymerization of microtubules, but also stabilization by taxanes results in GEF-H1 release and Rho pathway activation. The authors then reveal the molecular basis of this effect, showing that GEF-H1 recognizes tubulin spacing in the microtubule lattice, which is increased through microtubule lattice expansion by taxanes, leading to GEF-H1 release, despite the stabilization and presence of microtubule lattices. For this they use cell-based assays to demonstrate Rho pathway activation and GEF-H1 microtubule interaction and also reconstitute the effects in vitro using TIRF imaging of GEF-H1 interaction with compacted and expanded microtubule lattices obtained with various microtubule binding compounds. Finally, as additional control for their model, they use cryo-EM and structural analyses to determine that discodermolide, while also stabilizing microtubules, compacts lattices and does not trigger GEF-H1 release.

This very well-executed and presented study closes an important gap in our understanding of the cellular effects of microtubule-targeted drugs used in chemotherapy. It highlights the differential effects of these drugs on activation of Rho signalling, which may be an additional therapeutic benefit, opening up new routes for further investigation and drug development in the future. Apart from this, the work also establishes microtubule lattice conformation as an important regulatory mechanism that, together with posttranslational modifications, could be part of the tubulin "code" that is read by microtubule interacting proteins.

This is a very nice paper, presenting high quality data to support the conclusions. I have found only one issue that needs to be addressed:

There is a disconnect between cellular data in the first part on Rho pathway activation and contractility (Fig. 1 and 2) and the second part on the underlying mechanism analysing microtubule conformations and GEF-H1 binding to microtubules (Fig. 3). The disconnect stems from the switch from HT1080 cells in the first part to U2OS in the second part. The authors justify this with better microtubule imaging in U2OS due to their stronger adherence and flattened morphology. But this also means that there is no demonstrated effect on Rho activation in these cells (e.g. no cell rounding, increased contractility). Similarly, in the experiments with HT1080 cells, no GEF-H1-microtubule binding/release is shown. Thus, the current data are based on correlation of two different effects obtained independently in two different cell lines (Rho pathway signalling vs. lattice state and GEF-H1 interaction). I strongly suggest that the authors demonstrate a direct effect in the same cell line of lattice spacing induced by a specific drug on GEF-H1 binding state, and on Rho-dependent signaling/contractility. This could be done by analyzing microtubules in HT1080, perhaps using an adherence-promoting substrate, or by analyzing Rho activity and contractility in U2OS cells. Alternatively, another suitable cell line could be used. Without such data, the support for the main message of this paper would be significantly weaker.

GUIDELINES FOR MANUSCRIPT SUBMISSION TO NATURE CELL BIOLOGY

READABILITY OF MANUSCRIPTS – Nature Cell Biology is read by cell biologists from diverse backgrounds, many of whom are not native English speakers. Authors should aim to communicate their findings clearly, explaining technical jargon that might be unfamiliar to non-specialists, and avoiding non-standard abbreviations. Titles and abstracts should concisely communicate the main findings of the study, and the background, rationale, results and conclusions should be clearly explained in the manuscript in a manner accessible to a broad cell biology audience. Nature Cell Biology uses British spelling.

MANUSCRIPT FORMAT – please follow the guidelines listed in our Guide to Authors regarding manuscript formats at Nature Cell Biology.

TITLE – should be no more than 100 characters including spaces, without punctuation and avoiding technical terms, abbreviations, and active verbs..

AUTHOR NAMES – should be given in full.

AUTHOR AFFILIATIONS – should be denoted with numerical superscripts (not symbols) preceding the names. Full addresses should be included, with US states in full and providing zip/post codes. The corresponding author is denoted by: "Correspondence should be addressed to [initials]."

ABSTRACT AND MAIN TEXT – please follow the guidelines that are specific to the format of your manuscript, as listed in our Guide to Authors (http://www.nature.com/ncb/pdf/ncb_gta.pdf) Briefly, Nature Cell Biology Articles, Resources and Technical Reports have 3500 words, including a 150 word abstract, and the main text is subdivided in Introduction, Results, and Discussion sections. Nature Cell Biology Letters have up to 2500 words, including a 180 word introductory paragraph (abstract), and the text is not subdivided in sections.

ACKNOWLEDGEMENTS – should be kept brief. Professional titles and affiliations are unnecessary. Grant numbers can be listed.

AUTHOR CONTRIBUTIONS – must be included after the Acknowledgements, detailing the contributions of each author to the paper (e.g. experimental work, project planning, data analysis etc.). Each author should be listed by his/her initials.

FINANCIAL AND NON-FINANCIAL COMPETING INTERESTS – the authors must include one of three declarations: (1) that they have no financial and non-financial competing interests; (2) that they have financial and non-financial competing interests; or (3) that they decline to respond, after the Author Contributions section. This statement will be published with the article, and in cases where financial and non-financial competing interests are declared, these will be itemized in a web supplement to the article. For further details please see https://www.nature.com/licenceforms/nrg/competing-interests.pdf.

REFERENCES – are limited to a total of 70 for Articles, Resources, Technical Reports; and 40 for Letters. This includes references in the main text and Methods combined. References must be numbered sequentially as they appear in the main text, tables and figure legends and Methods and must follow the precise style of Nature Cell Biology references. References only cited in the Methods should be numbered consecutively following the last reference cited in the main text. References only associated with Supplementary Information (e.g. in supplementary legends) do not count toward the total reference limit and do not need to be cited in numerical continuity with references in the main text. Only published papers can be cited, and each publication cited should be included in the numbered reference list, which should include the manuscript titles. Footnotes are not permitted.

METHODS – Nature Cell Biology publishes methods online. The methods section should be provided as a separate Word document, which will be copyedited and appended to the manuscript PDF, and incorporated within the HTML format of the paper.

Methods should be written concisely, but should contain all elements necessary to allow interpretation and replication of the results. As a guideline, Methods sections typically do not exceed 3,000 words. The Methods should be divided into subsections listing reagents and techniques. When citing previous methods, accurate references should be provided and any alterations should be noted. Information must be provided about: antibody dilutions, company names, catalogue numbers and clone numbers for monoclonal antibodies; sequences of RNAi and cDNA probes/primers or company names and catalogue numbers if reagents are commercial; cell line names, sources and information on cell line identity and authentication. Animal studies and experiments involving human subjects must be reported in detail, identifying the committees approving the protocols. For studies involving human subjects/samples, a statement must be included confirming that informed consent was obtained. Statistical analyses and information on the reproducibility of experimental

results should be provided in a section titled “Statistics and Reproducibility”.

All Nature Cell Biology manuscripts submitted on or after March 21 2016 must include a Data availability statement as a separate section after Methods but before references, under the heading "Data Availability". . For Springer Nature policies on data availability see http://www.nature.com/authors/policies/availability.html; for more information on this particular policy see http://www.nature.com/authors/policies/data/data-availability-statements-data-citations.pdf. The Data availability statement should include:

• Accession codes for primary datasets (generated during the study under consideration and designated as "primary accessions") and secondary datasets (published datasets reanalysed during the study under consideration, designated as "referenced accessions"). For primary accessions data should be made public to coincide with publication of the manuscript. A list of data types for which submission to community-endorsed public repositories is mandated (including sequence, structure, microarray, deep sequencing data) can be found here http://www.nature.com/authors/policies/availability.html#data.

• Unique identifiers (accession codes, DOIs or other unique persistent identifier) and hyperlinks for datasets deposited in an approved repository, but for which data deposition is not mandated (see here for details http://www.nature.com/sdata/data-policies/repositories).

• At a minimum, please include a statement confirming that all relevant data are available from the authors, and/or are included with the manuscript (e.g. as source data or supplementary information), listing which data are included (e.g. by figure panels and data types) and mentioning any restrictions on availability.

• If a dataset has a Digital Object Identifier (DOI) as its unique identifier, we strongly encourage including this in the Reference list and citing the dataset in the Methods.

We recommend that you upload the step-by-step protocols used in this manuscript to protocols.io. More details can found at https://www.protocols.io/help/publish-articles.

DISPLAY ITEMS – main display items are limited to 6-8 main figures and/or main tables for Articles, Resources, Technical Reports; and 5 main figures and/or main tables for Letters. For Supplementary Information see below.

FIGURES – Colour figure publication costs $600 for the first, and $300 for each subsequent colour figure. All panels of a multi-panel figure must be logically connected and arranged as they would appear in the final version. Unnecessary figures and figure panels should be avoided (e.g. data presented in small tables could be stated briefly in the text instead).

All imaging data should be accompanied by scale bars, which should be defined in the legend.
Cropped images of gels/blots are acceptable, but need to be accompanied by size markers, and to retain visible background signal within the linear range (i.e. should not be saturated). The boundaries of panels with low background have to be demarked with black lines. Splicing of panels should only be considered if unavoidable, and must be clearly marked on the figure, and noted in the legend with a statement on whether the samples were obtained and processed simultaneously. Quantitative comparisons between samples on different gels/blots are discouraged; if this is unavoidable, it should only be performed for samples derived from the same experiment with gels/blots were processed in parallel, which needs to be stated in the legend.

Figures should be provided at approximately the size that they are to be printed at (single column is 86 mm, double column is 170 mm) and should not exceed an A4 page (8.5 x 11"). Reduction to the scale that will be used on the page is not necessary, but multi-panel figures should be sized so that the whole figure can be reduced by the same amount at the smallest size at which essential details in each panel are visible. In the interest of our colour-blind readers we ask that you avoid using red and green for contrast in figures. Replacing red with magenta and green with turquoise are two possible colour-safe alternatives. Lines with widths of less than 1 point should be avoided. Sans serif typefaces, such as Helvetica (preferred) or Arial should be used. All text that forms part of a figure should be rewritable and removable.

We accept files from the following graphics packages in either PC or Macintosh format:

- For line art, graphs, charts and schematics we prefer Adobe Illustrator (.AI), Encapsulated PostScript (.EPS) or Portable Document Format (.PDF). Files should be saved or exported as such directly from the application in which they were made, to allow us to restyle them according to our journal house style.

- We accept PowerPoint (.PPT) files if they are fully editable. However, please refrain from adding PowerPoint graphical effects to objects, as this results in them outputting poor quality raster art. Text used for PowerPoint figures should be Helvetica (preferred) or Arial.

- We do not recommend using Adobe Photoshop for designing figures, but we can accept Photoshop generated (.PSD or .TIFF) files only if each element included in the figure (text, labels, pictures, graphs, arrows and scale bars) are on separate layers. All text should be editable in ‘type layers’ and line-art such as graphs and other simple schematics should be preserved and embedded within 'vector smart objects’ - not flattened raster/bitmap graphics.

- Some programs can generate Postscript by 'printing to file' (found in the Print dialogue). If using an application not listed above, save the file in PostScript format or email our Art Editor, Allen Beattie for advice (a.beattie@nature.com).

Regardless of format, all figures must be vector graphic compatible files, not supplied in a flattened raster/bitmap graphics format, but should be fully editable, allowing us to highlight/copy/paste all text and move individual parts of the figures (i.e. arrows, lines, x and y axes, graphs, tick marks, scale bars etc.). The only parts of the figure that should be in pixel raster/bitmap format are photographic images or 3D rendered graphics/complex technical illustrations.

All placed images (i.e. a photo incorporated into a figure) should be on a separate layer and independent from any superimposed scale bars or text. Individual photographic images must be a minimum of 300+ DPI (at actual size) or kept constant from the original picture acquisition and not decreased in resolution post image acquisition. All colour artwork should be RGB format.

EXTENDED DATA FIGURES - When re-submitting your manuscript, please ensure that any supplementary figures and tables that are crucial to the manuscript's conclusions are converted into Extended Data figures and tables to increase visibility of these data. Extended Data figures and tables are online-only (present in the online PDF and full-text HTML versions of the paper), peer-reviewed display items that provide essential background to the article but are not included in the main article due to space constraints. A maximum of ten Extended Data display items (figures and tables) is permitted.

FIGURE LEGENDS – must not exceed 350 words for each figure to allow fit on a single printed NCB page together with the figure. They must include a brief title for the whole figure, and short descriptions of each panel with definitions of the symbols used, but without detailing methodology.

TABLES – main tables should be provided as individual Word files, together with a brief title and legend. For supplementary tables see below.

SUPPLEMENTARY INFORMATION – Supplementary information is material directly relevant to the conclusion of a paper, but which cannot be included in the printed version in order to keep the manuscript concise and accessible to the general reader. Supplementary information is an integral part of a Nature Cell Biology publication and should be prepared and presented with as much care as the main display item, but it must not include non-essential data or text, which may be removed at the editor's discretion. All supplementary material is fully peer-reviewed and published online as part of the HTML version of the manuscript. Supplementary Figures and Supplementary Notes are appended at the end of the main PDF of the published manuscript.

Supplementary items should relate to a main text figure, wherever possible, and should be mentioned sequentially in the main manuscript, designated as Supplementary Figure, Table, Video, or Note, and numbered continuously (e.g. Supplementary Figure 1, Supplementary Figure 2, Supplementary Table 1, Supplementary Table 2 etc.).

Unprocessed scans of all key data generated through electrophoretic separation techniques need to be presented in a supplementary figure that should be labelled and numbered as the final supplementary figure, and should be mentioned in every relevant figure legend. This figure does not count towards the total number of figures and is the only figure that can be displayed over multiple pages, but should be provided as a single file, in PDF or TIFF format. Data in this figure can be displayed in a relatively informal style, but size markers and the figures panels corresponding to the presented data must be indicated.

The total number of Supplementary Figures (not including the "unprocessed scans" Supplementary Figure) should not exceed the number of main display items (figures and/or tables (see our Guide to Authors and March 2012 editorial http://www.nature.com/ncb/authors/submit/index.html#suppinfo; http://www.nature.com/ncb/journal/v14/n3/index.html#ed). No restrictions apply to Supplementary Tables or Videos, but we advise authors to be selective in including supplemental data.

Each Supplementary Figure should be provided as a single page and as an individual file in one of our accepted figure formats and should be presented according to our figure guidelines (see above). Supplementary Tables should be provided as individual Excel files. Supplementary Videos should be provided as .avi or .mov files up to 50 MB in size. Supplementary Figures, Tables and Videos much be accompanied by a separate Word document including titles and legends.

GUIDELINES FOR EXPERIMENTAL AND STATISTICAL REPORTING

REPORTING REQUIREMENTS – We are trying to improve the quality of methods and statistics reporting in our papers. To that end, we are now asking authors to complete a reporting summary that collects information on experimental design and reagents. The Reporting Summary can be found here https://www.nature.com/documents/nr-reporting-summary.pdf)If you would like to reference the guidance text as you complete the template, please access these flattened versions at http://www.nature.com/authors/policies/availability.html.

STATISTICS – Wherever statistics have been derived the legend needs to provide the n number (i.e. the sample size used to derive statistics) as a precise value (not a range), and define what this value represents. Error bars need to be defined in the legends (e.g. SD, SEM) together with a measure of centre (e.g. mean, median). Box plots need to be defined in terms of

minima, maxima, centre, and percentiles. Ranges are more appropriate than standard errors for small data sets. Wherever statistical significance has been derived, precise p values need to be provided and the statistical test used needs to be stated in the legend. Statistics such as error bars must not be derived from n<3. For sample sizes of n<5 please plot the individual data points rather than providing bar graphs. Deriving statistics from technical replicate samples, rather than biological replicates is strongly discouraged. Wherever statistical significance has been derived, precise p values need to be provided and the statistical test stated in the legend.

Information on how many times each experiment was repeated independently with similar results needs to be provided in the legends and/or Methods for all experiments, and in particular wherever representative experiments are shown.

We strongly recommend the presentation of source data for graphical and statistical analyses as a separate Supplementary Table, and request that source data for all independent repeats are provided when representative experiments of multiple independent repeats, or averages of two independent experiments are presented. This supplementary table should be in Excel format, with data for different figures provided as different sheets within a single Excel file. It should be labelled and numbered as one of the supplementary tables, titled “Statistics Source Data”, and mentioned in all relevant figure legends.

--------- Please don't hesitate to contact NCB@nature.com should you have queries about any of the above requirements ---------

Version 1:

Decision Letter:

Our ref: NCB-A61065A

20th July 2026

Dear Dr. Akhmanova,

Thank you for submitting your revised manuscript "Taxane-Induced Conformational Changes in the Microtubule Lattice Activate GEF-H1-Dependent RhoA Signaling." (NCB-A61065A). It has now been seen by the original referees and their comments are below. The reviewers find that the paper has improved in revision, and therefore we'll be happy in principle to publish it in Nature Cell Biology, pending minor revisions to satisfy the referees' final requests and to comply with our editorial and formatting guidelines.

The current version of your manuscript is in a PDF format. Therefore, please email us a copy of the file in an editable format (Microsoft Word or LaTex)-- we can not proceed with PDFs at this stage.

We are now performing detailed checks on your paper and will send you a checklist detailing our editorial and formatting requirements in a couple of weeks. Please do not upload the final materials and make any revisions until you receive this additional information from us.

Thank you again for your interest in Nature Cell Biology. Please do not hesitate to contact me if you have any questions.

Sincerely,
Daryl

Daryl Jason Verzosa David, PhD

Senior Editor, Nature Cell Biology
Advisory Editor, npj Biological Physics and Mechanics
Nature Portfolio

Heidelberger Platz 3, 14197 Berlin, Germany
Email: daryl.david@nature.com
ORCID: https://orcid.org/0000-0002-9253-4805

Reviewer #1 (Remarks to the Author):

The authors have addressed all the concerns raised by the reviewers in the revised manuscript.

Reviewer #2 (Remarks to the Author):

The revised manuscript by Meiring et al has added new text and experimental evidence that clarify and/or support their

findings. The main point is that the sequestraiton of GEF-H1, the major RhoA activitor, on microtubules is regulated by the conformational state of the microtubule. This finding provides a mechanism for the fact that both microtubule depolymerization and stabilization activate RhoA. The experiments are well-done and compelling, the findings are exciting and foundational, and the work is timely and important.

Reviewer #3 (Remarks to the Author):

The authors have done a very thorough revision. The issue that I raised was addressed and I have no further concerns.

INTERNAL USE ONLY AIP modules required: ["Clinical","Life Science"]

Version 2:

Decision Letter:

Dear Dr Akhmanova,

I am pleased to inform you that your manuscript, "Taxane-Induced Conformational Changes in the Microtubule Lattice Activate GEF-H1-Dependent RhoA Signaling.", has now been accepted for publication in Nature Cell Biology.

Thank you for sending us the final manuscript files to be processed for print and online production, and for returning the manuscript checklists and other forms. Your manuscript will now be passed to our production team who will be in contact with you if there are any questions with the production quality of supplied figures and text.

Over the next few weeks, your paper will be copyedited to ensure that it conforms to Nature Cell Biology style. Once your paper is typeset, you will receive an email with a link to choose the appropriate publishing options for your paper and our Author Services team will be in touch regarding any additional information that may be required.

After the grant of rights is completed, you will receive a link to your electronic proof via email with a request to make any corrections within 48 hours. If, when you receive your proof, you cannot meet this deadline, please inform us at rjsproduction@springernature.com immediately.

You will not receive your proofs until the publishing agreement has been received through our system.

Due to the importance of these deadlines, we ask that you please let us know now whether you will be difficult to contact over the next month. If this is the case, we ask you provide us with the contact information (email, phone and fax) of someone who will be able to check the proofs on your behalf, and who will be available to address any last-minute problems.

If you have any questions about our publishing options, costs, Open Access requirements, or our legal forms, please contact ASJournals@springernature.com

Once your paper has been scheduled for online publication, the Nature press office will be in touch to confirm the details. An online order form for reprints of your paper is available at https://www.nature.com/reprints/author-reprints.html. All co-authors, authors' institutions and authors' funding agencies can order reprints using the form appropriate to their geographical region.

Publication is conditional on the manuscript not being published elsewhere and on there being no announcement of this work to any media outlet until the online publication date in Nature Cell Biology.

You may wish to make your media relations office aware of your accepted publication, in case they consider it appropriate to organize some internal or external publicity. Once your paper has been scheduled you will receive an email confirming the publication details. This is normally 3-4 working days in advance of publication. If you need additional notice of the date and time of publication, please let the production team know when you receive the proof of your article to ensure there is sufficient time to coordinate. Further information on our embargo policies can be found here: https://www.nature.com/authors/policies/embargo.html

Please note that *Nature Cell Biology* is a Transformative Journal (TJ). Authors may publish their research with us through the traditional subscription access route or make their paper immediately open access through payment of an article-processing charge (APC). Authors will not be required to make a final decision about access to their article until it has been accepted. Find out more about Transformative Journals

Authors may need to take specific actions to achieve compliance with funder and institutional open access

mandates. If your research is supported by a funder that requires immediate open access (e.g. according to Plan S principles or the NIH public access policy) then you should select the gold OA route, and we will direct you to the compliant route where possible. Because authors warrant under our subscription licensing terms that they haven't committed to licensing any version of their article under a licence inconsistent with the terms of our agreement – including the applicable embargo period – publication under the subscription model isn't suitable for authors whose funders require no embargo.

To assist our authors in disseminating their research to the broader community, our SharedIt initiative provides you with a unique shareable link that will allow anyone (with or without a subscription) to read the published article. Recipients of the link with a subscription will also be able to download and print the PDF.

If your paper includes color figures, please be aware that in order to help cover some of the additional cost of four-color reproduction, Nature Portfolio charges our authors a fee for the printing of their color figures. Please contact our offices for exact pricing and details.

As soon as your article is published, you will receive an automated email with your shareable link.

If you have not already done so, we strongly recommend that you upload the step-by-step protocols used in this manuscript to protocols.io (https://protocols.io), an open online resource that allows researchers to share their detailed experimental know-how. All uploaded protocols are made freely available and are assigned DOIs for ease of citation. Protocols and Nature Portfolio journal papers in which they are used can be linked to one another, and this link is clearly and prominently visible in the online versions of both. Authors who performed the specific experiments can act as primary authors for the Protocol as they will be best placed to share the methodology details, but the Corresponding Author of the present research paper should be included as one of the authors. By uploading your Protocols onto protocols.io, you are enabling researchers to more readily reproduce or adapt the methodology you use, as well as increasing the visibility of your protocols and papers. You can also establish a dedicated workspace to collect your lab Protocols. Further information can be found at https://www.protocols.io/help/publish-articles.

You can use a single sign-on for all your accounts, view the status of all your manuscript submissions and reviews, access usage statistics for your published articles and download a record of your refereeing activity for the Nature Portfolio.

Nature Cell Biology encourages authors presenting evidence for cell, biological, molecular, and genetic interactions to consider communicating these findings using Biofactoid (https://biofactoid.org/). This tool helps users share a searchable representation of interactions (e.g. binding, gene expression, post-translational modification) between genes, gene products, or chemicals. Information added to Biofactoid, with author attribution, is shared on social media and public databases, such as Pathway Commons, where it can be discovered and analyzed in the context of a large and growing corpus of knowledge.

Please feel free to contact us if you have any questions.

With kind regards,

Daryl

Daryl Jason Verzosa David, PhD

Senior Editor, Nature Cell Biology
Advisory Editor, npj Biological Physics and Mechanics
Nature Portfolio

Heidelberger Platz 3, 14197 Berlin, Germany
Email: daryl.david@nature.com
ORCID: https://orcid.org/0000-0002-9253-4805

Click here if you would like to recommend Nature Cell Biology to your librarian
http://www.nature.com/subscriptions/recommend.html#forms

** Visit the Springer Nature Editorial and Publishing website at www.springernature.com/editorial-and-publishing-jobs for more information about our career opportunities. If you have any questions please click here.**

Open Access This Peer Review File is licensed under a Creative Commons Attribution 4.0 International License, which permits use, sharing, adaptation, distribution and reproduction in any medium or format, as long as you give appropriate credit to the original author(s) and the source, provide a link to the Creative Commons license, and indicate if changes were made.
In cases where reviewers are anonymous, credit should be given to 'Anonymous Referee' and the source.
The images or other third party material in this Peer Review File are included in the article's Creative Commons license, unless indicated otherwise in a credit line to the material. If material is not included in the article's Creative Commons license and your intended use is not permitted by statutory regulation or exceeds the permitted use, you will need to obtain permission directly from the copyright holder.
To view a copy of this license, visit https://creativecommons.org/licenses/by/4.0/

Point-by-point response to reviewer comments

Reviewer 1

Dynamic microtubules regulate eukaryotic cell polarity, in part by influencing F-actin polymerization dynamics and retrograde flow. However, the signaling pathways linking microtubule dynamics to F-actin regulation remain poorly understood. Rho GTPases, activated by guanine nucleotide exchange factors (GEFs) such as GEF-H1 (a Rho-specific GEF), modulate the actin cytoskeleton and myosin-driven contractility.

In this manuscript, Akhmanova and colleagues investigate how the microtubule-stabilizing drug Taxol (paclitaxel) and its derivatives affect GEF-H1 binding to microtubules, Rho activation, and subsequent F-actin cytoskeleton organization. The authors show that Taxol binding induces GEF-H1 dissociation from microtubules (likely via allosteric effects on the microtubule lattice), which potentially activates GEF-H1, alters F-actin organization, and changes cell shape. They support this with in vitro reconstitution assays demonstrating Taxol's impact on GEF-H1 localization, combined with in vivo cell biology experiments (primarily using fixed cells or fluorescently tagged actin/microtubules).

The manuscript is of interest to wide audiences, but the authors must revise the manuscript to address the experimental and conceptual concerns:

Strengths

1)The study integrates in vitro and in vivo approaches to address how microtubule stabilization by Taxol releases GEF-H1.

2)It provides novel insights into an unexpected mechanism: Taxol-induced microtubule lattice changes can displace GEF-H1, contrasting with the more typical activation by microtubule depolymerization.

3)The topic is broadly relevant to cytoskeletal regulation, Rho signaling, and the cellular effects of microtubule-targeting drugs used in cancer therapy.

Major Concerns

1)The manuscript focuses narrowly on the mechanistic effect of Taxol on GEF-H1 release but does not sufficiently address key biological questions, such as whether GEF-H1 release leads to its activation, and the downstream implications for cell motility (e.g., in fibroblasts) or long-term processes like cell division.

We are grateful to the reviewer for the thorough evaluation and positive comments about our manuscript. In this manuscript, we demonstrate that Taxol releases GEF-H1 from microtubules, and this leads to RhoA activation and actin remodeling, whereas these effects do not occur in GEF-H1 knockout cells. These data directly show that GEF-H1 release leads to activation of RhoA signaling, cell rounding and changes in the actin cytoskeleton.

Activatable RhoA is restricted to the plasma membrane, and this is why sequestration of GEF-H1 can be sufficient as an inhibitory mechanism. The activation of GEF-H1 by its release from microtubules has already been the focus of several studies: Krendel et al. Nat Cell Biol 2002, doi: 10.1038/ncb773; Meiri et al., Mol Cell 2012, doi: 10.1016/j.molcel.2012.01.027; Choi et al, Cell 2025, doi: 10.1016/j.cell.2025.11.011; Kamps et al. Cell Rep 2020, doi: 10.1016/j.celrep.2020.108467. Please note that in the Kamps et al. study, it was directly demonstrated that sequestration of a non-microtubule binding mutant of GEF-H1 on mitochondria is sufficient to tune down the activity of GEF-H1 and Rho A. This shows that sequestration away from the plasma membrane is sufficient to control

GEF-H1 activity, and while this is normally regulated by microtubules, no complex microtubule dependent activation steps appear to be needed.

The effects of Taxol on cell division and migration are generally accepted, and it was not our goal to reproduce extensive, previously published studies describing these phenomena. Specifically, mitotic arrest caused by spindle defects and the activation of the spindle assembly checkpoint, as well as chromosome missegregation, are the best understood mechanisms of Taxol action, known for more than 30 years (e.g., Jordan et al., PNAS 1993, doi: 10.1073/pnas.90.20.9552; Terzis et al Brit J Cancer 1997, doi: 10.1038/bjc.1997.298) and extensively reviewed over the years: see, for example, Jordan and Wilson, Nat Rev Cancer 2004, doi: 10.1038/nrc1317; Weaver, Mol Biol Cell 2017, doi: 10.1091/mbc.e14-04-0916. Inhibition of cell migration by Taxol treatment has also already been reported previously. The effects of Taxol on polarized morphology of migrating fibroblasts have already been described by Tomasek and Hey in 1984 (doi: 10.1083/jcb.99.2.536). A frequently cited study on the effects of microtubule-targeting agents on cell migration and division is Yang, Ganguli and Cabral, J Biol Chem 2010 (doi: 10.1074/jbc.M110.160820). Another frequently cited study demonstrating inhibition of cell migration by Taxol is Hellal et al, Science 2012; doi: 10.1126/science.1201148. Importantly, despite these observations, the exact mechanisms by which Taxol exerts its effects were not understood. We now demonstrate that Taxol-induced release of GEF-H1 from microtubules activates RhoA and thereby increases cell contractility, which is a novel finding and the focus of the current paper.

2)Cell biology data rely heavily on fixed samples or static imaging of fluorescently tagged actin/microtubules; this limits insights into dynamic processes.

To address this comment, we have now added live cell imaging of MDA-MB-231 breast cancer cells expressing Lifeact-mCherry, 1205Lu melanoma cells expressing eGFP-Lifeact, and HT1080 fibrosarcoma cells expressing F-tractin-mStaygold to demonstrate the dynamic response of these cells to Taxol (Videos 1-6). The paper now also includes videos demonstrating release of GEF-H1 from microtubules by Taxol treatment in both U2OS and HT1080 cells (Videos 7-10).

3)The study equates GEF-H1 binding to microtubules with inactivation but does not explore the multi-domain nature of GEF-H1 or potential autoinhibitory mechanisms beyond simple sequestration.

Autoinhibition of GEF-H1, including the generation of fluorescent sensors reporting the activation of GEF-H1, has been investigated by Azoitei et al, J Cell Biol 2019 (doi: 10.1083/jcb.201812073). This paper showed that microtubule binding through the N-terminal C1 domain sequesters GEF-H1 on microtubules and renders it inactive. Moreover, it was recently shown that point mutations perturbing the interface between the microtubule-binding C1 domain of GEF-H1 and microtubules are sufficient to activate GEF-H1 and the downstream RhoA signaling also in the physiological context of immune cells (Choi et al, Cell 2026, doi: 10.1016/j.cell.2025.11.011). The same study further demonstrated that, although the catalytic DH–PH domain of GEF-H1 adopts a compact quaternary structure similar to that of the well-characterized GEF VAV1, the C1 domain of GEF-H1 does not engage in a stable intramolecular interaction with the DH–PH domain to form a defined autoinhibited conformation. Rather, the C1 domain appears to act as an independent module. Finally, as mentioned above, it has been demonstrated that GEF-H1 sequestration on mitochondria, away from the plasma membrane, is sufficient to inhibit its ability to activate RhoA (Kamps et al. Cell Rep 2020, doi: 10.1016/j.celrep.2020.108467). While these results do not exclude additional mechanisms controlling the multidomain GEF-H1 molecule, such as regulatory phosphorylation by different kinases, they support the established notion that release from microtubules is a key mechanism for GEF-H1 activation.

Our study focuses on addressing this mechanism from the microtubule perspective. We show that conformational change in the microtubule lattice is sufficient to release full-length GEF-H1 (or its microtubule-binding inhibitory C1 domain alone) and activate Rho A signaling. This novel concept is the main point of our study.

4)The discussion largely overlooks the native biological role of microtubule-associated GEF-H1 autoinhibition in regulating F-actin dynamics and cell motility. It does not adequately discuss physiological signals (e.g., native substrates or microtubule depolymerization) that release GEF-H1 in normal cells, nor does it frame Taxol's effects in the context of perturbing a native pathway. Instead, the discussion emphasizes Taxol's cancerous/cellular effects without sufficient grounding in endogenous regulation.

The native context of GEF-H1 activation by release from microtubules has been addressed by previous studies. It has already been convincingly demonstrated that GEF-H1 acting downstream of dynamic microtubules controls cell migration and matrix invasion (Nalbant et al. Mol Biol Cell 2009, doi: 10.1091/mbc.e09-01-0041; Heck et al. Mol Biol Cell 2012; doi: 10.1091/mbc.E11-10-087). Azoitei et al (J Cell Biol 2019, doi: 10.1083/jcb.201812073) analyzed in detail how local changes in the microtubule cytoskeleton regulate GEF-H1 and RhoA activity at the cell edge. In another study, Kopf et al (J Cell Biol 2020, doi: 10.1083/jcb.201907154) demonstrated that local microtubule depolymerization within protrusions triggers the contraction and retraction, and this process is important for cell coherence. These studies established the importance of dynamic microtubules in the regulation of GEF-H1.

Our work adds an important mechanistic insight, because it explains why GEF-H1 is biased to dynamic microtubules (which are compacted) instead of being sequestered on stable microtubules (which are expanded, de Jager et al., J Cell Biol 2025; doi: 10.1083/jcb.202206143). This concept is novel and important, because it introduces the notion that different microtubule subsets can differentially regulate signaling pathways. Avoidance of stabilized microtubules can prevent GEF-H1 sequestration on the stable perinuclear microtubule pool and bias it towards dynamic microtubules in protrusions to regulate their retraction. Furthermore, stabilization of microtubules in the vicinity of focal adhesions can trigger GEF-H1 release and RhoA-dependent focal adhesion disassembly during cell movement. Taxol treatment, which expands microtubules, extends this effect to all microtubules in the cell, causing global RhoA activation. As suggested by the reviewer, we extended and emphasized this reasoning better in the Discussion of the revised paper on page 10.

Specific Recommendations for Revision

1.Enhance biological impact studies with live cell imaging of motile and dividing cells: A)Perform live-cell imaging to assess the effects of Taxol on cell motility (e.g., migration assays in fibroblasts) and cell division.

As outlined above, we feel that cell motility or cell division assays would not add any novelty to the paper, since such experiments with Taxol have already been extensively published, and it is established that Taxol inhibits both processes.

B)Conduct additional live imaging to directly examine the dynamics of f-actin mediated Rho activation downstream of GEF-H1 release from microtubules.

We have now conducted live imaging experiments to visualize F-actin and included them in the new Supplemental Videos 1-6. These data show that Taxol addition causes fast cell rounding and blebbing

response, followed by slight respreading of the cell and formation of prominent stress fibers, while no striking difference was observed upon treatment with discodermolide or DMSO.

2.Investigate GEF-H1 autoinhibition mechanism:

A) Explore structural homology models comparing GEF-H1 to other GEFs, focusing on how microtubule binding inhibits GEF activity and how release induces activation.

B) Test mutants that disrupt putative autoinhibitory domains or the microtubule-binding site to determine whether microtubule-bound GEF-H1 can be active and how this affects the F-actin cytoskeleton in cells.

As outlined above, there is already very strong published evidence, including rational point mutations in the C1 domain of GEF-H1 (Choi et al, Cell 2026, doi: 10.1016/j.cell.2025.11.011), supporting the GEF-H1 inactivation mechanism based on sequestration on microtubules. Comparison with other GEFs, e.g. VAV1, and biochemical experiments based on this comparison are published in Azoitei et al, J Cell Biol 2019, doi: 10.1083/jcb.201812073 and Choi et al, Cell 2026, doi: 10.1016/j.cell.2025.11.011. Repeating these experiments would not add novelty to our study, which focused on GEF-H1 regulation by changes in microtubule lattice conformations.

3.Revise and expand the discussion:

A)Prioritize the native biological pathway: Discuss the physiological role of coupling GEF-H1 auto-inhibition to microtubules in regulating F-actin dynamics and key processes like cell motility.

We have now modified the Discussion to emphasize better the physiological role of the regulation of GEF-H1 by microtubule lattice conformation in the context of microtubule stabilization and generation of different microtubule subsets, which is the key novel point of our study.

B)Address open questions: Are there endogenous signals/substrates that release GEF-H1 from microtubules? Is microtubule depolymerization the primary physiological trigger for GEF-H1 activation?

Our work suggests that microtubule stabilization which correlates with lattice expansion may serve as a trigger for GEF-H1 activation, along with microtubule depolymerization. We emphasized this point more clearly in the Discussion on page 10.

C)Reposition Taxol effects as perturbation of this native pathway, rather than treating them as the primary focus.

As suggested by the Reviewer, in the revised manuscript we have expanded the Discussion on the role of microtubule lattice conformation in controlling the native activity of GEF-H1. However, we do believe it is also appropriate to do justice to the strong general interest in the mechanisms underlying Taxol action and its side activities in cancer therapies, because taxanes remain widely used for the treatment of the most common cancers worldwide, and understanding their mechanisms of action is of considerable clinical importance for therapy optimization (see also reviewer 3).

Reviewer 2

The manuscript by Meiring et al addresses a long-standing question in cell biology (how is RhoA activated by changes in microtubules) by studying an emerging idea in the microtubule field (that the conformation of tubulin subunits within the microtubule lattice is locally written and read as part of the tubulin code). Over 20 years ago, it was shown that microtubule-destabilizing agents activate RhoA by releasing GEF-H1 from microtubules. With the emerging ideas in the microtubule field, Meiring et al asked whether conformational changes in tubulin subunits in the lattice can also release GEF-H1 and activate RhoA. The authors find that treating cells with paclitaxel, with causes tubulin to adopt an extended conformation, causes activation of RhoA signaling and release of GEF-H1 from microtubules and that the effects are dependent on GEF-H1. They then reconstitute GEF-H1 binding to microtubules and show a preference for the compacted state. This is a really strong paper, the results are exciting, and the information is timely although a few control experiments and some text revisions would strengthen and clarify the manuscript.

We thank the reviewer for the positive comments on our manuscript.

1) Why HT1080 fibrosarcoma cells? It would strengthen the findings if the authors could repeat key experiments in cells commonly used to investigate Rho signaling such as mouse embryonic fibroblasts, U2OS, or NIH3T3 cells. Especially since GEF-H1 KO cells have higher FBS response, there may be quite a bit of variability between cell lines and it would be good to see whether the effects are reproducible. Maybe it's too much with the Rho FRET sensor but at least repeating experiments with the stress fibers and GEF-H1 release in another cell line.

We initially used HT1080 cells because they are a very good human cell model to study cell migration not just in 2D but also in 3D settings, and generation of a GEF-H1 knockout in this background seemed like a good investment. However, the effects can be observed in different commonly used cell lines, and we have now added F-actin, cell circularity and p-MLC quantifications for Swiss 3T3 and U2OS cells (Figure S2), as well as movies of GEF-H1 release in HT1080 and actin remodeling in HT1080, MDA-MB-231 breast cancer cells, and 1205 Lu melanoma cells (Video 3-10).

2) I don't understand the FChitax-3 experiments. The impact on RhoA activation is not measured directly and these experiments were done with a different drug in different cell line at different conc than paclitaxel and require a much longer response time. Also, according to Lucena-Agell et al (2025), FChitax3 treatment should result in a compacted microtubule lattice.

The purpose of FChitax-3 experiments was to demonstrate the gradual and persistent accumulation of taxanes in cells when utilized at nM concentrations. Slow accumulation of taxanes in interphase cells explains the gradual activation of RhoA by 100 nM docetaxel shown in Figure 1f. We have now added a better explanation to the main text: "The idea that taxanes can accumulate in cells gradually at low concentrations was supported by 7-hour long live imaging of cell accumulation of a fluorescent taxane, Fchitax-3".

3) Since one of the points the authors want to make is that discodermolide is a lattice compactor that can be used as a control for paclitaxel treatment, the authors need to show that discodermolide does not activate RhoA. The FChitax3, if needed, could be moved to the supplement.

In order to address this, we have now added RhoA2G FRET data with Discodermolide (Fig 1a), and also added Discodermolide to the cell experiments quantifying F-actin intensity, cell circularity and p-MLC (new panels in Figures 2 & S2). These data show that Discodermolide does not activate RhoA.

4) Likewise, it would be good to show that discodermolide does not cause stabilization of filamentous actin and increased actomyosin contractility. Also, for Fig 2, FBS would be a better control since it activates RhoA signaling without GEF-H1.

We have now added both FBS and discodermolide to the F-actin quantification, cell circularity and p-MLC quantification experiments for HT1080, U2OS and Swiss 3T3 cells (Fig. 2 and S2). These results show that discodermolide does not cause stabilization of filamentous actin or increased actomyosin contractility.

5) For Fig 3, the authors rely on overexpression studies and state that they image cells at low levels, but no information or quantification is provided for how much overexpression there is, even for low levels of expression. For such important experiments, I am surprised the authors did not make a knockin cell line so they can look at GEF-H1 under endogenous conditions. At a minimum, they should show the effects of various treatments on GEF-H1 localization at different levels of expression. It seems likely that at high levels of expression, GEF-H1 can resist paclitaxel, perhaps by compacting the lattice (e.g. cell in top left corner of Fig 3F). The data in Fig 4P also suggest that GEF-H1 can compact a GMPCPP seed. Can the authors delve into this more fully, for example using experiments similar to Paquette et al 2025 where they vary the concentrations of GEF-H1 and paclitaxel.

Indeed, our in vitro experiments directly demonstrate that high concentrations of GEF-H1 can compact even a taxane-expanded lattice (Fig. 4p), showing that avoidance of Taxol-stabilized microtubules by GEF-H1 occurs only at sufficiently low GEF-H1 concentrations, and we observed a similar phenomenon in cells. We found that in U2OS cells, 2-10x endogenous levels GEF-H1 can still be displaced by Taxol, and this is the range we used for analysis. We have now added a figure to demonstrate this (new Fig. S3a, b). Above these levels, GEF-H1 cannot be displaced, because it enforces its own preferred conformation on microtubules. Note that published structural data from Choi et al, Cell 2026 (doi: 10.1016/j.cell.2025.11.011; illustrated in Fig. S6a, b) support this notion – the C1 domain alone, used to decorate microtubules in this cryo-EM study, was able to partially compact Taxol-stabilized lattices.

5) One point the authors want to make is that GEF-H1 avoids acetylated microtubules. Their results do support the hypothesis that GEF-H1 binding to microtubules is not dependent on PTMs but the relationship between PTM state and conformational state is unclear. Recent work in the field suggests that the tubulin expanded conformation can drive PTM (e.g. Yue et al 2023, Yue et al 2025, Egoldt et al 2025) but it is not clear whether overexpression of PTM enzymes and/or the modification drives tubulin to an expanded conformation. Indeed, if they overexpress alphaTAT to increase acetylation, then GEF-H1 is on all the microtubules, suggesting that acetylated microtubules could be compacted.

This is indeed correct. The conclusions that compacted microtubules can be acetylated and that acetylated microtubules can be compacted are also supported by Jansen et al. J Cell Biol 2023 (doi: 10.1083/jcb.202106105) demonstrating that over-acetylation of microtubules using deacetylase inhibitor tubacin doesn't alter the distribution of StableMARK, a rigor version of kinesin-1 that specifically recognizes expanded microtubules.

6) To fully support the hypothesis that the expanded tubulin state in cells releases GEF-H1, the authors should utilize methods that other papers have recently used such as stableMARK or a MAP or osmotic shock.

We have now added a figure panel with GEF-H1 and StableMARK showing that GEF-H1 avoids StableMARK labelled microtubules (new Fig. S3c).

7) The reconstitution experiments suggest that GEF-H1 (full length and truncation) prefers GTPgS > GDP > GMPCPP microtubules. Can the authors quantify this? If so, then this is consistent with GEF-H1 preferring a compacted lattice state. But then why doesn't GEF-H1 track plus ends in cells and in vitro? Note that the authors use GTPgS as a GTP analog, but recent work indicates that GTPgS is a compacted lattice.

We have added the suggested quantification experiments, which showed that GEF-H1 binds with similar affinity to GTPγS and GDP microtubules (this point has been corrected in the revised paper), consistent with the view that they are both compacted, even though GTPγS is a GTP analog, as correctly pointed out by the reviewer (Fig. S5c, d). Although both EBs and GEF-H1 prefer compacted lattice, GEF-H1 doesn't track growing microtubules. This is not surprising, because their binding sites are not the same: the CH domain of EBs binds at the vertex between 4 tubulin dimers, close to the beta-tubulin site affected by nucleotide state. In contrast, the C1 domain of GEF-H1 binds between two tubulin dimers, far away from this site. This is illustrated in Figure S6d and discussed on pages 9 and 10.

8) It is surprising that paclitaxel knocks both EB and GEF-H1 off of the lattice whereas discodermolide knocks off EB but not GEF-H1. Both EB and GEF-H1 prefer to bind to a GTPgS lattice and the dimer rise of a discodermolide lattice is similar to that of GTPgS lattice (Fig S4).

The recognition of growing microtubule plus ends by EBs relies not only on the discrimination between compacted and expanded lattices but also on the nucleotide state of beta-tubulin, and therefore, endogenous EBs are enriched at the growing plus ends, forming comets as shown in Fig. S3d. Discodermolide stabilizes microtubules, increases their number and reduces soluble tubulin pool, thereby inhibiting microtubule growth, and this is why EB comets disappear (GTP caps at microtubule plus ends become very short or are gone).

10) The authors state that discodermolide generates a compacted lattice based on cryoEM but there is no data backing up this claim. For Fig S4, the authors should state how they arrived at each point on the graphs (i.e. they need references).

We have clarified the text to make explicit the evidence supporting the classification of discodermolide-stabilized microtubules as having a compacted lattice.

We have two independent lines of evidence showing that discodermolide generates compacted lattices. First, in this manuscript, cryo-EM single-particle analysis yielded a dimer rise of 81.7 Å and 81.6 Å for 13-pf and 14-pf microtubules, respectively (the cryo-EM maps for the discodermolide stabilized 13-protofilament and 14-protofilament microtubules have been deposited in the EMDB under the accession codes EMD-58509 and EMD-58507, respectively; since these files are not released yet, the reviewers can find them at https://surfdrive.surf.nl/s/mkGpwRH3F9arQKR). These values fall within the compacted lattice range and is very close to the GTPγS values reported by Zhang et al, PNAS 2018 (doi: 10.1073/pnas.180263711). Importantly, this lattice spacing is clearly distinct from the expanded paclitaxel lattice.

Second, in Lucena-Agell et al. (bioRxiv, 2025.2011.2011.687833; recently accepted in PNAS, 2026; doi: 10.1101/2025.11.11.687833), an orthogonal fiber diffraction analysis also places discodermolide-stabilized microtubules in the compressed lattice class, with a monomer repeat of 4.05 ± 0.02 nm.

Therefore, the conclusion that discodermolide promotes a compacted lattice is supported by two independent structural approaches: cryo-EM single-particle analysis and fiber diffraction. We have also

revised the legend of Figure S6 to include the corresponding references for each data point shown in the graphs.

Minor comments:

1) Out of curiosity, are GEF-H1 mutations associated with cancer and or taxol resistance?

GEF-H1 is considered an oncogene and is often upregulated in cancer (Cheng et al. J Path 2012, doi: 10.1002/path.4084; Zhu et al. Cell Death Dis 2022,10.1038/s41419-022-05099-8). It has important roles in regulating RhoA during cell migration, hence it can also promote metastasis (Cheng et al. J Path 2012, doi: 10.1002/path.4084). GEF-H1 was also found to enhance the RAS-MAPK pathway (Cullis et al. Cancer Cell 2014, doi: 10.1016/j.ccr.2014.01.025). For these reasons GEF-H1 mutations probably do not benefit cancer cells. Indeed, mutations of GEF-H1 have not been reported as a mechanism for Taxol resistance, but mutations and isotype changes in beta tubulin that undermine the binding and impact of Taxol on microtubules have (Orr et al. Oncogene 2003, 10.1038/sj.onc.1206934). We included some of this information in the Introduction of the revised paper.

2) The authors state that paclitaxel expands lattice and reference Kellogg et al 2017. But the Kellogg et al manuscript states that paclitaxel does not expand the lattice if added after polymerization.

We agree that the effect of paclitaxel on microtubule lattice conformation can depend strongly on the experimental conditions, including buffer composition, polymerization protocol, nucleotide state, stabilizing conditions, and the timing of drug addition.

To address this point more directly, we have now added a reference to Lucena-Agell et al. (bioRxiv, 2025.2011.2011.687833, recently accepted in PNAS, 2026, doi: 10.1101/2025.11.11.687833). This study provides, to our knowledge, the most comprehensive comparative analysis of microtubule lattice parameters in the presence of different microtubule-stabilizing agents. Importantly, Figure 4A of that work provides a direct measurement of the effect of paclitaxel on the lattice when added to pre-formed microtubules, showing that the lattice change occurs immediately. In addition, Table S2 of that paper reports the lattice parameters of pre-polymerized microtubules exposed to Taxol.

Therefore, while we acknowledge the observation reported by Kellogg et al. 2017, we believe that the available data indicate that paclitaxel-induced lattice expansion after polymerization is condition-dependent rather than absent as a general rule. In our own study, the data shown in Figure 4 provide direct and, in our view, convincing evidence that paclitaxel expands the microtubule lattice under the experimental conditions used here.

3) The authors show that activation of RhoA via FBS is not dependent on GEF-H1. What is known about how FBS does activate RhoA signaling? This would help the reader interpret the results.

Lysophosphatidic acid (LPA) in FBS binds receptors which in turn activate downstream RhoA signaling, but these pathways are complicated and are not yet fully understood, so reviewing them would be distracting. We added one sentence on page 5 stating that effects of FBS on RhoA activation are GEF-H1 independent.

4) For Fig 1, what are the authors' thoughts on needing such high levels of paclitaxel if clinical levels are nM and they see response at uM levels

Several factors should be considered when comparing the concentrations used in our experimental system with clinical exposure levels.

First, paclitaxel has slightly lower affinity and markedly lower aqueous solubility than docetaxel, another taxane frequently used in the clinic. Although the difference in affinity is modest — approximately two-fold, as reported by Díaz and Andreu, 1993 (doi: 10.1021/bi00062a003), and Buey et al., 2005 (doi: 10.1016/j.chembiol.2005.09.010) — this difference, together with the poorer solubility of paclitaxel, may contribute to the requirement for higher apparent concentrations in our assay. Consistent with this, when using docetaxel we observe clear effects at 100 nM.

Second, our data show that docetaxel-induced RhoA activation and intracellular taxane accumulation in interphase cells increase progressively over time. Importantly, this is also likely to be relevant in patient tissues, where the majority of cells are in interphase and do not divide.

Finally, as indicated in the manuscript, therapeutic taxane doses are relatively high, and plasma concentrations can reach the micromolar range, around 1 µM. Therefore, although the concentrations required to observe paclitaxel effects in Fig. 1 are higher than nominal nanomolar clinical values, they remain within a pharmacologically relevant range, particularly when considering drug solubility, intracellular accumulation, exposure time, and the behavior of non-dividing interphase cells.

5) For Fig 2, the authors use actin as gel loading control but they are measuring changes in actin fluorescence intensity in response to the treatments. It looks in the gel that actin changes in response to paclitaxel (like in the intensity measurements) so a different loading control for the western blot is needed.

The stainings were performed with phalloidin, which specifically binds polymerized actin also known as filamentous actin (F-actin), while when blotting for actin on a Western blot, the total actin levels are detected, and these, unlike the F-actin to G-actin ratio, would not change.

6) Can the authors comment on the differences between nocodazole and paclitaxel in terms of RhoA in Fig 1A vs actin and circularity in Fig 2? The treatments in Fig 1A result in very different RhoA activation profiles in terms of time of increase and whether the signal decays over time. At 30 min, RhoA is still increasing in nocodazole treatment where it has almost returned to baseline in paclitaxel. Yet both treatments give the same response in Fig 2. If a short Rho activation is sufficient, then stimulating with FBS should give the same results in Fig 2.

In the case of FBS, a short treatment is indeed sufficient to induce stress fibers. We have now added FBS treatments to the quantifications of F-actin intensity and circularity (Fig 2a-f, S2a-f). We believe that the RhoA FRET data is a more sensitive method to quantify temporal differences in signaling intensity than downstream effects quantified by F-actin intensity, cell morphology and p-MLC, which depend on the kinetics of activation and inactivation of additional signaling intermediates, such as ROCK and formin.

7) For the graphs with individual data plotted, the different shapes and colors are not needed and the yellow x is very difficult to see. It would be better to use black spots for every plot and then a bright color for the averages. Are the statistics done comparing all the spots or the averages? Also, please show 95% confidence intervals rather than SD.

We have now used grey dots for the individual cells and differently colored X for the average according to the treatment and added 95% confidence intervals. We believe this use of a different color for each treatment allows the reader to quickly connect different experiments with the same treatment type across panels over the same figure. The statistics are performed on the individual cell measurements rather than the experimental averages.

8) For the experiments where they overexpress detyrosination enzymes (Fig S2), it would be better to stain for total tubulin (something that is not changing) instead of Tyr-tubulin (which is decreasing).

Where we saw the option of using two controls (tyrosinated tubulin and detyrosinated tubulin antibody) we used both to strengthen our conclusion. Staining for detyrosinated tubulin alone does not indicate the extent of microtubule detyrosination. Since microtubules are tyrosinated by default, the absence of tyrosinated tubulin staining demonstrates that the microtubule population is fully detyrosinated.

9) In Fig 3G, the images do not match up.

Thank you for spotting this, we apologize for the mistake. We have now fixed this panel.

10) What are red and green arrows in Fig 4A?

These are excitation and emission wavelengths, we have now added extra annotations to the figure legend to indicate this.

Reviewer 3.

The study by Meiring et al. investigates how effects of different microtubule-targeted drugs are linked to activation of RhoA-dependent signalling. This is a highly relevant question in the context of cancer chemotherapy where the therapeutic effects of such drugs are still incompletely understood. There is strong evidence that interference with mitotic spindle assembly and function is not the only therapeutically relevant effect, and that additional non-mitotic effects such as activation of Rho-dependent pathways play a role. This could modulate cancer cell survival through reduced adhesion, immune cell function, or blood vessel sprouting.

Based on previous work showing that sequestration of the RhoA activator GEF-H1 through binding to microtubule lattices inhibits Rho signalling, the authors show that, somewhat counter-intuitively, not only depolymerization of microtubules, but also stabilization by taxanes results in GEF-H1 release and Rho pathway activation. The authors then reveal the molecular basis of this effect, showing that GEF-H1 recognizes tubulin spacing in the microtubule lattice, which is increased through microtubule lattice expansion by taxanes, leading to GEF-H1 release, despite the stabilization and presence of microtubule lattices. For this they use cell-based assays to demonstrate Rho pathway activation and GEF-H1 microtubule interaction and also reconstitute the effects in vitro using TIRF imaging of GEF-H1 interaction with compacted and expanded microtubule lattices obtained with various microtubule binding compounds. Finally, as additional control for their model, they use cryo-EM and structural analyses to determine that discodermolide, while also stabilizing microtubules, compacts lattices and does not trigger GEF-H1 release.

This very well-executed and presented study closes an important gap in our understanding of the cellular effects of microtubule-targeted drugs used in chemotherapy. It highlights the differential effects of these drugs on activation of Rho signalling, which may be an additional therapeutic benefit, opening up new routes for further investigation and drug development in the future. Apart from this, the work also establishes microtubule lattice conformation as an important regulatory mechanism that, together with posttranslational modifications, could be part of the tubulin "code" that is read by microtubule interacting proteins.

This is a very nice paper, presenting high quality data to support the conclusions.

We thank the reviewer for the supportive comments on our manuscript.

I have found only one issue that needs to be addressed:

There is a disconnect between cellular data in the first part on Rho pathway activation and contractility (Fig. 1 and 2) and the second part on the underlying mechanism analysing microtubule conformations and GEF-H1 binding to microtubules (Fig. 3). The disconnect stems from the switch from HT1080 cells in the first part to U2OS in the second part. The authors justify this with better microtubule imaging in U2OS due to their stronger adherence and flattened morphology. But this also means that there is no demonstrated effect on Rho activation in these cells (e.g. no cell rounding, increased contractility). Similarly, in the experiments with HT1080 cells, no GEF-H1-microtubule binding/release is shown. Thus, the current data are based on correlation of two different effects obtained independently in two different cell lines (Rho pathway signalling vs. lattice state and GEF-H1 interaction). I strongly suggest that the authors demonstrate a direct effect in the same cell line of lattice spacing induced by a specific drug on GEF-H1 binding state, and on Rho-dependent signaling/contractility. This could be done by analyzing microtubules in HT1080, perhaps using an adherence-promoting substrate, or by analyzing Rho activity and contractility in U2OS cells. Alternatively, another suitable cell line could be used. Without such data, the support for the main message of this paper would be significantly weaker.

We fully agree with this comment. We have now added F-actin, circularity and p-MLC quantifications in U2OS cells (Fig. S2b, d, f, h, j), as well as live imaging of actin in MDA-MB-231 breast cancer cells, 1205Lu melanoma cells and HT1080 cells, and paclitaxel wash-in assays looking at GFP-GEF-H1 in HT1080 cells (Video 9, 10). These experiments showed that paclitaxel mediated GEF-H1 displacement and RhoA activation is a widespread phenomenon, which is not restricted to one cell type.

Point-by-point response to reviewer comments

Reviewer #1 (Remarks to the Author):

The authors have addressed all the concerns raised by the reviewers in the revised manuscript.

Reviewer #2 (Remarks to the Author):

The revised manuscript by Meiring et al has added new text and experimental evidence that clarify and/or support their findings. The main point is that the sequestraiton of GEF-H1, the major RhoA activitor, on microtubules is regulated by the conformational state of the microtubule. This finding provides a mechanism for the fact that both microtubule depolymerization and stabilization activate RhoA. The experiments are well-done and compelling, the findings are exciting and foundational, and the work is timely and important.

Reviewer #3 (Remarks to the Author):

The authors have done a very thorough revision. The issue that I raised was addressed and I have no further concerns.

We thank the reviewers for the constructive and positive assessment of our manuscript,
